# MSN/STAT3 drives cancer stemness and chemoresistance via IL-6/LPAR1 ligand receptor complex in triple-negative breast cancer

**DOI:** 10.1186/s13058-025-02072-z

**Published:** 2025-07-22

**Authors:** Cheng Hyun Lee, Soo Young Park, Jae Seok Lee, Da Sol Kim, Ha Yeon Kim, Min Ji Song, Seock-Ah Im, Kyung-Hun Lee, Dae-Won Lee, Ilias P. Nikas, Ji Won Koh, So Hyeon Yang, Hyebin Lee, Han Suk Ryu

**Affiliations:** 1 Department of Pathology, Seoul National University College of Medicine, Seoul, Republic of Korea; 2 Department of Pathology, Seoul National University Hospital, Seoul National University College of Medicine, 101 Daehak-Ro, Seoul, Jongno-Gu 03080 Republic of Korea; 3 Center for Medical Innovation, Biomedical Research Institute, Seoul National University Hospital, Seoul, Republic of Korea; 4 Pharmonoid Co., Ltd, Seoul, Korea; 5 Department of Pathology, Samsung Changwon Hospital, Sungkyunkwan University School of Medicine, Changwon, Republic of Korea; 6 Department of Internal Medicine, Seoul National University Hospital, Seoul National University College of Medicine, Seoul, Republic of Korea; 7 Translational Medicine, Seoul National University College of Medicine, Seoul, Republic of Korea; 8 Cancer Research Institute, Seoul National University, Seoul, Republic of Korea; 9 Medical School, University of Cyprus, Nicosia, Cyprus; 10 Department of Radiation Oncology, Kangbuk Samsung Hospital, Sungkyunkwan University School of Medicine, Gu29, Saemunan-Ro, Seoul, Jongno-Gu 03181 Republic of Korea

**Keywords:** Adriamycin resistance, Atovaquone, Cancer stemness, Moesin (MSN), Triple-negative breast cancer (TNBC)

## Abstract

**Background:**

Resistance to chemotherapy remains a major clinical challenge in triple-negative breast cancer (TNBC), an intrinsic subtype with limited available therapeutic options. The expression of moesin (MSN) is upregulated in TNBC patients, but little is known about the role of MSN in breast carcinogenesis.

**Methods:**

We investigated the MSN-dependent autocrine loop between extracellular interleukin 6 (IL-6) and NF-κB, along with a signaling cascade involving GTPase-mediated STAT3 phosphorylation. Various in vitro and in vivo assays were used to evaluate tumor initiation, growth, and stemness properties in TNBC models.

**Results:**

High MSN expression was correlated with shorter overall and disease-free survival in TNBC patients. In vivo, MSN promotes tumor initiation and growth. Mechanistically, MSN-mediated IL-6/NF-κB autoregulatory feedback enhances IL-6 transcription. IL-6 binding to LPAR1 activated MSN phosphorylation, which then sequentially phosphorylated the CDC42-PAK4 complex, triggering nuclear translocation of the pSTAT3-MSN complex. This led to pSTAT3-mediated activation of cancer stemness genes (IGFN1, EML1, and SRGN), contributing to Adriamycin resistance. Notably, combination treatment with the FDA-approved STAT3 inhibitor Atovaquone and Adriamycin restored drug sensitivity.

**Conclusions:**

Our findings uncover the critical role of MSN in regulating STAT3-mediated cancer stemness via the IL-6/NF-κB signaling axis. These results provide a strong rationale for repositioning STAT3 inhibitors such as Atovaquone as a therapeutic strategy in Adriamycin-resistant TNBC patients exhibiting pSTAT3-MSN complex upregulation.

**Supplementary Information:**

The online version contains supplementary material available at 10.1186/s13058-025-02072-z.

## Background

Triple-negative breast cancer (TNBC) is an aggressive intrinsic subtype of breast cancer, phenotypically displaying a higher proliferation index and metastatic potential compared to the other breast cancer subtypes. Due to the lack of hormone receptor expression and human epidermal growth factor 2 (HER2) amplification in TNBC, the therapeutic modalities available are limited to cytotoxic chemotherapy regimens involving platinum salts, anthracyclines, and taxanes. However, the treatment response rates remain low [1]. Therefore, it is necessary to identify key molecules and pathways inducing chemo-resistance and novel therapeutic strategies increasing chemo-sensitivity in TNBC. Since the six hallmarks of cancer were proposed, the interaction of cancer stem cells with the tumor microenvironment has been shown to play an integral role in cancer evolution [2]. At the same time, numerous aberrant signaling cascades lead to cancer stemness phenotypes with therapeutic resistance [3, 4]. Several oncogenic signaling pathways, including Janus kinases-signal transducer and transcription activator (JAK-STAT), govern cancer stem cells (CSCs) and the binding of various ligands, such as interleukins, to the respective receptors of JAK/STAT [5, 6]. Activated JAK phosphorylates non-receptor tyrosine kinases essential for signaling in response to cytokines and sequentially phosphorylates STAT3; this mechanism leads to poor chemotherapeutic response in several cancers [7–9]. We recently discovered a new oncogenic cytoskeletal adaptor protein, moesin (MSN), in our previous proteomics analysis [10]. The protein structure of MSN includes the N-terminal FERM domain, interacting with transmembrane receptors binding to the ligand such as an interleukin or growth factor, and the C-terminal F-actin binding domain interacting with the cytoskeleton in the cytoplasm [11]. As a protein connecting the cytoskeleton to the cell membrane, MSN regulates cell proliferation, motility, and intracellular signal transmission. Interestingly, the identical FERM domain is shared with other proteins, of which JAK, a protein implicated in chemotherapy resistance, is a notable example [12, 13]. These structural similarities prompt further investigation into the potential role of MSN as a novel signaling molecule triggering a key downstream pathway controlled by JAK, whose aberrant activation could confer resistance to chemotherapy. Our research provided compelling evidence that the nuclear localization of MSN promotes the binding of NF-κB into the promoter region of interleukin-6 (IL-6), inducing the transcription of IL-6 and enhancing an autoloop feedback of IL-6 stimulation in TNBC. Along with the interleukin and NF-κB circuit, IL-6 binds to G protein-coupled receptors (GPCRs), which sequentially activate the MSN-induced phosphorylation of STAT3 that is then translocated into the nucleus. Phosphorylated intranuclear STAT3 functions as a transcription factor for breast cancer stemness genes, inducing chemo-resistance in TNBC. Finally, we demonstrated that co-treatment with a STAT3 inhibitor and Adriamycin (ADR) represents a promising therapeutic strategy for restoring sensitivity to ADR in TNBC cells, in which resistance is mediated by the pSTAT3–MSN complex. Importantly, our study reveals a mechanistically novel role for MSN as a scaffolding partner that facilitates pSTAT3 nuclear translocation and activates downstream transcription of cancer stemness-related genes. To our knowledge, this is the first demonstration of such a function for MSN in the context of TNBC.

## Materials and Methods

### Cell Culture and Reagents

Human breast cancer cell lines (MDA-MB-231, RRID:CVCL_0062; Hs578T, RRID:CVCL_0332; BT20, RRID:CVCL_0178; MDA-MB-468, RRID:CVCL_0419; and MCF7, RRID:CVCL_0031) were obtained from the Korean Cell Line Bank (Seoul, Korea). Additionally, MCF7 cell lines resistant to Adriamycin (MCF7/ADR^r^), tamoxifen (MCF7/TAM^r^), and paclitaxel (MCF7/PAX^r^) were generously provided by Professor Woo-Kyung Moon from Seoul National University (Seoul, Korea). HEK293T cells (RRID:CVCL_0063) and 4T1 cells (RRID:CVCL_0125) were kindly provided by Professor Woo-Kyung Moon and Professor Jeong-Yeon Lee from Hanyang University (Seoul, Korea), respectively.

MDA-MB-231, Hs578T, MDA-MB-468, 4T1 and MCF7 cells were maintained in Dulbecco’s Modified Eagle Medium (DMEM) with high glucose, supplemented with 10% (v/v) heat-inactivated fetal bovine serum (FBS; Gibco, Rockford, IL, USA) and 1% penicillin–streptomycin (10,000 units/ml of penicillin and 10,000 μg/ml of streptomycin; Gibco). BT20 cells were cultured in RPMI-1640, supplemented with the same concentration of FBS and penicillin–streptomycin. All cells were maintained in a humidified incubator at 37 °C under a 5% CO_2_ atmosphere and passaged at 70–80% confluency. Mycoplasma contamination was routinely tested using the CycleavePCR Mycoplasma Detection Kit (Takara Bio, Shiga, Japan). Adriamycin was obtained from Sigma-Aldrich (St. Louis, MO, USA) for use in breast cancer cells. The STAT3 inhibitor Atovaquone (Selleckchem, Houston, TX, USA) and recombinant IL-6 (PeproTech, Cranbury, NJ, USA) were used in downstream experiments.

### Transfection of siRNAs and Plasmids

Small interfering RNAs (siRNAs) targeting the genes used in this study were obtained from Bioneer (Daejeon, Korea) and are listed in Supplementary Table S1. For transient target gene knockdown, siRNAs were transfected into appropriate cells for 48 h using Lipofectamine™ RNAiMAX Transfection Reagent (Invitrogen, Carlsbad, CA, USA), according to the manufacturer’s instructions. To produce lentivirus harboring the target gene and for luciferase assays, plasmids were transfected into HEK293T cells and the appropriate cells for 48 h, respectively, using Lipofectamine™ 2000 Transfection Reagent (Invitrogen).

### Lentivirus production and stable cell line establishment

For MSN overexpression, lentiviral vector Lv105 containing human full-length MSN cDNA or empty vector control was obtained by GeneCopoeia (Rockville, MD, USA, Product no. EX-D0144-Lv105). Conversely, for MSN knockdown, the lentiviral vector pGIPZ harboring small hairpin RNAs (shRNAs) targeting MSN or an empty control vector was obtained from Dharmacon (Lafayette, CO, USA, Product no. VGH5526-EG4478).

To generate the lentivirus encoding MSN cDNA or shRNAs, each lentiviral vector was co-transfected with packaging plasmids psPAX2 (RRID:Addgene_12260) and pMD2.G (RRID:Addgene_12259) into HEK293T cells using Lipofectamine ™ 2000 Transfection Reagent (Invitrogen). Following transfection, cells were incubated for 72 h, and the supernatant containing lentivirus particles was collected and filtered through 0.45-μm pore syringes. Target cells were seeded into appropriate culture vessels and incubated until reaching 50–70% confluency. The collected lentiviral supernatants were added to the target cells in the presence of 8 μg/ml polybrene (Sigma-Aldrich) to enhance transduction efficiency. After transduction, stable cell lines were selected by incubating cells with 2.5 μg/ml puromycin (Sigma-Aldrich) for a specified period until non-transduced cells were eliminated and stable transduced cells were obtained.

### Establishment of chemotherapy drug-resistant cell lines

For the MDA-MB-231 cell line, resistant cell lines to Adriamycin (ADR), cisplatin (CDDP), carboplatin (CBDCA), and paclitaxel (PTX) were developed through a stepwise process of drug exposure and selection. Parental MDA-MB-231 cells were subjected to escalating concentrations of each chemotherapy drug, ranging from 50 to 1000 nM for ADR, 5 μM to 50 μM for CDDP, 100 μM to 1000 μM for CBDCA, and 10 nM to 1000 nM for PTX. Surviving cells were cultured in media containing high doses of the respective drugs to amplify their populations. Following this, limiting dilution was employed to isolate individual resistant cell clones. Successful resistant cell clones against each drug were selected and characterized for their resistance profiles. Hereafter, ADR^r^, CDDP^r^, CBDCA^r^, and PTX^r^ represent the resistant cell lines.

### Patient selection

This retrospective study received approval from the Seoul National University Hospital Institutional Review Board, which also waived the requirement to obtain written informed consent from the patients (IRB no. H-2305–016–1428). To characterize the correlation between MSN expression and specific clinicopathologic parameters, a total of 181 formalin-fixed and paraffin-embedded (FFPE) tissue specimens from patients with TNBC who underwent surgical procedures at Seoul National University Hospital (SNUH) between 2003 and 2006 were included. For histological and immunohistochemical analysis, tissue microarrays (TMA) consisting of 2 mm cores from the cases above were constructed (Superbiochips laboratories, Seoul, Korea). The electronic medical records system was used to retrieve clinicopathologic parameters and patient survival data. These parameters included age, nuclear grade, histological grade, lymphatic invasion, lymph node metastasis, T stage, and vascular invasion, among others, according to the 8th edition of the American Joint Committee on Cancer (AJCC) [14]. For therapeutic response analysis based on MSN expression, core needle biopsy specimens from 86 TNBC patients who received single-regimen neoadjuvant chemotherapy (NAC) consisting of Adriamycin (ADR), cyclophosphamide (CPM), and docetaxel (DTX) were selected. These patients were divided into two groups based on their response: the complete remission (CR) group and the non-complete remission (nCR) group, based on the presence of residual invasive carcinoma in the post-operative surgical specimen [15]. The baseline characteristics of these patients are summarized in Supplementary Table S2. Additionally, the paired fresh-frozen tumor and surrounding normal tissue from 15 TNBC patients were analyzed to compare the expression of MSN between the two areas.

### Immunohistochemical staining

A 4-μm section from each TMA block was subjected to immunohistochemistry with MSN antibody (Santa Cruz Biotechnology, Cat# sc-58806, RRID:AB_784475) diluted at a ratio 1:250, using the BenchMark XT (Ventana Medical System, Inc., Tucson, AZ, USA). For core needle biopsy, immunohistochemistry was performed using the Bond-RXm system (Leica Biosystems, Wetzlar, Germany) with MSN antibody applied at a dilution of 1:50. For each case, the intensity score (IS) of each target was graded as follows: 0 (negative), 1 (weak), 2 (moderate), and 3 (strong). The proportion score (PS) was graded based on the percentage of cells at each IS grade, ensuring the total proportion added up to 100%. Specifically, the proportion scores for IS grades 0, 1, 2, and 3 were recorded as the percentage of cells exhibiting each respective intensity. The immunohistochemistry (IHC) results were calculated for each case using the following formula, ranging from 0 to 300: H-score = (IS_0 × PS_0) + (IS_1 × PS_1) + (IS_2 × PS_2) + (IS_3 × PS_3). In this formula, IS_0, IS_1, IS_2, and IS_3 represent the intensity grades of 0, 1, 2, and 3, respectively. Correspondingly, PS_0, PS_1, PS_2, and PS_3 represent the proportion scores for each intensity grade [16]. The cutoff for low and high expression of MSN was set at an H-score of 200. To enhance accuracy and reduce bias, all immunohistochemical staining was interpreted independently by experienced breast pathologists (J.S.L., I.P.N., and H.S.R.), with blinding applied during the analysis to prevent investigator bias. Any discordance among them was resolved through a consensus.

### In vivo Experiments

Five-week-old female NOD/SCID mice were purchased from KOATECH (Seoul, Korea). All experiments measured tumor size daily with calipers starting seven days after cell injection. Tumor volume was calculated using the formula: Tumor volume (mm^3^) = (*a* × *b*^2^)/2, where “*a*” and “*b*” represent the largest and perpendicular diameters, respectively. Animal experiments were conducted following the Institute for Experimental Animals College of Medicine guidelines and the Guide for the Care and Use of Laboratory Animals provided by the Institutional Animal Care and Use Committee of Seoul National University (IACUC approval no. SNU-220808–1–5). To determine the effect of MSN on breast cancer progression in an orthotopic mouse model, 5 × 10⁶ 4T1 cells stably expressing either shControl (shCON) or MSN shRNA were resuspended in 50% Matrigel (BD Corning, NY, USA) in cold PBS and injected into the mammary fat pads of the mice, which were randomly divided into two groups with six mice per group. Mice were sacrificed 24 days after cell injection, and primary tumors, lungs, and livers from each mouse were fixed in formalin and embedded in paraffin for histologic analysis. To measure in vivo tumor initiation ability, tenfold serially diluted MDA-MB-231 cells, expressing either shCON or MSN shRNA, were resuspended in Matrigel and orthotopically injected into mice. The frequency of tumor formation was analyzed using the L-Calc software (STEMCELL tech., Vancouver, BC, Canada).

### Western Blotting and Antibodies

Cell lysates were prepared using the Cell Signaling cell lysis buffer (Cell Signaling, Danvers, MA, USA) supplemented with protease and phosphatase inhibitors (Thermo Fisher Scientific, Waltham, MA, USA). 10 μg to 30 μg of protein was mixed with 5x SDS sample buffer and boiled at 95 °C for 5 min. Subsequently, proteins were separated by 8–14% polyacrylamide gels and transferred onto 0.45 μm Nitrocellulose membrane (Amersham Health, Stafford, United Kingdom). After transfer, the membranes were blocked with 5% nonfat skim milk in Tris-buffered saline with 0.1% Tween 20 (TBS-T; GeneAll, Seoul, Korea) and incubated overnight at 4 °C with primary antibodies. Following primary antibody incubation, membranes were washed and incubated with appropriate horseradish peroxidase-labeled secondary antibodies for 1 h at room temperature. Signal detection was performed using a Western ECL substrate (Thermo Fisher Scientific) and visualized using an Amersham 680 (Amersham). ImageJ 1.8.0 software (National Institutes of Health, Bethesda, MD, USA, RRID:SCR_003070) was used to quantify the relative expression of target proteins relative to the internal control GAPDH (Santa Cruz Biotechnology, Cat# sc-32233, RRID:AB_627679). Details of the specific antibodies used in this study are provided in Supplementary Table S3.

### Cell Proliferation and Viability Assay

Cells were seeded in 96-well cell culture plates for 2, 4, 6, and 8 days to measure cell proliferation. Each time, cells were lysed with CellTiter-Glo Luminescent Cell Viability Assay reagent (Promega, Madison, WI, USA) at a 1:1 ratio and incubated at room temperature for 12 min. The luminescence was measured using a microplate reader (Promega). Cells were seeded in 96-well cell culture plates or Ultra-Low clear round bottom plates to measure cell viability. Cells were incubated with and without the indicated treatment. After the specified periods, viability was measured using the CellTiter-Glo Luminescent Cell Viability Assay reagent for 2D attachment and the CellTiter-Glo 3D cell viability Assay reagent for 3D spheroid. The diameter of the 3D spheroid was calculated using ImageJ 1.8.0 software (NIH). The 50% inhibitory concentration (IC_50_) values were determined by nonlinear regression using a variable slope dose–response model and 95% confidence intervals with GraphPad Prism version 10.3.1 (GraphPad Software, Inc., La Jolla, CA, USA, RRID:SCR_002798). For drug combination effects, CompuSyn 1.0 software (ComboSyn, Inc., Paramus, NJ, USA) was used to calculate combination index (CI) values, where CI < 0.75 indicates synergism, CI = 0.75–1.25 indicates additive effects, and CI > 1.25 indicates antagonism.

### Transwell Migration and Invasion Assays

The cell migration and invasion abilities were evaluated using a transwell insert with an 8 μm pore size membrane (BD Corning). Cells were seeded in the upper chamber with a serum-free medium for the migration assay and incubated for 24 h. Migrated cells were fixed and stained with 0.1% crystal violet (Sigma-Aldrich), and images were captured under a light microscope. The number of migrated cells was counted. For the invasion assay, the upper chamber of the transwell insert was coated with a thin layer of 100ul of Matrigel (final concentration 2 mg/ml) to mimic the extracellular matrix. Cells were then seeded in the upper chamber with serum-free medium and incubated for 24 h. Invading cells in the lower chamber were fixed, stained with crystal violet, and counted under a light microscope.

### Intracellular total RNA Extraction and Quantitative RT-PCR (qRT-PCR)

Total RNA was extracted from cultured cell lines using the RNeasy Kit (QIAGEN, Germantown, MD, USA) following the manufacturer’s protocol. Two micrograms of total RNA were reverse transcribed using M-MLV Reverse Transcriptase (Thermo Fisher Scientific) and RNaseOUT™ Recombinant Ribonuclease Inhibitor (Thermo Fisher Scientific). qRT-PCR was performed using SYBR Green 2 qPCR Master Mix (SMO-bio, Seoul, Korea) with primers listed in Supplementary Table S4. Comparative gene expression analysis was performed using the 2 ^−ΔΔCt^ method with data normalized to the level of human GAPDH, which was used as an internal control. These normalized values were represented as relative gene expressions.

### Nuclear and Cytoplasmic Cell Fraction

Fractions were collected manually to investigate the relative distribution of MSN or STAT3/p-STAT3 in the cytosol and nucleus, depending on MSN expression. Briefly, cells were harvested and washed using 0.9% sodium chloride solution. Cells were then resuspended and homogenized in a lysis buffer containing 0.5% NP-40, 10 mM Tris–HCl pH 7.4, 10 mM NaCl, and 3 mM MgCl_2_. A low-speed centrifugation step was employed to pellet the nuclei and any unbroken cells. Subsequently, the cytosolic fraction supernatant was carefully transferred to a fresh tube. The pellet was lysed using protein lysis buffer (Cell signaling) to isolate the nucleus. Cytosolic and nuclear proteins were confirmed with Western blotting. GAPDH (Santa Cruz Biotechnology) and Lamin A/C (Santa Cruz Biotechnology, Cat# sc-376248, RRID:AB_10991536) antibodies were used as an internal control of cytosolic and nuclear proteins, respectively.

### Co-immunoprecipitation (Co-IP) Assay

In a standard Co-IP assay, cells were lysed using a cell lysis buffer (Cell Signaling) supplemented with protease and phosphatase inhibitors (Thermo Fisher Scientific). After centrifugation to remove cell debris, 500 μg of protein was incubated with 1 μg of the primary antibody at 4 °C for 4 h. Subsequently, protein G-agarose beads (GenDEPOT, Sugar Land, TX, USA) were added to the mixture and incubated overnight at 4 °C. The following day, the beads were collected by centrifugation and washed three times with PBS. The clarified beads were then resuspended in an equal volume of 2x SDS sample buffer and boiled at 95 °C for 5 min. The resulting immunoprecipitates were subjected to western blot analysis.

### Tumorsphere Formation Assay

Each MSN-overexpressing or knock-down cell line was cultured in DMEM-GlutaMAX medium (Gibco) supplemented with 1 × B27 (Invitrogen), 20 ng/ml of epidermal growth factor (EGF; Pepro Tech), and 40 ng/ml of essential fibroblast growth factor (bFGF; Pepro Tech) in 96-well ultra-low attachment plates (BD Corning). After a 7-day incubation period, tumorsphere formation was observed using an inverted microscope, and the number of tumorspheres formed (> 100 μm in diameter) was counted. Images were captured for documentation. For secondary tumorsphere-formation assays, primary tumorspheres (> 100 μm) were harvested after seven days and suspended with 0.05% trypsin EDTA to dissociate into single cells, which were subsequently cultured as described.

### Flow cytometry analysis

For analysis of the breast cancer stem cell (CSC) population, cells were stained with allophycocyanin (APC)-conjugated CD44 (BD Biosciences, Cat# 559,942, RRID:AB_398683) and phycoerythrin (PE)-conjugated CD24 (BD Biosciences, Cat# 559,942, RRID:AB_398683) antibodies at room temperature for 20 min. The subpopulation of CD44^+^/CD24^−^ cells was measured using FACSymphony A3 (BD Biosciences). For MSN knock-down cell lines, transient MSN knock-down cells using siRNA were utilized for flow cytometry analysis due to GFP interference of lentiviral MSN shRNA. To evaluate cell population with a high ALDH enzymatic activity, ALDEFLUOR assays were performed following the manufacturer’s instructions provided with the kit (Merck, Rahway, NJ, USA). In brief, 5 × 10^5^ cells were incubated in 500 μl of ALDEFLUOR assay buffer containing 0.6 μg ALDH substrate and incubated at 37 °C for 30 min. Cells treated with diethylaminobenzaldehyde (DEAB), a specific inhibitor of ALDH, were used as a negative control for each experimental group. The sorting gates were established based on cell viability and the negative controls (ALDEFLUOR-stained cells treated with DEAB), and the ALDEFLUOR-positive population was measured using FACSymphony A3 (BD Biosciences). Data were analyzed using Flowjo 10.8.1 software (BD Biosciences, RRID:SCR_008520). To analyze apoptotic cells, staining with APC-labelled annexin V and propidium iodide (PI) was performed using the Annexin V Apoptosis Detection Kit (Sigma-Aldrich). Annexin V -APC and PI were diluted at ratios of 1:200 and 1:100, respectively, and incubated at room temperature for 20 min.

### Confocal microscopic analysis

Confocal microscopy was used to examine the localization of pSTAT3 and MSN under IL-6 treatment conditions. Cells were seeded in confocal dishes (SPL Life Science, Seoul, Korea) and cultured until they reached 60–70% confluency. The cells were then fixed with 4% paraformaldehyde (PFA) for 15 min at room temperature, permeabilized using 0.1% Triton X-100 (Sigma-Aldrich) for 10 min, and blocked with 10% goat serum (Invitrogen) for 1 h to minimize non-specific binding. Primary antibodies against pSTAT3 (Cell Signaling, Cat# 9145, RRID:AB_2491009) and MSN (Santa Cruz Biotechnology) were diluted at 1:500 in 1% BSA and incubated overnight at 4 °C. After washing thoroughly with PBS, cells were incubated with secondary antibodies conjugated to Alexa Fluor 594 (red) or FITC (green) at a 1:500 dilution for 1 h at room temperature. Cell nuclei were stained using DAPI (blue) to visualize nuclear localization. To quantify nuclear co-localization of pSTAT3 and MSN, signal intensities were measured using ImageJ by analyzing the overlap with DAPI staining. The average nuclear intensity of the overlapping signals was calculated and compared between groups. For LPAR1 and IL-6 co-localization studies, MDA-MB-231 cells were treated with 50 ng/ml FITC-conjugated IL-6 (green) for 15 min, followed by incubation with anti-LPAR1 antibody (Santa Cruz Biotechnology, Cat# sc-130361) at 4 °C overnight. After washing, cells were treated with rhodamine-conjugated secondary antibody (red) for 1 h to detect LPAR1 expression. DAPI was used for nuclear staining. Images were captured using a Leica STED CW fluorescence microscope (Leica Biosystems) under identical exposure conditions across samples. Co-localization analysis was performed using the Leica Application Suite X software (LASX, version 1.4.6), and any post-capture image processing was standardized to maintain consistency across experimental conditions.

### Enzyme-linked Immunosorbent Assay (ELISA)

To confirm the functional protein levels of IL-6 in cell culture supernatants, the medium was collected from adherent MSN-shRNA and MSN-overexpressing transduced TNBC cells. The medium from each cell line was concentrated using an ultra-centrifuge filter (Gibco). The IL-6 levels were determined using the IL-6 DuoSet ELISA Development System (R&D Systems, Minneapolis, Minnesota, USA) with specific antibodies against human IL-6, following the manufacturer’s instructions.

### Dual Luciferase Promoter Assay

To measure IL-6 promoter activity based on MSN expression, the Dual-Luciferase Reporter Assay System was used (Promega). Briefly, after cells were seeded and allowed to adhere for one day, they were transfected with the control firefly reporter plasmid pGL4.54 (Promega) and co-transfected with either the luciferase reporter plasmid pLN1.21 (Promega) or pLN1.21 containing the IL-6 promoter region (Accession no. OL961307, nt 1- nt 229) including NF-κB motifs (AATGTGGGATTTTCCCATG) and STAT3 binding motifs (CCGGGAA). After 48 h of transfection, luciferase activity was measured using the Nano-Glo® Dual-Luciferase® Reporter Assay System (Promega) as described in the manufacturer’s instructions.

### qPCR Array for GPCR-related and Cytoskeleton-related Genes

RT-qPCR was performed using two Accutarget™ qPCR Screening Kits (BIONEER) for target gene screening. The first kit contains gene-specific primers for 84 genes related to GPCR, and the second kit includes gene-specific primers for 84 genes associated with Rho family genes and cytoskeleton regulators. PCR amplification was carried out using SYBR Green PCR Master Mix (SMO-bio) according to the manufacturer’s instructions. Target gene expression was normalized to endogenous GAPDH using the comparative cycle threshold method.

### Chromatin Immunoprecipitation (ChIP) Assay

The ChIP assay was performed using a Chromatin Immunoprecipitation Assay Kit (Merck). Briefly, cells were crosslinked with 1% formaldehyde (Sigma-Aldrich) and lysed with nucleus lysis buffer containing phenylmethylsulfonyl fluoride (PMSF; Sigma-Aldrich) for 20 min on ice. The lysate was then sonicated to obtain sheared DNA fragments of approximately 200–1000 bp. Subsequently, the chromatin was incubated and precipitated with antibodies against STAT3 (Cell Signaling, Cat# 9139, RRID:AB_331757) or IgG (Santa Cruz Biotechnology, Cat# sc-2025). The DNA–protein immunocomplexes were collected using protein A-agarose beads, and the DNA was purified. Finally, the target gene was detected using the purified DNA as a template for conventional PCR. The PCR product was electrophoresed on a 2% agarose gel. c-Fos served as a positive control since STAT3 has been shown by ChIP assay to bind and up-regulate c-Fos expression. Primers for detecting the target genes are listed in Supplementary Table S5.

### RNA Sequencing (RNA-seq) and Data Analysis

To construct cDNA libraries, 100 ng of total RNA was used with the CORALL RNA-Seq Library Prep Kit (Lexogen, Inc., Greenland, NH, USA). This protocol involved polyA-selected RNA extraction, RNA fragmentation, random hexamer-primed reverse transcription, and purification of library fragments using the AMPure XP system (Beckman Coulter, Beverly, USA). PCR amplification was conducted with Phusion High-Fidelity DNA polymerase, Universal PCR primers, and Index (X) Primer. The PCR products were subsequently purified using the AMPure XP system, and the quality of the library was assessed using the Agilent 2100 Bioanalyzer Instrument (Agilent Technologies, Santa Clara, CA, USA, RRID:SCR_018043). Clustering of the index-coded samples was performed on a cBot Cluster Generation System using TruSeq PE Cluster Kit v3-cBot-HS (Illumina, Inc., San Diego, CA, USA), according to the manufacturer’s instructions. The libraries were sequenced on an Illumina Novaseq platform, generating 150 bp paired-end reads. For quality control, raw FASTQ data were processed using Novogene Perl scripts to obtain clean reads by removing adapters sequences, poly-N sequences, and low-quality reads while calculating Q20, Q30, and GC content. Clean reads were mapped to the reference genome using HISAT2 (RRID:SCR_015530), which includes splice junctions from gene model annotations to improve alignment accuracy. Gene expression levels were quantified using featureCounts (RRID:SCR_012919) and expressed as FPKM, accounting for gene length and sequencing depth. Normalization and differential expression analyses were conducted using the DESeq2 (RRID:SCR_015687) package in R software 4.2.3. For conditions with biological replicates, hierarchical clustering was performed using a negative binomial model, with p-values adjusted using the Benjamini–Hochberg method. Genes with adjusted *p*-values ≤ 0.05 and absolute │ Log2(1.2) │ were considered significantly differentially expressed. Hierarchical clustering was based on Pearson’s correlation coefficient. Gene Ontology (GO) analysis and the Coexpression feature were utilized on the ToppGene site (https://toppgene.cchmc.org/, accessed on March 29, 2024) to analyze the input gene data. Raw RNA-Seq data have been uploaded to the Gene Expression Omnibus (GEO) database (https://www.ncbi.nlm.nih.gov/geo) at the National Center for Biotechnology Information (NCBI) (GEO no. GSE272103 and GSE272101).

### Public Data

Publicly available datasets from human breast cancer patients, including METABRIC, The Cancer Genome Atlas (TCGA), and the Cancer Cell Line Encyclopedia (CCLE), were obtained from cBioPortal (http://www.cbioportal.org/) and re-analyzed. In addition, the GSE dataset was downloaded from the Gene Expression Omnibus (GEO) (RRID:SCR_005012) database for further analysis. Disease-free survival (DFS) and overall survival (OS) of breast cancer patients in METABRIC, TCGA, and GSE datasets were analyzed using Cancer Target Gene Screening (CTGS; http://ctgs.biohackers.net). To compare CSC biomarkers selected in this study, the Biomarkers of Cancer Stem Cells database (BCSCdb; a database of biomarkers of cancer stem cells [17]) was used. Forest plots representing the hazard ratio (HR) and 95% confidential intervals (CIs) of selected genes in a Cox proportional hazard regression model were analyzed.

### Statistical analysis

All statistical analyses were performed using the SPSS version 26.0 software (IBM Corp., Armonk, NY, USA, RRID:SCR_002865) or GraphPad Prism version 10.3.1 (GraphPad Software). The chi-square was utilized to investigate the potential association of the expression of MSN in TNBC patients with various clinicopathological parameters. For correlation analysis, the Pearson correlation coefficient was used to calculate the correlation between two factors of interest. For DFS and OS survival analyses, the Kaplan–Meier curves were used, and different groups were compared using a log-rank test. The statistical significance of the differences between the means of the two groups was analyzed using an unpaired Student’s *t*-test. For multiple group comparisons, a one-way ANOVA with post-hoc Tukey’s test was used. Experiments were repeated at least three times and expressed as mean ± SD. All *P*-values were two-sided. *P*-value less than 0.05 was considered statistically significant.

## Results

### MSN is Associated with Poor Clinical Outcomes in TNBC

**Fig. 1:**
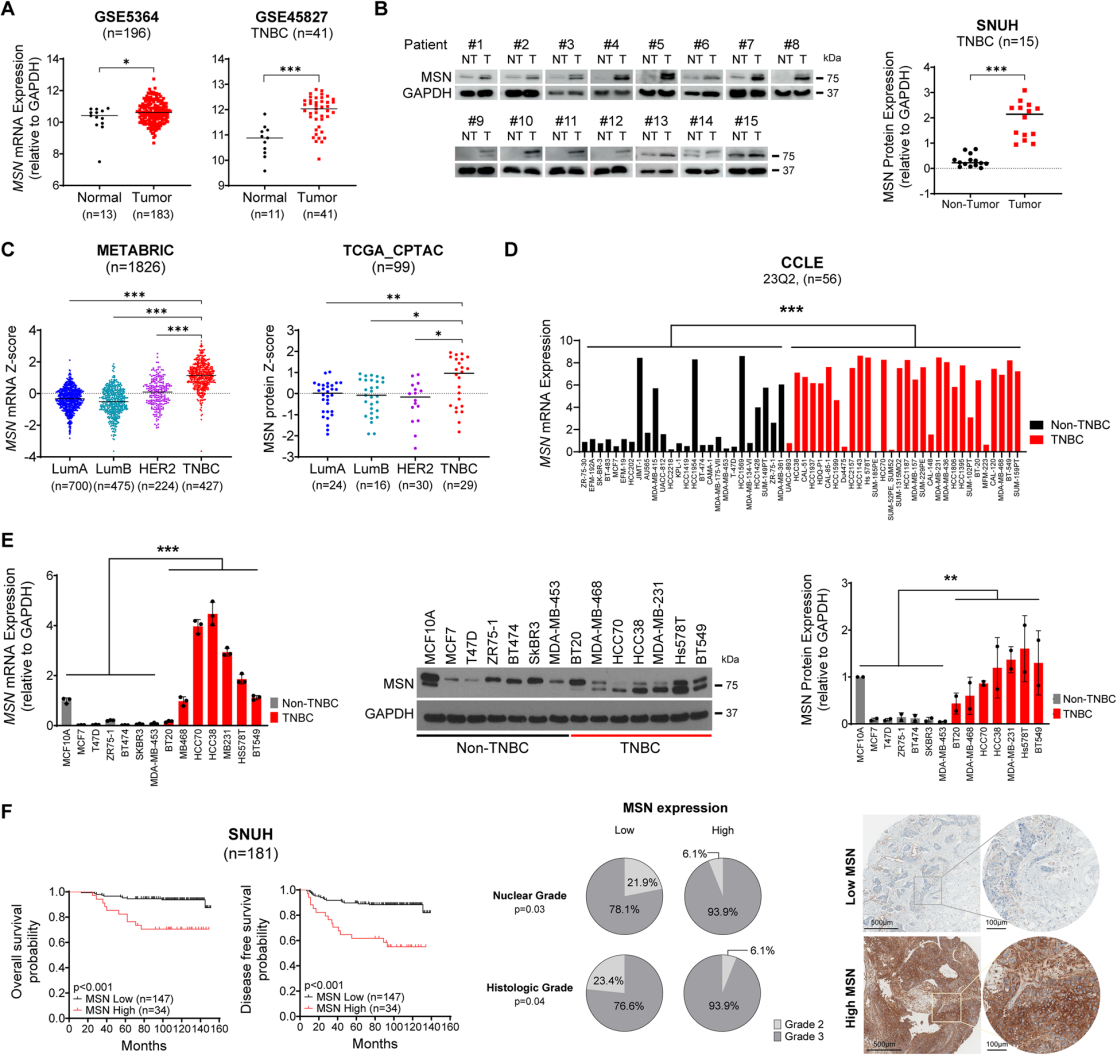
Elevated MSN Expression Predicts Poor Prognosis in TNBC Patients.** A** MSN mRNA expression levels in normal breast and breast cancer tissues from the GSE5364 dataset (left) and in normal versus TNBC tissues from GSE45827 dataset (right).** B** Western blot analysis of MSN protein levels in TNBC tissues and paired adjacent non-tumor tissues from the SHNU cohort (left). The normalization of the quantification to GAPDH is shown (right).** C** MSN mRNA expression levels across breast cancer subtypes from the METABRIC dataset (left) and protein expression from the TCGA_CPTAC dataset (right). **D** MSN mRNA expression levels between non-TNBC and TNBC cell lines, based on CCLE 23Q2.** E** MSN mRNA expression (left; performed in triplicate) and protein expression (middle and right; performed in duplicate) in non-TNBC and TNBC cell lines, normalized to GAPDH. **F** Kaplan–Meier survival analysis of TNBC patients from the SNUH cohort (*n* = 181), stratified into high- and low-MSN expression groups for overall survival (upper left) and disease-free survival (upper right), using an H score of 200 as the cut-off. Pearson Chi-square analysis evaluated between MSN expression and histologic grade or nuclear grade. Representative IHC staining images from breast cancer sections are shown (right). Scale bars: 500 μm (low power) and 100 μm (high power). Data are presented as the median for panels A, B, and C, while the remaining data are expressed as mean ± SD. Statistical significance was determined using Student’s *t*-test (A, B, D, E), ANOVA followed by the Tukey test (C), and the Log-rank test or Chi-Square test (F). *P < 0.05, **P < 0.01, ***P < 0.001

Our previous study has supported the oncogenic functions of MSN in human bladder cancer [10]. To further define the biological roles of MSN in breast cancer, MSN mRNA and protein expression levels were investigated in multiple breast cancer cohorts. The GEO microarray datasets (GSE5364 and GSE45827) revealed a significant upregulation of MSN transcript in all intrinsic subtypes of breast cancer, including TNBC, compared to normal tissue (Fig. 1A, *p* = 0.0335; all subtypes and *p* < 0.0001; TNBC, respectively). Western blotting of our cohorts (*n *= 15) also confirmed that there were approximately 6.5 higher levels of MSN protein expression in TNBC tissues compared to paired adjacent non-tumor breast parenchyma (Fig. 1B, *p* < 0.001). Notably, the comparative analysis for all intrinsic subtypes demonstrated that TNBC exhibited the highest levels of MSN mRNA and protein expression in METABRIC and TCGA datasets (*n* = 598, Fig. 1C and Supplementary Fig.  1 A). Consistent with the human breast cancer cohorts, MSN mRNA and protein expression was significantly elevated in TNBC cell lines compared to non-TNBC breast cancer cell lines in the comparative analysis with the Cancer Cell Line Encyclopedia (CCLE) dataset (Fig. 1D, *p* < 0.001), and our breast cancer cell line library (Fig. 1E, *p *< 0.001; mRNA and *p* = 0.002; protein, respectively). These findings collectively indicate that MSN is highly expressed in breast cancer tumor tissues, particularly within the TNBC subtype. In the METABRIC and in-house patient cohorts, TNBC patients with higher MSN expression had lower overall survival (OS) rates (*p* = 0.048 and *p* < 0.001, respectively). A significant difference in disease-free survival (DFS) was also observed in the SNUH and KM plotter datasets (*p* < 0.001 and *p* = 0.061, respectively). Further analysis revealed that higher protein expression of MSN was associated with advanced histologic grade (*p* = 0.04) and nuclear grade (*p* = 0.03) (Fig. 1F, Supplementary Fig. 1B, and Supplementary Table S6). Taken together, these findings revealed the clinical evidence that tumors with elevated MSN expression are significantly associated with worse clinical outcomes, supporting the notion that MSN may potentiate the aggressiveness of TNBC.

### MSN Enhances Tumor Growth and Distant Organ Metastasis in TNBC

**Fig. 2:**
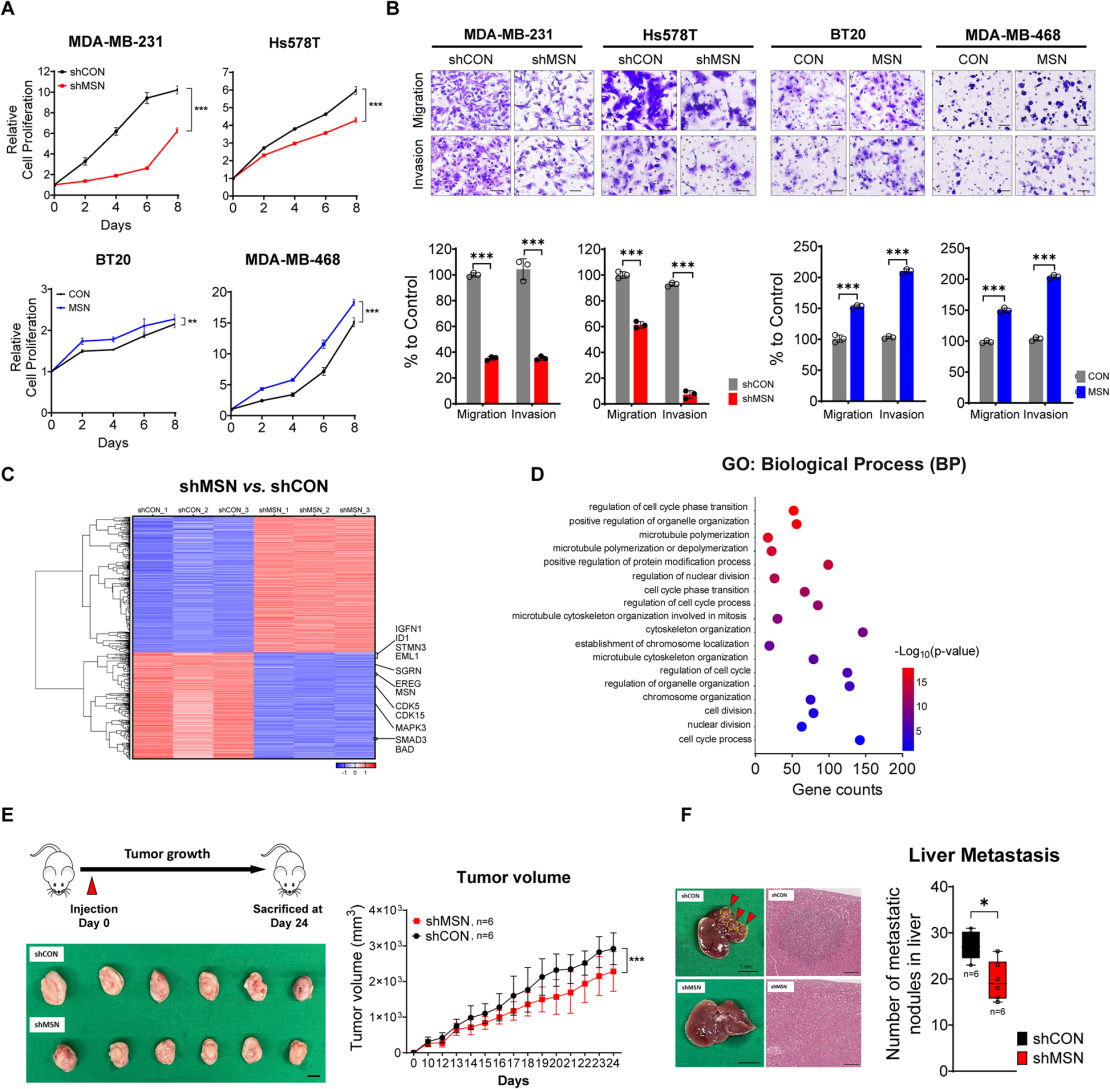
MSN Drives Tumor Progression and Increases Metastatic Spread in TNBC.** A** Proliferation measured using Glo Titer Assay in shMSN or MSN-overexpressing cell lines compared to their respective control cell lines. Experiments were performed in three independent biological replicates (*n* = 3). **B** Transwell migration and invasion assays performed with Matrigel (upper: images; lower: quantification). Assays were conducted in three independent biological replicates (*n* = 3). **C** Heatmap from RNA-seq analysis showing differentially expressed genes between MDA-MB-231/shMSN and MDA-MB-231/shCON, with hierarchical clustering (adjusted *p* ≤ 0.05, │Log2(1.2)│).** D** GO enrichment analysis of differentially expressed genes (FDR < 0.05). The dot color indicates -log10 (*p*-value); the x-axis represents gene counts, and the y-axis shows GO terms. **E** Schematic of the animal study (top left). Mice injected with 4T1/shCON or 4T1/shMSN cells were monitored for 24 days. Tumor volumes and tumor weights (boxplot) were compared (*n* = 6 per group). **F** Representative liver images showing metastasis (gross appearance and H&E staining). Scale bar: 100 µm. A Boxplot comparing the number of liver micrometastatic nodules is shown (*n* = 6 per group). All data are represented as mean ± SD. Statistical significance was determined by mixed regression (A, E) and Student’s *t*-test (B, F). *P < 0.05, **P < 0.01, ***P < 0.001

To validate the independent effect of MSN on biological features without being influenced by other Ezrin-Moesin-Radixin (ERM) subfamily members [18], such as Ezrin (EZR) and Radixin (RDX), we first performed knockdown experiments targeting MSN (Supplementary Fig.  2 A). The results demonstrated that knocking down MSN did not affect the expression of EZR and RDX. Similarly, suppression of EZR or RDX did not influence the expression of MSN. Following this confirmation, we investigated the role of MSN in breast cancer by establishing cell lines with altered MSN expression in TNBC (Supplementary Fig. 2B). When analyzing the impact of MSN expression on cell proliferation, we found that knockdown of MSN inhibited cell proliferation, whereas overexpression of MSN rescued cell proliferation (Fig. 2A). Additionally, MSN expression significantly enhanced cell migration and invasion abilities of tumor cells (Fig. 2B). To further investigate gene expression changes and the underlying molecular mechanisms associated with MSN expression, we subjected MDA-MB-231/shMSN cells and their control cells (MDA-MB-231/shCON) to RNA sequencing (RNA-seq). A total of 1,301 genes were significantly downregulated upon MSN depletion (Fig. 2C, Supplementary Fig.  2 C, and Supplementary Table S7). Differentially expressed genes (DEGs) profiles between the two groups were statistically enriched for gene sets linked to kinase binding, the connection between a cell and the extracellular matrix, cell cycle process, and microtubule cytoskeleton organization in mitosis (Fig. 2D and Supplementary Table S8). This finding aligns with the observed aggressive tumor behavior associated with high MSN expression, suggesting that MSN may play a crucial role in promoting these oncogenic processes. Consistent with the in vitro and transcriptomic results, the orthotopic xenograft mouse models harboring MSN-knockdown tumors with the established 4T1/shMSN cell lines showed impaired tumor growth compared to the control mice (Supplementary Fig. 2D and Fig. 2E). Additionally, MSN knockdown was observed to reduce both microscopic metastatic lesions and macroscopic metastatic nodules in the liver compared to the control group (Fig. 2F). Collectively, these results suggest that MSN promotes tumor growth and metastasis in TNBC.

### Activated MSN by IL-6 Involves Autoloop of IL-6 Transcription and NF-κB Phosphorylation

**Fig. 3:**
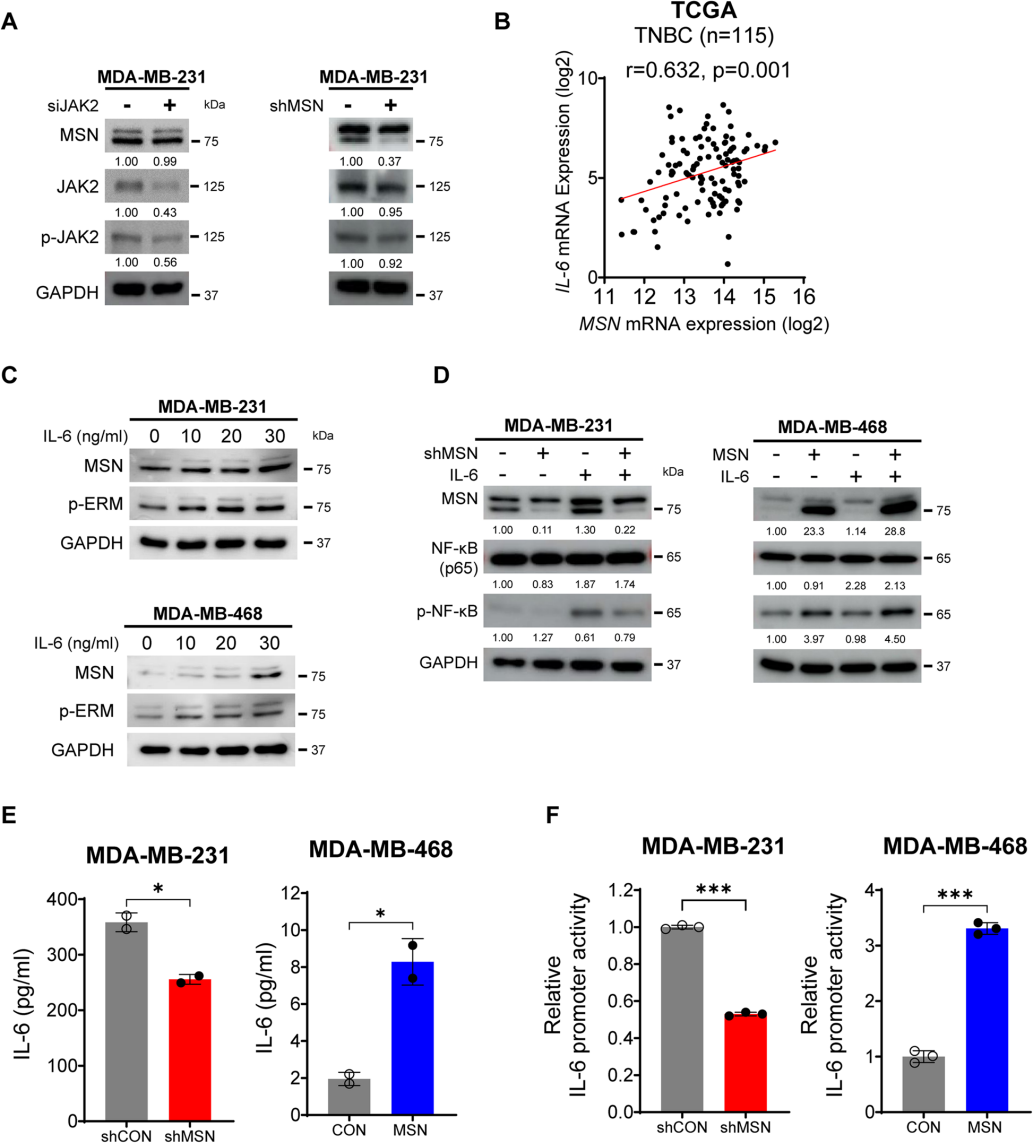
IL-6-Induced Activation of MSN Drives an Autoregulatory Loop of IL-6 Transcription and NF-κB Phosphorylation. **A** Western blot analysis showing JAK2 knockdown on MSN, JAK2, and p-JAK2 expression in MDA-MB-231 cells. Comparison between MDA-MB-231/shMSN and MDA-MB-231/shCON cells. GAPDH was used as an internal loading control. **B** Correlation analysis of IL-6 and MSN mRNA expression levels in TNBC patients, based on the TCGA dataset. **C** Western blot analysis of MSN and p-ERM expression in MDA-MB-231 and MDA-MB-468 cells treated with increasing doses of IL-6. **D** Western blot analysis of MSN, NF-κB, and p-NF-κB in MDA-MB-231/shMSN and MDA-MB-468/MSN cell lines following IL-6 treatment. **E** Measurement of secreted IL-6 levels by ELISA in MDA-MB-231/shMSN (*vs.* MDA-MB-231/shCON) and MDA-MB-468/MSN (*vs.* MDA-MB-468/CON) cells. Experiments were performed in duplicate (*n* = 2). **F** Relative IL-6 promoter activity measured by Dual-Luciferase Reporter Assay in MDA-MB-231/shMSN and MDA-MB-468/MSN cell lines. Experiments were performed in triplicate (*n* = 3). All data are shown as mean ± SD. Statistical significance was determined by Student’s *t*-test (E, F). *P < 0.05, ***P < 0.001

As MSN contains a FERM domain identically found in JAK2 [11] and IL-6 stimulates hyperactivation of JAK signaling [19], we next examined whether IL-6 acts on tumor cells to activate MSN. Due to their protein structural similarity, we initially explored the crosstalk between JAK2 and MSN to exclude JAK2 interference. Inhibition of JAK2 did not affect MSN expression, nor did suppressing MSN significantly impact JAK2 expression, indicating independent operation of these pathways (Fig. 3A). Therefore, we further examined the effect of IL-6 on MSN expression and phosphorylation, as JAK is controlled by IL-6 [20]. The increased MSN expression was correlated with IL-6 mRNA levels in TNBC patients (Fig. 3B, *n*= 115, *r* = 0.632, *p* = 0.001; TCGA). Consistently, IL-6 treatment increased MSN expression and promoted MSN phosphorylation (Fig. 3C and Supplementary Fig.  3 A). Since IL-6 is a key cytokine that activates the NF-κB pathway within the cytoplasm and NF-κB is well-described as a crucial transcription factor for various types of chemokines as well [21], we assumed that MSN might involve this circular loop of IL-6 transcription by NF-κB phosphorylation and IL-6-induced NF-κB activation. Interestingly, upon MSN upregulation in tumor cells, there was an increase in phosphorylated NF-κB (p-NF-κB) under IL-6 treatment. In contrast, MSN knockdown in TNBC cells resulted in a reduction of p-NF-κB. However, the total NF-κB expression remained unchanged by MSN modulation or presence of IL-6 (Fig. 3D and Supplementary Fig. 3B). Concurrently, ELISA analysis indicated that tumor cells with higher MSN expression exhibited increased levels of secreted IL-6, while those with low MSN expression had reduced levels (Fig. 3E and Supplementary Fig.  3 C). A promoter assay also demonstrated that IL-6 promoter activity was highly enhanced in the group of MSN overexpression compared to the tumor cells with MSN depletion (Fig. 3F and Supplementary Fig. 3D). Collectively, these findings imply that IL-6-induced MSN elevates p-NF-κB levels, which in turn amplifies IL-6 production, establishing an autoregulatory feedback loop in TNBC.

### IL-6 binding lysophosphatidic acid receptor 1 (LPAR1) activates MSN mediated CDC42-PAK4 Signaling Axis and NF-κB Phosphorylation

**Fig. 4:**
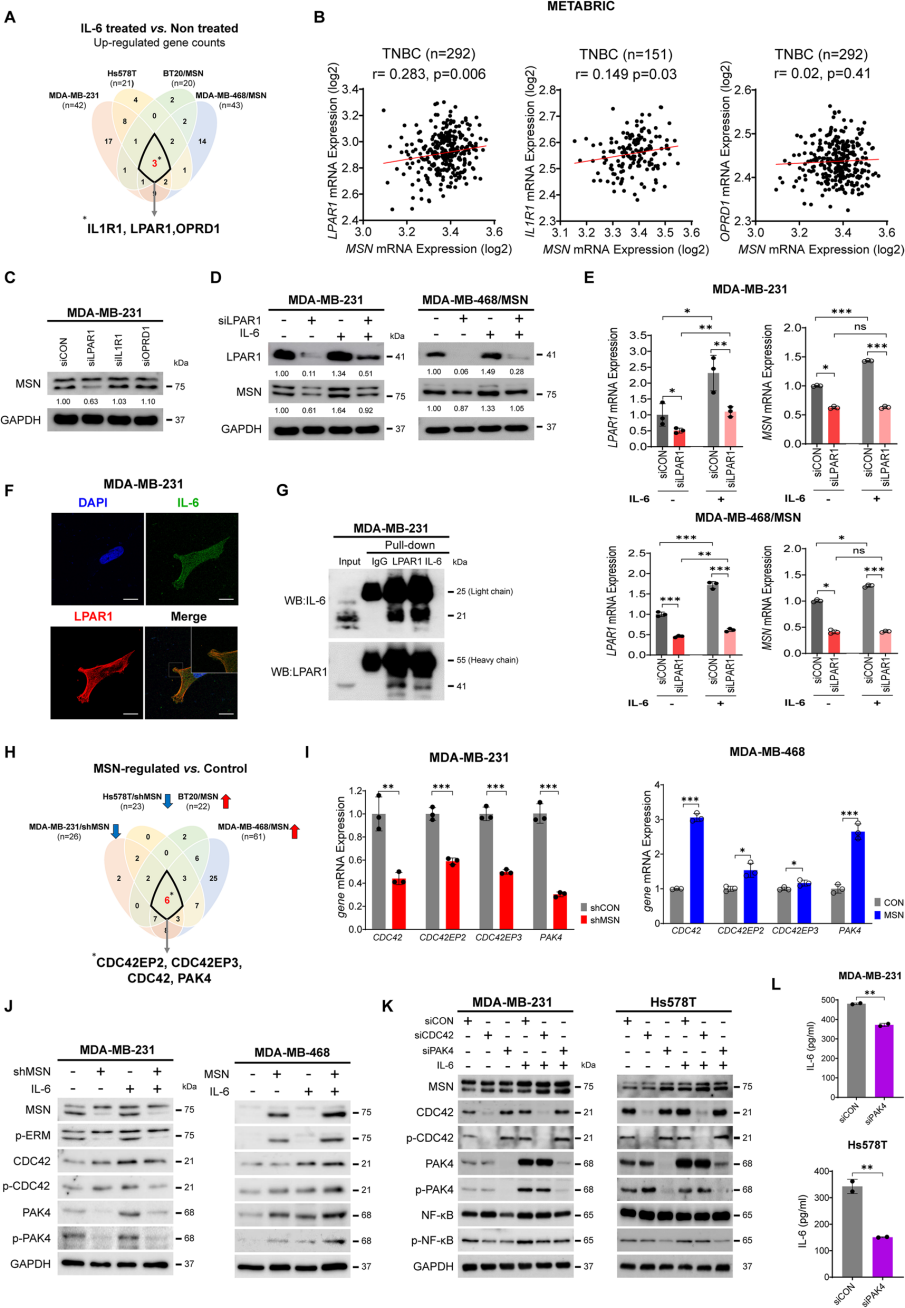
IL-6 Binding to LPAR1 Triggers Activation of the MSN-Mediated CDC42-PAK4 Signaling Pathway and Promotes NF-κB Phosphorylation.** A** Venn diagram showing common upregulated GPCR-related genes identified via qPCR panel following IL-6 treatment in MSN-expressing cell lines. **B** Correlation analysis of MSN with LPAR1, IL1R1, and OPRD1 mRNA expression levels in TNBC patients from the METABRIC dataset. **C** Western blot analysis of MSN expression in MDA-MB-231 cells transfected with siRNAs targeting LPAR1, IL1R1, and OPRD1, normalized to GAPDH. **D** Western blot analysis of LPAR1 and MSN expression in MDA-MB-231 and MDA-MB-468/MSN cells after LPAR1 knockdown, with or without IL-6 treatment. **E** Relative mRNA expression of LPAR1 and MSN in MDA-MB-231 and MDA-MB-468/MSN cells after LPAR1 knockdown, with or without IL-6 treatment. Experiments were performed in triplicate (*n* = 3). **F** Confocal microscopy images of MDA-MB-231 cells treated with FITC-IL-6 and anti-LPAR1, showing co-localization. Scale bar: 10 μm. **G** Immunoprecipitation of IL-6 and LPAR1 in MDA-MB-231 cells, confirmed by Western blot using anti-LPAR1, anti-IL-6, and anti-IgG. **H** Venn diagram showing upregulated or downregulated Rho GTPase-related genes in MSN-overexpressing and knockdown cells. Six common genes were selected, excluding MSN and EZR. **I** Relative mRNA expression of selected targets in shMSN or MSN-overexpressing cell lines compared to control cell lines, measured via RT-qPCR. Experiments were performed in triplicate (*n* = 3). **J** Western blot analysis of target proteins in shMSN or MSN-overexpressing cell lines, with or without IL-6 treatment, normalized to GAPDH. **K** Western blot analysis of target proteins in MDA-MB-231 and Hs578T cells transfected with siRNAs for CDC42, PAK4, or siCON, with or without IL-6 treatment. GAPDH was used as loading control. **L** Secreted IL-6 levels in the supernatant of MDA-MB-231 and Hs578T cells transfected with siPAK4 or siCON, measured by ELISA. Experiments were performed in duplicate (*n* = 2). All data are represented as mean ± SD and statistical significance was determined using Student’s *t*-test (E, I, L). *P < 0.05, **P < 0.01, ***P < 0.001; ns, not significant

To elucidate which receptor is the target for IL-6 biding and regulates MSN expression and phosphorylation in TNBC, we first focused on IL6R as an IL-6 binding receptor for MSN-mediated signal transduction due to the canonical interaction between IL-6 and IL6R, which is well known from the previous studies [22]. MDA-MB-231 cells with knockdown of IL6R had no alteration in MSN expression, while they reduced JAK2 expression (Supplementary Fig.  4 A). Simultaneous IL-6 administration still induced strong MSN expression regardless of the presence of IL6R (Supplementary Fig. 4B). These findings indicate that IL-6-induced MSN expression operates independently of the IL-6/IL6R interaction, which led us to explore other chemokine binding receptors for IL-6 in MSN mediated intracellular signal transduction. Since chemokine receptors are a family of GPCR that chemokine binds to and activates cellular response [23, 24], we employed a qPCR panel of multiple GPCR-related genes to determine the MSN-related GPCR responses to IL-6 in TNBC (Supplementary Fig.  4 C and Supplementary Table S9). As a result, the mRNA expression levels of IL1R1, LPAR1, and OPRD1 were concurrently altered across all four cell lines upon IL-6 stimulation (Fig. 4A). Further correlation analysis with METABRIC showed a positive correlation of LPAR1 and IL1R1 with MSN mRNA expression (Fig. 4B; *r* = 0.283, *p* = 0.006; LPAR1 and *r* = 0.149, *p* = 0.03; IL1R1, respectively). Transient knockdown of the three genes revealed that only MSN protein levels decreased when LPAR1 expression was inhibited (Supplementary Fig. 4D and Fig. 4C). Additionally, LPAR1 and MSN expression increased following IL-6 treatment (Supplementary Fig. 4E). Reduced MSN expression was identified in IL-6 stimulated cell lines with LPAR1 inhibition at both protein and mRNA levels (Fig. 4D, E). A confocal microscopy assay showed co-localization of IL-6 and LPAR1 after IL-6 treatment. Immunoprecipitation (IP) assays also confirmed the interaction between IL-6 and LPAR1, indicating that the ligand IL-6 binds to its cellular receptor LPAR1 (Fig. 4F, G). Collectively, our findings suggest that LPAR1 may serve as a specific receptor for IL-6, regulating MSN expression in TNBC cells. Because Rho family GTPases serve as a cytoplasmic signal transducer of GPCR [25] and MSN was regulated by GPCR in our study, we next examined whether MSN regulates Rho family GTPase-related genes in the GPCR triggered downstream signal transduction. A qPCR panel with multiple Rho family GTPase-related genes identified four common genes out of 84, including CDC42, CDC42EP2, CDC42EP3, and PAK4, which were upregulated or downregulated in cell lines upon the MSN expression level (Fig. 4H, Supplementary Fig.  4 F, and Supplementary Table S10). Consistently, qPCR validation confirmed significant alterations of these four genes upon MSN expression, and we finally selected CDC42 and PAK4 as potential candidates based on the combined altered expression level from the qPCR panel and validation analyses (Fig. 4I and Supplementary Fig. 4G). Immunoblot analysis also showed increased CDC42 and PAK4 activity by IL-6 treatment in an MSN-dependent manner (Fig. 4J and Supplementary Fig. 4H). However, transient knockdown of CDC42 or PAK4 exhibited only decreased expression and phosphorylation of NF-κB without alteration of MSN level. PAK4 expression and phosphorylation were restored, and CDC42 phosphorylation was moderately enhanced by exogenous IL-6, repeatedly indicating its critical role in IL-6-induced signaling. This restoration also affected transcription factors NF-κB, essential for IL-6 production (Fig. 4K). PAK4 inhibition significantly reduced IL-6 production in the supernatant (Fig. 4L). In TNBC, LPAR1 might play a unique role in IL-6 binding, sequentially controlling MSN-mediated CDC42-PAK4 Signaling Axis and NF-κB phosphorylation.

### MSN phosphorylates STAT3 and Induces Intranuclear Translocation of p-STAT3 for Transcription of CSC-Related Genes

**Fig. 5:**
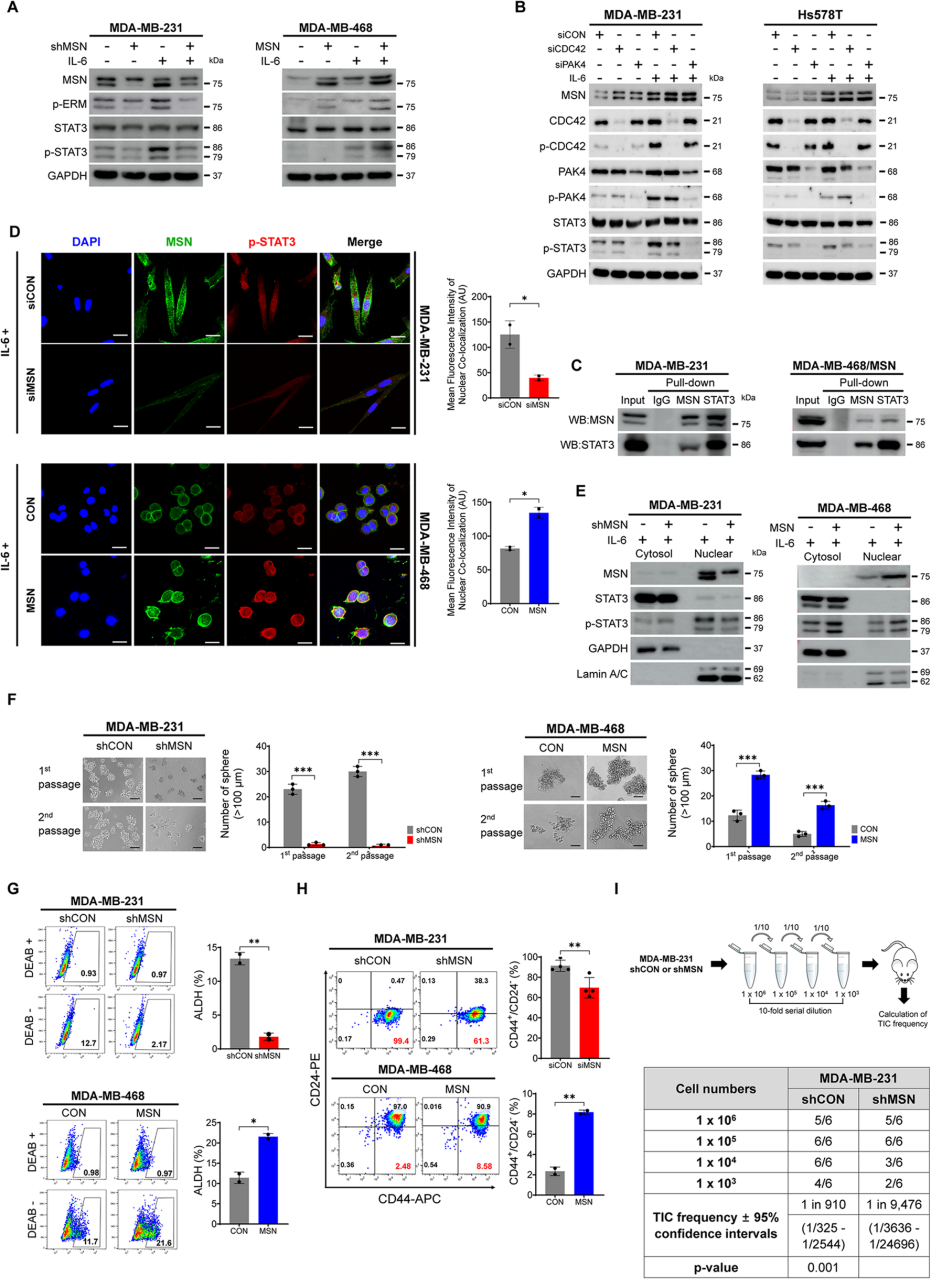

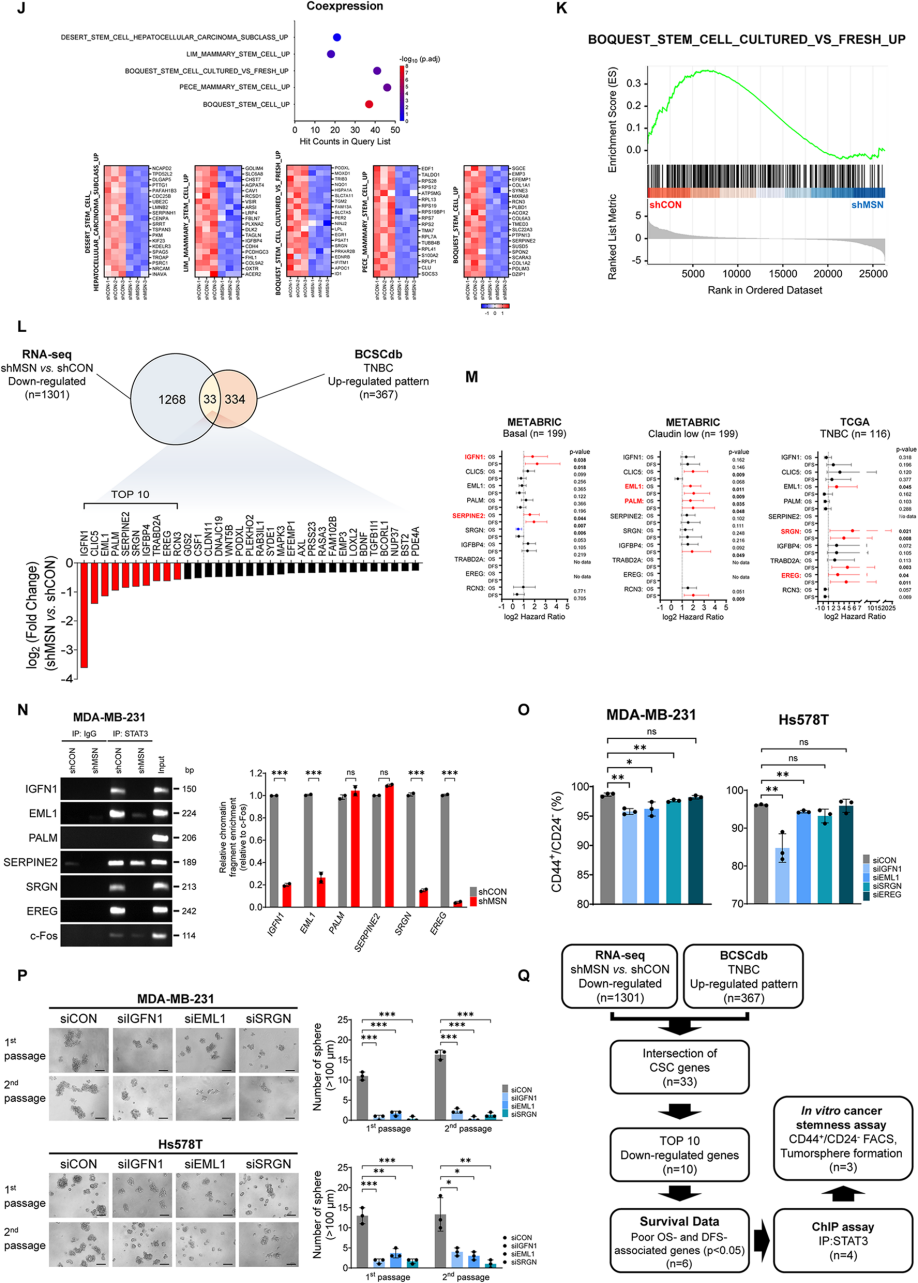
MSN Phosphorylates STAT3, Triggering p-STAT3 Intranuclear Translocation and CSC-Related Gene Transcription.** A** Western blot analysis of STAT3 and related targets in MDA-MB-231/shMSN and MDA-MB-468/MSN cells, with or without IL-6 treatment.** B** Western blot analysis of STAT3 and related targets in MDA-MB-231 and Hs578T cells transfected with siRNAs targeting CDC42, PAK4, or siCON, with or without IL-6 treatment.** C** Immunoprecipitation of MSN and STAT3 in MDA-MB-231 and MDA-MB-468/MSN cells using anti-MSN, anti-STAT3, and anti-IgG, followed by Western blot to confirm binding.** D** Confocal microscopy images of MSN (green), p-STAT3 (red), and DAPI (blue) in MDA-MB-231 cells (siMSN/siCON) and MDA-MB-468/CON/MSN after IL-6 treatment. Nuclear co-localization of p-STAT3 and MSN was quantified using ImageJ by measuring overlap with DAPI staining. Bar graphs represent average nuclear co-localization intensity from two independent experiments. Statistical analysis was performed using *t*-tests. Scale bar: 10 μm.** E** Western blot analysis of cytosolic and nuclear fractions from MDA-MB-231/shMSN and MDA-MB-468/MSN cells for MSN, STAT3, and p-STAT3. GAPDH and Lamin A/C were used as cytosolic and nuclear controls, respectively.** F** Tumorspheres formation assays (day 7) comparing first and second sphere formation. Spheres > 100 μm were quantified. Experiments were performed in duplicate (*n* = 3). **G** and **H** ALDH activity and CD44 ^+^/CD24^ −^ ratio assessed by Flow cytometry in MDA-MB-231/shCON, MDA-MB-231/shMSN, MDA-MB-468/CON, and MDA-MB-468/MSN cells. DEAB-treated cells served as negative controls. ALDH activity (**G**) was measured in duplicate (*n* = 2), and CD44⁺/CD24⁻ ratio (**H**) was assessed in triplicate (*n* = 3). **I** Limiting dilution assay showing TIC frequency in MDA-MB-231/shCON and MDA-MB-231/shMSN cells injected into NOD/SCID mice. TIC frequency was calculated using L-Calc software.** J** Heatmap of the top 20 downregulated genes in RNA-seq data comparing MDA-MB-231/shMSN to controls, analyzed using the COEXPRESSION dataset (ToppGene). **K** GSEA analysis of RNA-seq downregulated gene sets from MDA-MB-231/shMSN compared to controls. Enrichment scores were evaluated using GSEA software. **l** Venn diagram showing overlap between 1,301 downregulated DEGs (MDA-MB-231/shMSN) and 367 upregulated stemness genes (BCSCdb, TNBC), identifying 33 common genes. A waterfall chart highlights log2(fold change) of the 33 genes; the top 10 are marked in red.** M** Forest plots of hazard ratios for the top 10 genes in DFS and OS from METABRIC and TCGA datasets. Genes with p < 0.05 in both analyses are marked in red.** N** ChIP assay evaluating STAT3 binding to promoter/enhancer regions of seven genes, including c-fos as a positive control. Results are shown as fold enrichment relative to c-fos. Experiments were performed in duplicate (*n* = 2). **O** and **P** Flow cytometry analysis of CD44^+^/CD24^−^ populations tumorsphere formation assays (Day 7) in MDA-MB-231 and Hs578T cells transfected with siRNAs targeting the indicated genes. Spheres > 100 μm were quantified. Both assays were performed in triplicate (*n* = 3). **Q** Flowchart summarizing CSC-related gene selection. From 1,301 downregulated DEGs and 367 upregulated stemness genes, 33 common genes were identified. The top 10 genes were narrowed to 3 based on poor prognosis and STAT3 regulation confirmed by ChIP. Sphere formation and CD44^+^/CD24^−^ assays validated the final selection. All data are shown as mean ± SD. Statistical significance was determined by Student’s *t*-test (F, G, H, N O, P). *P < 0.05, **P < 0.01, ***P < 0.001

Since the role of JAK2 to control STAT3 is well known to be initiated by IL-6 stimulation [26] and the structural similarity between JAK2 and MSN [27], we evaluated whether MSN controls STAT3 in TNBC. After the optimal condition for IL-6 treatment for each TNBC cell line (Supplementary Fig.  5 A), we found that IL-6 treatment simultaneously increased the level of phosphorylated MSN and STAT3 without affecting total STAT3 levels (Fig. 5A and Supplementary Fig. 5B). Consistent with the activation of NF-κB, the suppression of CDC42 or PAK4 reduced phosphorylation of STAT3 (Fig. 5B), suggesting that these two specific Rho family GTPases concomitantly regulate the phosphorylation of NF-κB and STAT3 in TNBC. Immunoprecipitation (IP) assays confirmed the interaction between STAT3 and MSN (Fig. 5C and Supplementary Fig.  5 C). Interestingly, confocal microscopy revealed that IL-6 treatment promoted the nuclear translocation of the p-STAT3-MSN complex in an MSN-dependent manner, showing colocalization within the nucleus (Fig. 5D and Supplementary Fig. 5D). However, unlike MSN, the activated form of MSN, p-ERM, did not exhibit significant nuclear translocation upon IL-6 stimulation, as confirmed by imaging analysis. These findings indicate that the primary binding partner of p-STAT3 is the unphosphorylated form of MSN rather than its phosphorylated counterpart, p-ERM, in the context of IL-6-induced MSN expression (Supplementary Fig. 5E). This was corroborated by the analysis of cytosolic and nuclear fractions, which consistently showed the nuclear translocation of p-STAT3 alongside MSN following IL-6 treatment (Fig. 5E and Supplementary Fig.  5 F). Taken together, IL-6-induced MSN expression enhances STAT3 phosphorylation and nuclear translocation of p-STAT3 by direct binding of MSN to p-STAT3. STAT3 is a crucial transcription factor that mediates the expression of various genes associated with cancer stemness [28]. Therefore, we further examined whether the MSN-STAT3 pathway affects cancer stemness in TNBC. First, sphere formation assays and ALDH activity measurements were conducted based on varying levels of MSN expression to prove the connection of MSN to cancer stemness. The sphere formation ability and ALDH activity increased in an MSN-dependent manner (Fig. 5F, G and Supplementary Fig. 5G, H). Additionally, the expression of CSC markers, CD24^−^/CD44^+^, was found in a higher proportion of these cells with elevated MSN expression (Fig. 5H and Supplementary Fig. 5I). Consistent with in vitro results, an in vivo limiting dilution assay showed significantly impaired tumor initiation in the mice group with MSN-knockdown tumors with the established MDA-MB-231/shMSN cell lines, demonstrating that MSN is crucial for enhancing tumor initiation and CSC functions in TNBC (Fig. 5I). The Co-expression dataset available in the ToppGene (https://toppgene.cchmc.org/) from RNA-seq with shRNA-mediated MSN knockdown identified significant downregulation of 142 stem cell-related genes and terms such as stem/progenitor cells and mammary stem cell (Fig. 5J and Supplementary Fig. 5 J, Supplementary Table S11). GSEA analysis further demonstrated that genes upregulated in the cultured stem cells were more actively expressed than fresh stem cells (Fig. 5K). These findings suggest that MSN is critical for maintaining stem cell characteristics in TNBC. To identify candidate genes related to CSC affected by the MSN-p-STAT3 complex, we next performed a stepwise analysis with an in-house dataset and external validation sets. Intersecting the 1,301 DEGs with the 367 upregulated cancer stemness-related genes previously reported in the BCSCdb with TNBC identified 33 common genes modulated by MSN. Among these, the top 10 genes with the lowest expression in MSN-suppressed cells were selected for survival analysis (Fig. 5L and Supplementary Table S12). Using the METABRIC and TCGA datasets, six genes, including IGFN1, EML1, PALM, SERPINE2, SRGN, and EREG, were identified as poor prognostic factors, significantly associated with both OS and DFS (Fig. 5M and Supplementary Fig. 5 K). These six genes were then subjected to IP with anti-STAT3, followed by a ChIP assay for further validation (Fig. 5N). The results indicated that IGFN1, EML1, SRGN, and EREG showed reduced expression in TNBC cell lines with suppressed MSN expression, suggesting that these CSC-related genes are activated by p-STAT3 in conjunction with MSN. Further analysis involved silencing these four genes using siRNA significantly reduced the proportion of CD24^-^/CD44 ^+^ cells for IGFN1, EML1, and SRGN (Supplementary Fig. 5L and Fig. 5O). A sphere formation assay following their suppression showed a significant reduction in sphere formation compared to the control siRNA (Fig. 5P). This series of steps for selecting and validating CSC-related genes is illustrated in a schematic flow chart in Fig. 5Q. These results demonstrate that the p-STAT3 controlled by MSN may induce cancer stemness in TNBC.

### MSN Enhances Chemoresistance to Adriamycin in TNBC

**Fig. 6:**
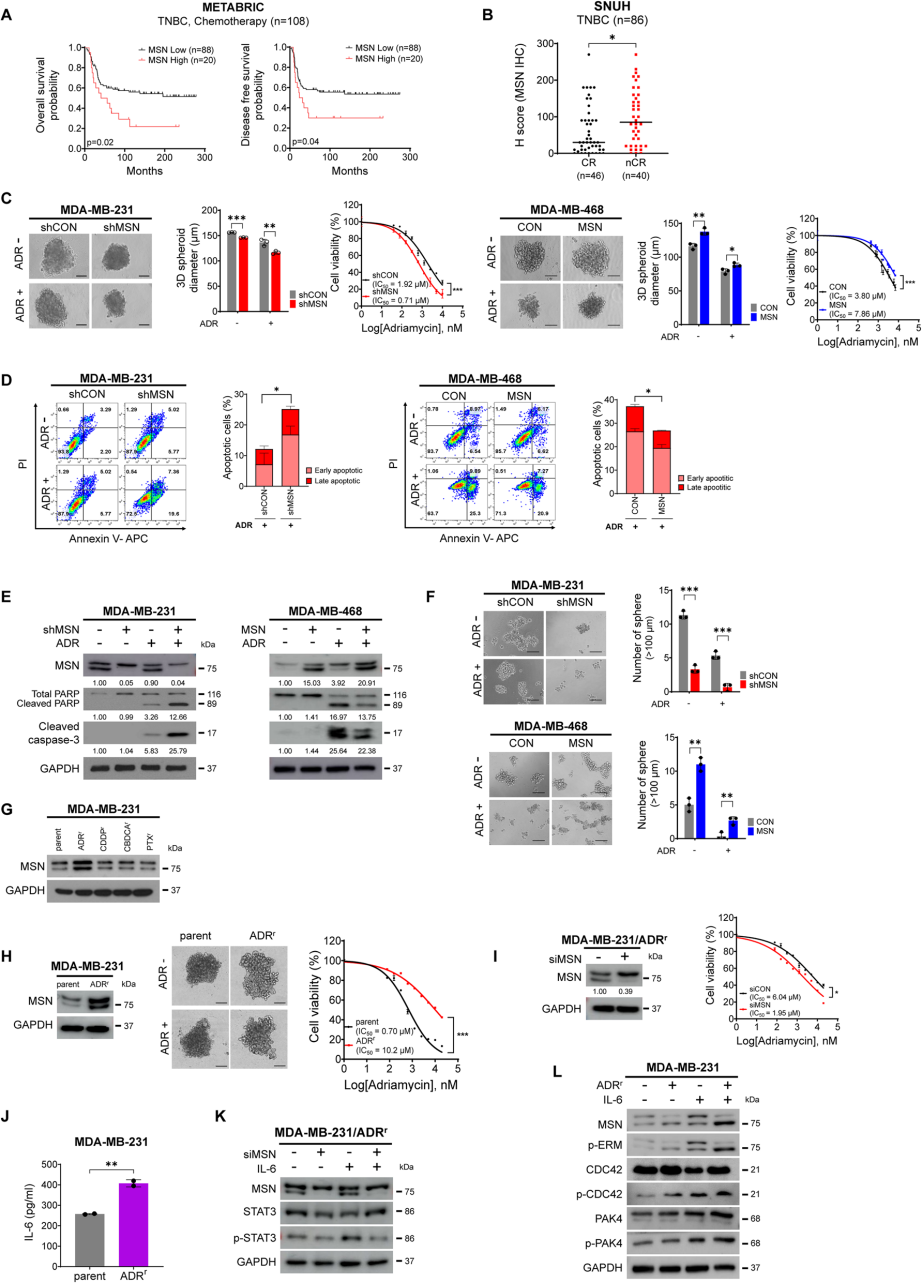
MSN Promotes Chemoresistance to Adriamycin in TNBC.** A** Kaplan–Meier survival analysis of TNBC patients receiving chemotherapy (METABRIC dataset), stratified by high- and low-MSN expression for overall (left) and disease-free survival (right). **B** Immunohistochemistry (IHC) analysis of MSN expression (H-score) in TNBC patients (SNUH cohort) receiving chemotherapy, categorized into complete remission (CR) and non-complete remission (nCR). **C** 3D spheroid culture of MDA-MB-231/shMSN, MDA-MB-468/MSN, and control cells, treated with or without Adriamycin (ADR). Spheroid diameters were measured, and IC50 values were calculated from cell viability after ADR treatment. Experiments were performed in triplicate (*n* = 3). **D** Apoptosis analysis of shMSN and MSN-overexpressing cells with or without ADR, using Annexin V and PI staining. Percentages of apoptotic cells were reported. Experiments were performed in duplicate (*n* = 2). **E** Western blot analysis of the indicated targets in shMSN and MSN-overexpressing cells treated with ADR (1 µM and 2 µM, 24 h). **F** Tumorsphere formation assays (day 7) comparing shMSN and MSN-overexpressing cells with or without ADR. Spheres > 100 μm were quantified. Experiments were performed in triplicate (*n* = 3). **G** Western blot of MSN expression in MDA-MB-231 parental cells and drug-resistant cell lines (ADR^r^, CDDP^r^, CBDCA^r^, PTX^r^). GAPDH was used as the loading control. **H** Western blot of MSN expression, 3D spheroid images, and cell viability in parental and ADR-resistant (ADR^r^) cell lines after ADR treatment. IC50 values were calculated. Experiments were performed in triplicate (*n* = 3). **I** Western blot of MSN expression in ADR^r^ cell lines transfected with siMSN or siCON. Cell viability and IC50 values were measured after ADR treatment. Experiments were performed in triplicate (*n* = 3). **J** Secreted IL-6 levels in the supernatant of MDA-MB-231 and ADR^r^ cell lines. Experiments were performed in duplicate (*n* = 2). **K** and **L** Western blot analysis of indicated targets in siMSN-transfected ADR^r^ cells (K) and MDA-MB-231/ADR^r^ cells with or without IL-6 treatment. All data are shown as mean ± SD (C, D, F, H, I, J). Statistical significance was determined by Student’s *t*-test (B, C, D, F, J) and by mixed regression (C, H, I). *P < 0.05, **P < 0.01, ***P < 0.001; ns, not significant

As CSC-like properties are one the major features that induce chemotherapeutic resistance in cancer [29, 30], we next examined the effect of MSN on chemotherapeutic efficacy in TNBC. In the METABRIC TNBC dataset, overexpression of MSN was associated with a worse prognosis for OS and DFS in patients who received conventional chemotherapy (Fig. 6A). Consistently, the analysis of our TNBC patient cohort treated with neoadjuvant chemotherapy also showed significantly higher MSN expression in the non-complete remission (nCR) group compared to the complete remission (CR) group (Fig. 6B), further supporting the evidence that MSN is a potential prognostic marker for poor response to conventional chemotherapy in TNBC. Considering these findings, we next investigated the sensitivity of each chemotherapeutic agent for standard regimen in breast cancer, including Adriamycin, cyclophosphamide, paclitaxel, and docetaxel, upon MSN expression levels (Supplementary Fig.  6 A). As a result, higher MSN expression conferred drug resistance and lower MSN expression indicated increased drug sensitivity to Adriamycin (ADR) solely, but no significant findings in other chemotherapeutic agents were detected. These findings suggest the crucial role of MSN in the therapeutic efficacy of ADR in TNBC. In 3D TNBC cell culture systems, increased MSN expression demonstrated prominent resistance to ADR treatment, with larger spheroids and lower apoptosis rates (Fig. 6C, D and Supplementary Fig. 6B, C). Furthermore, suppression of the previously chosen CSC-related genes, IGFN1, EML1, and SRGN, significantly increased sensitivity to ADR (Supplementary Fig. 6D). Consistently, protein analysis showed reduced activation of PARP and caspase-3 in these cells, suggesting resistance to ADR-induced apoptosis by MSN (Fig. 6E and Supplementary Fig. 6E). The sphere formation assays also indicated that high MSN expression maintained sphere morphology and increased the number of spheres with ADR treatment (Fig. 6F and Supplementary Fig.  6 F). These results suggest that MSN enhances CSC-inducible gene expression and resistance to apoptosis of tumor cells by chemotherapy, contributing to ADR resistance in TNBC. Therefore, we further explored the relationship between chemotherapy resistance and MSN expression using our previously established drug-resistant breast cancer cell lines, such as PTX^r^, CBDCA^r^, CDDP^r^, and ADR^r^. MSN was significantly overexpressed in ADR-resistant breast cancer cells, MDA-MB-231/ADRr and MCF7/ADRr, compared to other drug-resistant cell lines (Fig. 6G and Supplementary Fig. 6G). Also, ADR-resistant cancer cells exhibited more prominent 3D spheroid formation than the paired naive tumor cells with the ADR treatment (Fig. 6H and Supplementary Fig. 6H). Transient knockdown of MSN restored drug sensitivity to ADR in MDA-MB-231/ADR^r^ and MCF7/ADR^r^ (Fig. 6I and Supplementary Fig. 6I). Interestingly, ADR^r^ cells with higher MSN expression secreted significantly increased levels of IL-6 compared to naive tumor cell lines (Fig. 6J). Furthermore, the knockdown of MSN in ADR^r^ cells suppressed STAT3 expression and activation, which was concordant with the condition of IL-6 treatment, where MSN inhibition led to decreased STAT3 phosphorylation (Fig. 6K). ADR^r^ cells also showed increased levels of phosphorylated MSN, CDC42, and PAK4, particularly after IL-6 treatment, compared to parental MDA-MB-231 cell lines (Fig. 6L). These results suggest that ADR^r^ tumor cells secrete more IL-6, which induce phosphorylation of the MSN-CDC42-PAK4-STAT3 pathway, and eventually ADR resistance in TNBC.

### Combinatory Treatment of a STAT3-targeting Inhibitor with Adriamycin Restored Adriamycin Sensitivity in MSN-overexpressed TNBC

**Fig. 7:**
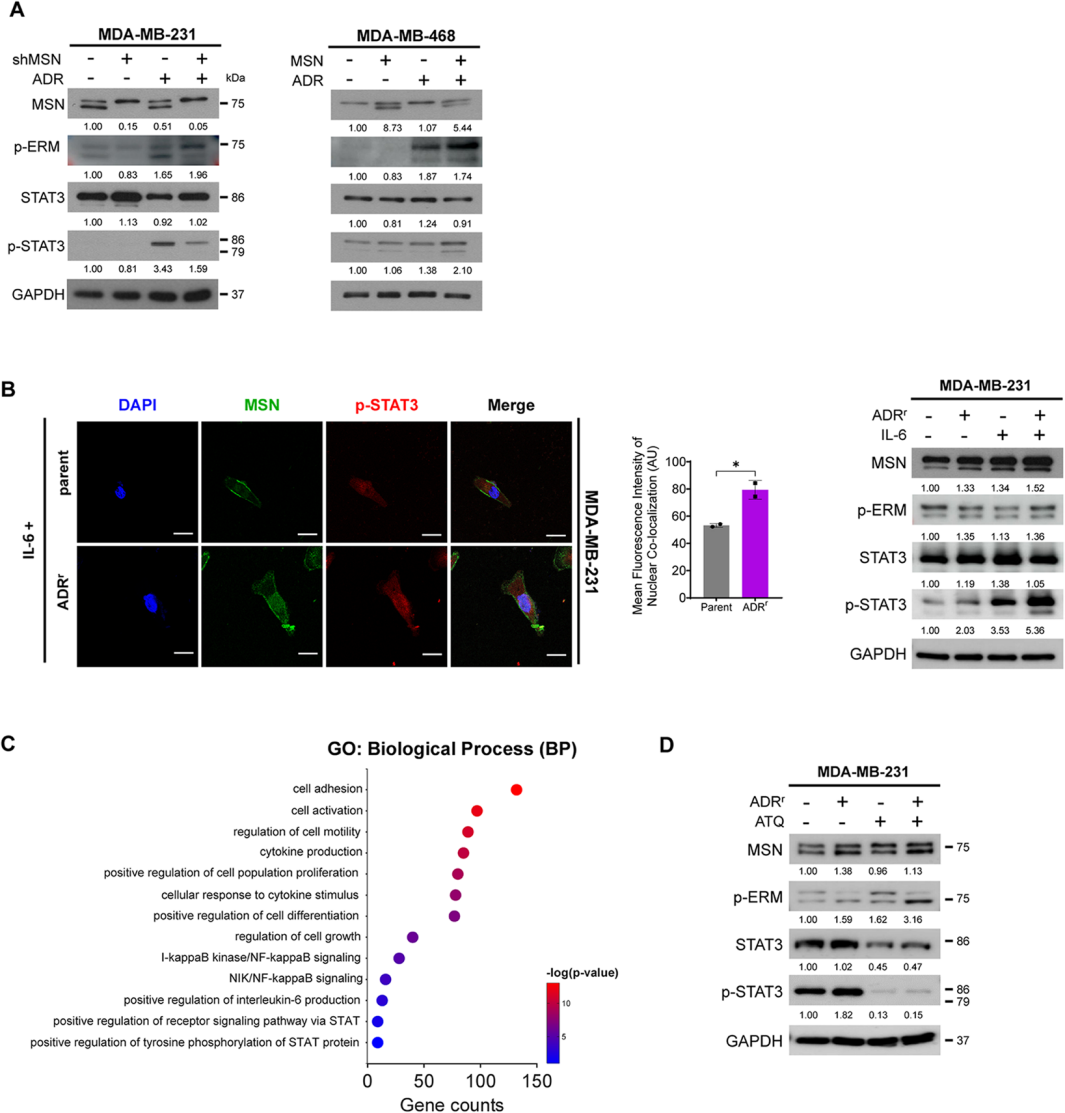

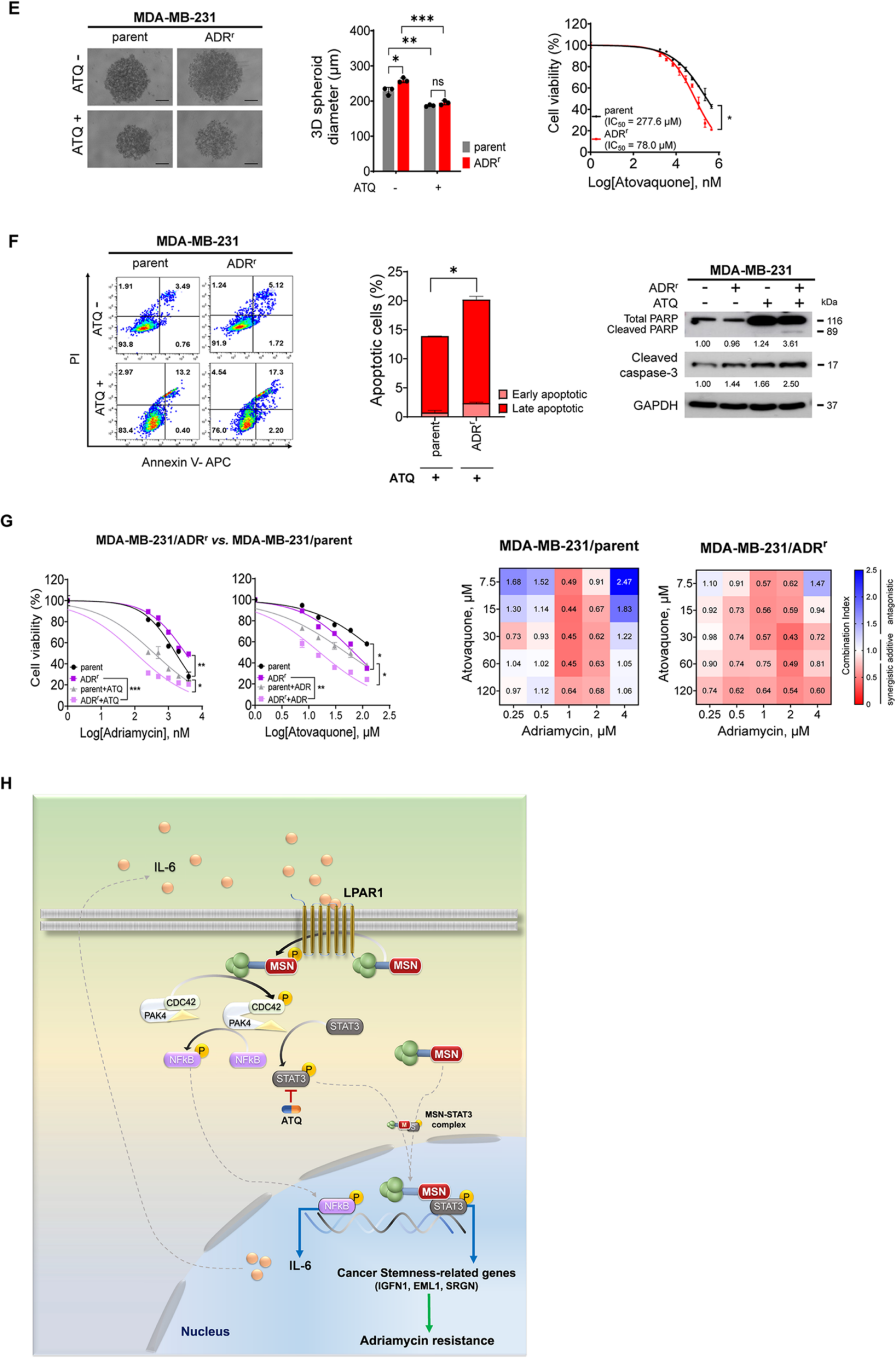
STAT3 Inhibition re-sensitizes MSN-overexpressing TNBC cells to Adriamycin. **A** Western blot analysis of indicated targets in shMSN and MSN-overexpressing cells treated with or without ADR (1 µM and 2 µM, 24 h), compared to control cell lines. **B** Confocal microscopy images of MSN (green), p-STAT3 (red), and DAPI (blue) in MDA-MB-231 parental and ADR-resistant (ADR^r^) cells treated with IL-6. Merged images show MSN and p-STAT3 co-localization. Nuclear co-localization of p-STAT3 and MSN was quantified using ImageJ by measuring overlap with DAPI staining. Bar graphs represent average nuclear co-localization intensity from two independent experiments. Statistical analysis was performed using *t*-tests. Scale bar: 10 μm. Western blot of indicated targets in parental and ADR^r^ cells, with or without IL-6 (50 ng/ml for 5 min). **C** GO enrichment analysis of differentially expressed genes in MDA-MB-231 parental and ADR^r^ cells. Dots represent significantly enriched GO terms (FDR < 0.05). The x-axis shows gene counts, and the y-axis lists GO terms. Dot color indicates -log10 (*p*-value) **D** Western blot analysis of indicated targets in MDA-MB-231 parental and ADR^r^ cells treated with (ATQ, 100 µM for 24 h). **E** 3D spheroid culture of parental and ADR^r^ cells treated with ATQ, with or without ADR. Spheroid diameters and IC50 values for cell viability after ADR treatment were measured. Experiments were performed in triplicate (*n* = 3). **F** Apoptosis analysis of parental and ADR^r^ cells treated with ATQ using Annexin V and PI staining. Apoptotic cell percentages are shown. Experiments were performed in duplicate (*n* = 2). Western blot of indicated targets in parental and ADR^r^ cells treated with or without ATQ. **G** Dose–response curves for ADR and ATQ, alone and in combination, in parental and ADR^r^ cells. Drug synergy analysis (CompuSyn) shows CI values: CI < 1 indicates synergy, CI = 1 additive, and CI > 1 is antagonism. A heatmap presents CI values across doses. Experiments were performed in triplicate (*n* = 3). **H** Schematic model illustrating MSN-driven Adriamycin resistance. MSN phosphorylates NF-κB, promoting IL-6 transcription (blue solid arrow). IL-6 binds to the LPAR1 via an autocrine loop, activating MSN phosphorylation (solid lines). Phosphorylated MSN triggers CDC42-PAK4 complex phosphorylation, which then phosphorylates STAT3. p-STAT3 translocates into the nucleus (dashed arrows), promoting transcription of cancer stem cell-related genes (blue solid arrow), leading to Adriamycin resistance. Co-treatment with STAT3 inhibitor Atovaquone and Adriamycin reverse resistance (green arrow, conclusion). All data are shown as mean ± SD (E, F, G). Statistical significance was determined using Student’s *t*-test (E, F) and mixed regression analysis (E, G). *P < 0.05, **P < 0.01, ***P < 0.001; ns, not significant

Based on the results above, in which overexpression of MSN in ADR-resistant breast cancer cell lines established by long-term ADR treatment and MSN conferred resistance to ADR-based chemotherapy, we hypothesized that ADR might paradoxically lead to drug resistance through the activation of the MSN pathway. ADR treatment showed no alteration of the MSN expression levels but induced phosphorylation of MSN, compared to the group without ADR stimulation, which was similar to STAT3 (Fig. 7A and Supplementary Fig.  7 A). Consistent with our previous results, phosphorylation of STAT3 by MSN was especially prominent with ADR treatment (Fig. 7A and Supplementary Fig.  7 A). In addition, ADR^r^ tumor cells showed more formation of p-STAT3/MSN complex that translocated to the nucleus compared to the TNBC cell lines without ADR resistance and the complex was enhanced by IL-6 stimulation (Fig. 7B). RNA sequencing of ADR^r^ TNBC cells compared to parent cells also revealed increased expression of genes related to STAT3, NF-κB, and cytokine production, explaining this mechanism (Fig. 7C, Supplementary Fig. 7B, and Supplementary Table S13–S15). These findings indicated that ADR-based chemotherapeutic agents might contradictorily induce phosphorylation of the MSN-STAT3 pathway, which accelerates the insensitivity of tumor cells to ADR. Therefore, negative regulation of the MSN-STAT3 pathway could be a promising solution to overcome TNBC refractory to ADR. Due to the absence of a commercially available MSN-targetable agent, we chose a STAT3 inhibitor as an alternative. To investigate whether inhibition of STAT3 activation would increase sensitivity to the drug upon the level of MSN expression, the therapeutic efficacy of the STAT3 inhibitor Atovaquone (ATQ) was tested (Supplementary Fig.  7 C). Treatment with ATQ strongly suppressed the expression and activation of STAT3 in ADR^r^ cells (Fig. 7D). Consequently, ATQ was identified as an effective drug in ADR-resistant TNBC cell lines with high MSN expression, making it a candidate for further research. 3D cell viability and spheroid formation assays demonstrated that ADR-resistant tumor cells exhibited a sensitive response to ATQ treatment, significantly reducing cell viability (*p* = 0.04, Fig. 7E). Additionally, the reduced spheroid size and a more prominent dispersed structure were observed after ATQ treatment in ADR-resistant tumor cells. Notably, the reduction in spheroid size was significantly greater in ADR-resistant cells than in the parental cells following ATQ treatment (*p* = 0.0002 *vs. p* = 0.003, Fig. 7E). Consistently, the activated forms of apoptosis-related proteins, PARP, and caspase-3, were upregulated in ADR-resistant tumor cells following ATQ treatment (Fig. 7F). Finally, we analyzed the synergistic effect of combining ATQ with ADR to determine whether ATQ restores therapeutic sensitivity to ADR, using the CompuSyn software program in ADR-resistant tumor cells (Fig. 7G). ADR-resistant tumor cells exhibited higher cell viability compared to parental cells when treated with ADR alone. However, co-treatment with ATQ restored drug sensitivity in the ADR-resistant cells, leading to a substantial reduction in cell viability relative to parental cells. Conversely, treatment with ATQ alone resulted in lower cell viability in ADR-resistant cells than in parental cells, suggesting inhibition of the MSN-STAT3 pathway. Similarly, drug sensitivity was restored when ATQ was combined with ADR, further reducing cell viability in the ADR-resistant cells (combination of ADR and ATQ *vs*. ATQ alone, *p* = 0.001). These findings suggest that combining the treatment of ADR with the STAT3 inhibitor ATQ could enhance therapeutic efficacy, especially in TNBC patients who suffered from therapeutic resistance to ADR with a higher expression of MSN, indicating a tailored therapeutic strategy.

## Discussion

In this study, we revealed a previously unknown autocrine loop between an extracellular chemokine and an intracellular kinase cascade in TNBC, showing for the first time that IL-6 stimulates MSN phosphorylation and the MSN-induced phosphorylation of NF-κB promotes IL-6 transcription. In addition, the intranuclear translocation of the pSTAT3-MSN complex promotes the transcription of cancer stem cell-related genes, inducing Adriamycin resistance in TNBC (Fig. 7H). In the tumor microenvironment, IL-6 has widely been described as a cytokine that acts as a growth factor for cancer cells, promoting tumorigenesis [31]. For IL-6 activity, binding the secreted ligand to the cell surface receptor extracellular domain is essential [32]. According to their structural features, cytokine receptors are grouped into six major families, and IL-6 typically binds to the hexamers of IL-6/gp130, a class I cytokine receptor complex [33]. However, increasing evidence from recent studies has established the roles of interleukins in cancer progression through interacting with GPCR [34, 35]. Our study also observed that several family genes related to GPCRs were upregulated at the mRNA level in TNBC cell lines after IL-6 treatment, and LPAR1 was selected as the ultimate GPCR-related gene responsive to IL-6 through our multi-stepwise approach. LPAR1 is a type of GPCR mainly activated by binding to a ligand such as lysophosphatidic acid (LPA) in various diseases, including cancer [36, 37]. Although it has been unclear whether LPAR serves the receptor of IL-6 and is a secondary event to the direct binding and alternative activation within the cytoplasm, indirect evidence suggests LPA binding to LPAR stimulates IL-6 expression [38]. For the first time, our data demonstrated interactions among those signaling cascades, where IL-6 acts as a ligand to LPAR1 and sequentially activates MSN by phosphorylation in TNBC. These findings were similarly observed in ovarian cancer, where G-coupled LPAR activation also induced ERM protein phosphorylation [39]. ERM protein binds various transmembrane proteins with the FERM domain and mainly anchors the protein to the intracytoplasmic cytoskeleton [40]. However, growing evidence has unveiled the interaction with non-canonical functional units, such as transmitting signals from cell membranes towards the cytoplasm [41]. In our study, MSN concordantly functioned as a linker of LPAR1 to RHO GTPases and their downstream effectors, a previously unknown finding. The GPCR-induced Rho-dependent signaling cascade has recently emerged as a key player in tumor growth and metastasis [42]. We established another novel signaling mechanism, where MSN, among the Rho-type GTPases, altered the phosphorylation of CDC42 and PAK4, a downstream effector of CDC42, [43, 44] in multiple TNBC cell lines. To date, 20 canonical members of the RHO family, including RHOA, RAC1, and CDC42, have been identified; these interact with over 70 downstream effectors in humans [45]. Previous X-ray crystallography experiments revealed that the polybasic region of CDC42 is structurally bound to the PAK4 kinase domain, while the dimeric structure changes during the interaction of the extracellular environment with intracellular signal transduction pathways [46]. Along with the functions of MSN as a phosphate messenger between LPAR1 and GTPase, we found additional novel features of MSN as an intracellular transporter that transports STAT3 into the nuclei of TNBC cells, which is crucial for the transcription of specific genes in cancer. The phosphorylated STAT3 by the CDC42-PAK4 complex protein is translocated into the nucleus by directly binding with the cytoplasm's MSN protein. In a recent Drosophila study, MSN among ERM complexes particularly entered the nucleus from the cytoplasm through a nuclear localization signal (NLS)-dependent mechanism, functioning as an active nuclear transporter [47, 48]. In addition, the results provided further evidence that the non-phosphorylated MSN might be favored regarding nuclear transport, compared to its phosphorylated form [47]. In agreement with these results, we also observed that non-phosphorylated MSN binds to and translocates pSTAT3 into the nucleus. These novel findings are non-canonical signaling cascades of STAT3 phosphorylation, compared to the classic signaling discovered so far, where JAK phosphorylates STAT3 and triggers nuclear translocation of p-STAT3 without a physical binding together [49, 50]. These findings reveal that MSN functions not only as a cytoskeletal adaptor but also as a previously unrecognized scaffolding protein that facilitates STAT3 nuclear translocation in a phosphorylation-dependent manner. This role of MSN distinguishes it from canonical STAT3 regulators, positioning it as a critical integrator of IL-6/NF-κB signaling in the chemoresistance circuitry of TNBC.

Even though JAK and MSN share the structural similarity of the N-terminal FERM domain, the mode of action for activating STAT3 may differ. STAT3 is a family of transcription factors [51] found to play a crucial role in maintaining cancer stem cells [52]. Our data also identified altered expression of more than 1,000 genes related to cancer stemness by the complex of MSN-pSTAT3 transcription factor, eventually presenting three genes including IGFN1 [53–55], EML1, and SRGN [56–58] after stepwise filtering platforms with clinical public databases and various sets of in vitro tests. Unlike IGFN1 and SRGN, which were revealed as novel genes related to cancer stemness, EML1 is thought to be mainly responsible for physiologic stemness, such as embryogenesis of cilia formation or brain formation, yet there is no evidence of its role in cancer stemness [59, 60]. Our findings describe, for the first time, a tumor setting where EML1 may act as a novel gene for the stem cell-like phenotype of cancer cells. These molecular pathways eventually cause chemotherapeutic resistance, especially against Adriamycin, as shown in our TNBC cell line models. Although the molecular mechanism remains largely unknown, Adriamycin-induced paradoxical tumor growth and drug resistance may be led by persistent STAT3 activity [61]. Along these lines, nanosized polymeric carriers for simultaneous Adriamycin/STAT3 siRNA delivery were suggested as an effective strategy to increase ADR-mediated cell death in TNBC cell lines [62]. We presented Atovaquone, a STAT3 inhibitor, as a therapeutic strategy to enhance the anti-cancer effect for Adriamycin-resistant TNBC. Atovaquone, an antimalarial medication, is a potent selective STAT3 inhibitor approved by the US Food and Drug Administration (FDA) and has recently been repurposed as an antitumor therapeutic option [63]. In previous studies, ATQ effectively reduced the growth of paclitaxel-resistant rodent breast tumors and was suggested as a novel therapeutic drug in hematopoietic malignancies [64, 65]. Our finding is the first evidence of the therapeutic efficacy of Atovaquone in MSN/STAT3-induced Adriamycin-resistant TNBC. However, it is important to acknowledge that Atovaquone may exert pleiotropic effects beyond STAT3 inhibition. Prior studies have shown that it can disrupt mitochondrial oxidative phosphorylation and affect cellular energy metabolism [66–68]. Therefore, although our data suggest that the anti-tumor activity of Atovaquone in TNBC is mediated by suppression of the STAT3 pathway, we cannot rule out the contribution of off-target mechanisms. These potential off-target effects should be considered when interpreting the therapeutic implications of Atovaquone and underscore the need for further mechanistic studies.

In conclusion, our study delineates the crucial role of MSN in TNBC tumor initiation and chemotherapy resistance, which is closely linked to the signaling cascade initiated by IL-6 binding to LPAR1. This cascade sequentially phosphorylates MSN and a complex of CDC42 and PAK4, which then phosphorylates STAT3. Subsequently, pSTAT3 translocates into the nucleus and promotes transcription of genes related to cancer stemness. Along with STAT3, MSN-dependent Rho-type GTPases also phosphorylate NF-κB that enters into the nucleus and binds to the transcription factor site of IL-6, eventually creating the autocrine loop between extracellular IL-6 stimulation and activation of an MSN-dependent kinase pathway, which is associated with Adriamycin resistance in TNBC. The drug repositioning of Atovaquone might be a promising inhibitor-based therapeutic strategy tailored to Adriamycin refractory TNBC patients.

## Supplementary Information

Additional file 1.

Additional file 2.

Additional file 3.

Additional file 4.

Additional file 5.

Additional file 6.

Additional file 7.

Additional file 8.

Additional file 9.

Additional file 10.

Additional file 11.

Additional file 12.

Additional file 13.

Additional file 14.

## Data Availability

The raw RNA sequencing data supporting the conclusions of this article are deposited in the Gene Expression Omnibus (GEO) database (https://www.ncbi.nlm.nih.gov/geo) at the National Center for Biotechnology Information (NCBI) under the accession numbers GEO272103 and GEO272101. These datasets can be accessed directly via their persistent identifiers. For further information, please contact the corresponding author.

## References

[1] Wu J, Li S, Jia W, Su F. Response and prognosis of taxanes and anthracyclines neoadjuvant chemotherapy in patients with triple-negative breast cancer. J Cancer Res Clin Oncol. 2011;137(10):1505–10. 21830158 10.1007/s00432-011-1029-6 PMC11827805

[2] Hanahan D. Hallmarks of cancer: new dimensions. Cancer Discov. 2022;12(1):31–46. 35022204 10.1158/2159-8290.CD-21-1059

[3] Sp N, Kang DY, Kim DH, Park JH, Lee HG, Kim HJ, Darvin P, Park Y-M, Yang YM. Nobiletin inhibits CD36-dependent tumor angiogenesis, migration, invasion, and sphere formation through the Cd36/Stat3/Nf-Κb signaling axis. Nutrients. 2018;10(6):772. 29914089 10.3390/nu10060772 PMC6024609

[4] Ono M, Kosaka N, Tominaga N, Yoshioka Y, Takeshita F, Takahashi R-u, Yoshida M, Tsuda H, Tamura K, Ochiya T.Exosomes from bone marrow mesenchymal stem cells contain a microRNA that promotes dormancy in metastatic breast cancer cells. Science signaling. 2014; 7(332):ra63-ra. 10.1126/scisignal.2005231 24985346

[5] Hu X, Li J, Fu M, Zhao X, Wang W. The JAK/STAT signaling pathway: from bench to clinic. Signal Transduct Target Ther. 2021;6(1):402. 34824210 10.1038/s41392-021-00791-1 PMC8617206

[6] Calon A, Espinet E, Palomo-Ponce S, Tauriello DV, Iglesias M, Céspedes MV, Sevillano M, Nadal C, Jung P, Zhang XH-F. Dependency of colorectal cancer on a TGF-β-driven program in stromal cells for metastasis initiation. Cancer Cell. 2012; 22(5):571–84. 10.1016/j.ccr.2012.08.013 PMC3512565 23153532

[7] Thakur R, Trivedi R, Rastogi N, Singh M, Mishra DP. Inhibition of STAT3, FAK and Src mediated signaling reduces cancer stem cell load, tumorigenic potential and metastasis in breast cancer. Sci Rep. 2015;5(1):10194. 25973915 10.1038/srep10194 PMC4431480

[8] Shih P-C, Mei K-C. Role of STAT3 signaling transduction pathways in cancer stem cell-associated chemoresistance. Drug Discovery Today. 2021;26(6):1450–8. 33307211 10.1016/j.drudis.2020.11.032

[9] Dolatabadi S, Jonasson E, Lindén M, Fereydouni B, Bäcksten K, Nilsson M, Martner A, Forootan A, Fagman H, Landberg G. JAK–STAT signalling controls cancer stem cell properties including chemotherapy resistance in myxoid liposarcoma. Int. J. Cancer. 2019; 145(2):435–49. 10.1002/ijc.32123 PMC6590236 30650179

[10] Park JH, Lee C, Han D, Lee JS, Lee KM, Song MJ, Kim K, Lee H, Moon KC, Kim Y. Moesin (MSN) as a novel proteome-based diagnostic marker for early detection of invasive bladder urothelial carcinoma in liquid-based cytology. Cancers (Basel). 2020;12(4):1018. 32326232 10.3390/cancers12041018 PMC7225967

[11] Du Y, Bradshaw WJ, Leisner TM, Annor-Gyamfi JK, Qian K, Bashore FM, Sikdar A, Nwogbo FO, Ivanov AA, Frye SV, et al. Discovery of FERM domain protein-protein interaction inhibitors for MSN and CD44 as a potential therapeutic approach for Alzheimer’s disease. J Biol Chem. 2023;299(12):105382. 37866628 10.1016/j.jbc.2023.105382 PMC10692723

[12] Ferrao R, Lupardus PJ. The Janus Kinase (JAK) FERM and SH2 Domains: Bringing Specificity to JAK–Receptor Interactions. Front. Endocrinol. 2017;8:71. 10.3389/fendo.2017.00071 PMC5394478 28458652

[13] Mengie Ayele T, Tilahun Muche Z, Behaile Teklemariam A, Bogale Kassie A, Chekol AE. Role of JAK2/STAT3 Signaling Pathway in the Tumorigenesis, Chemotherapy Resistance, and Treatment of Solid Tumors: A Systemic Review. J Inflamm Res. 2022;15:1349–64. 35241923 10.2147/JIR.S353489 PMC8887966

[14] Giuliano AE, Edge SB, Hortobagyi GN. Eighth Edition of the AJCC Cancer Staging Manual: Breast Cancer. Ann Surg Oncol. 2018;25(7):1783–5. 29671136 10.1245/s10434-018-6486-6

[15] Symmans WF, Peintinger F, Hatzis C, Rajan R, Kuerer H, Valero V, Assad L, Poniecka A, Hennessy B, Green M, et al. Measurement of residual breast cancer burden to predict survival after neoadjuvant chemotherapy. J Clin Oncol. 2007;25(28):4414–22. 17785706 10.1200/JCO.2007.10.6823

[16] McCarty Jr K, Miller L, Cox E, Konrath J, McCarty Sr K.Estrogen receptor analyses. Correlation of biochemical and immunohistochemical methods using monoclonal antireceptor antibodies. Arch. Pathol. Lab. Med. 1985; 109(8):716–21. 3893381

[17] Firdous S, Ghosh A, Saha S.BCSCdb: a database of biomarkers of cancer stem cells. Database. 2022; 2022:baac082. 10.1093/database/baac082 PMC9517164 36169329

[18] Fehon RG, McClatchey AI, Bretscher A. Organizing the cell cortex: the role of ERM proteins. Nat Rev Mol Cell Biol. 2010;11(4):276–87. 20308985 10.1038/nrm2866 PMC2871950

[19] Johnson DE, O’Keefe RA, Grandis JR. Targeting the IL-6/JAK/STAT3 signalling axis in cancer. Nat Rev Clin Oncol. 2018;15(4):234–48. 29405201 10.1038/nrclinonc.2018.8 PMC5858971

[20] Zhang N, Zeng Y, Du W, Zhu J, Shen D, Liu Z, Huang J-A. The EGFR pathway is involved in the regulation of PD-L1 expression via the IL-6/JAK/STAT3 signaling pathway in EGFR-mutated non-small cell lung cancer. Int J Oncol. 2016;49(4):1360–8. 27499357 10.3892/ijo.2016.3632

[21] Matsusaka T, Fujikawa K, Nishio Y, Mukaida N, Matsushima K, Kishimoto T, Akira S. Transcription factors NF-IL6 and NF-kappa B synergistically activate transcription of the inflammatory cytokines, interleukin 6 and interleukin 8. Proc Natl Acad Sci. 1993;90(21):10193–7. 8234276 10.1073/pnas.90.21.10193 PMC47740

[22] Wolf J, Rose-John S, Garbers C. Interleukin-6 and its receptors: a highly regulated and dynamic system. Cytokine. 2014;70(1):11–20. 24986424 10.1016/j.cyto.2014.05.024

[23] Cant SH, Pitcher JA. G protein-coupled receptor kinase 2-mediated phosphorylation of ezrin is required for G protein-coupled receptor-dependent reorganization of the actin cytoskeleton. Mol. Biol. Cell. 2005; 16(7):3088–99. 10.1091/mbc.E04-10-0877 PMC1165394 15843435

[24] Gustavsson M. New insights into the structure and function of chemokine receptor: chemokine complexes from an experimental perspective. J Leukoc Biol. 2020;107(6):1115–22. 31965639 10.1002/JLB.2MR1219-288R

[25] Olivia MY, Brown JH. G Protein–coupled receptor and RhoA-stimulated transcriptional responses: links to inflammation, differentiation, and cell proliferation. Mol. Pharmacol. 2015; 88(1):171–80. 10.1124/mol.115.097857 PMC4468647 25904553

[26] Wang S-W, Sun Y-M. The IL-6/JAK/STAT3 pathway: potential therapeutic strategies in treating colorectal cancer. Int J Oncol. 2014;44(4):1032–40. 24430672 10.3892/ijo.2014.2259

[27] Ferrao R, Lupardus PJ. The Janus kinase (JAK) FERM and SH2 domains: Bringing specificity to JAK–receptor interactions. Front Endocrinol. 2017;8:71. 10.3389/fendo.2017.00071 PMC5394478 28458652

[28] Huang B, Lang X, Li X. The role of IL-6/JAK2/STAT3 signaling pathway in cancers. Front Oncol. 2022;12:1023177. 36591515 10.3389/fonc.2022.1023177 PMC9800921

[29] He L, Wick N, Germans SK, Peng Y. The Role of Breast Cancer Stem Cells in Chemoresistance and Metastasis in Triple-Negative Breast Cancer. Cancers (Basel). 2021;13(24):6209. 34944829 10.3390/cancers13246209 PMC8699562

[30] Toledo B, González-Titos A, Hernández-Camarero P, Perán M. A Brief Review on Chemoresistance; Targeting Cancer Stem Cells as an Alternative Approach. Int. J. Mol. Sci. 2023; 24(5):4487. 10.3390/ijms24054487 PMC10003376 36901917

[31] Orange ST, Leslie J, Ross M, Mann DA, Wackerhage H. The exercise IL-6 enigma in cancer. Trends Endocrinol Metab. 2023;34(11):749-63. 10.1016/j.tem.2023.08.001 37633799

[32] Wang X, Lupardus P, LaPorte SL, Garcia KC. Structural biology of shared cytokine receptors. Annu Rev Immunol. 2009;27(1):29–60. 18817510 10.1146/annurev.immunol.24.021605.090616 PMC3981547

[33] Boulanger MJ, Chow D-c, Brevnova EE, Garcia KC. Hexameric structure and assembly of the interleukin-6/IL-6 α-receptor/gp130 complex. Science. 2003; 300(5628):2101–4. 10.1126/science.1083901 12829785

[34] Natsagdorj A, Izumi K, Hiratsuka K, Machioka K, Iwamoto H, Naito R, Makino T, Kadomoto S, Shigehara K, Kadono Y. CCL2 induces resistance to the antiproliferative effect of cabazitaxel in prostate cancer cells. Cancer Sci. 2019; 110(1):279–88. 10.1111/cas.13876 PMC6317938 30426599

[35] Bonapace L, Coissieux M-M, Wyckoff J, Mertz KD, Varga Z, Junt T, Bentires-Alj M. Cessation of CCL2 inhibition accelerates breast cancer metastasis by promoting angiogenesis. Nature. 2014;515(7525):130–3. 25337873 10.1038/nature13862

[36] Moolenaar WH. Regulation and biological activities of the autotaxin-LPA axis. Chem Phys Lipids. 2009;160:S12. 10.1016/j.plipres.2007.02.001 17459484

[37] Aikawa S, Hashimoto T, Kano K, Aoki J. Lysophosphatidic acid as a lipid mediator with multiple biological actions. The journal of biochemistry. 2015;157(2):81–9. 25500504 10.1093/jb/mvu077

[38] Hartman ZC, Poage GM, Den Hollander P, Tsimelzon A, Hill J, Panupinthu N, Zhang Y, Mazumdar A, Hilsenbeck SG, Mills GB. Growth of triple-negative breast cancer cells relies upon coordinate autocrine expression of the proinflammatory cytokines IL-6 and IL-8. Cancer Res. 2013;73(11):3470–80. 23633491 10.1158/0008-5472.CAN-12-4524-T PMC3853111

[39] Park J, Jang J-H, Oh S, Kim M, Shin C, Jeong M, Heo K, Park JB, Kim SR, Oh Y-S. LPA-induced migration of ovarian cancer cells requires activation of ERM proteins via LPA1 and LPA2. Cell. Signal. 2018; 44:138–47. 10.1016/j.cellsig.2018.01.007 29329782

[40] Tsukita S, Yonemura S, Tsukita S. ERM (ezrin/radixin/moesin) family: from cytoskeleton to signal transduction. Curr. Opin. Cell Biol. 1997; 9(1):70–5. 10.1016/s0955-0674(97)80154-8 9013673

[41] García-Ortiz A, Serrador JM. ERM proteins at the crossroad of leukocyte polarization, migration and intercellular adhesion. Int J Mol Sci. 2020;21(4):1502. 32098334 10.3390/ijms21041502 PMC7073024

[42] Dorsam RT, Gutkind JS. G-protein-coupled receptors and cancer. Nature reviews cancer. 2007;7(2):79–94. 17251915 10.1038/nrc2069

[43] Bokoch GM. Biology of the p21-activated kinases. Annu Rev Biochem. 2003;72(1):743–81. 12676796 10.1146/annurev.biochem.72.121801.161742

[44] Vadlamudi RK, Adam L, Wang R-A, Mandal M, Nguyen D, Sahin A, Chernoff J, Hung M-C, Kumar R. Regulatable expression of p21-activated kinase-1 promotes anchorage-independent growth and abnormal organization of mitotic spindles in human epithelial breast cancer cells. J Biol Chem. 2000;275(46):36238–44. 10945974 10.1074/jbc.M002138200

[45] Mosaddeghzadeh N, Ahmadian MR. The RHO Family GTPases: Mechanisms of Regulation and Signaling. Cells. 2021;10(7):1831. 34359999 10.3390/cells10071831 PMC8305018

[46] Ha BH, Boggon TJ. CDC42 binds PAK4 via an extended GTPase-effector interface. Proceedings of the National Academy of Sciences. 2018; 115(3):531–6. 10.1073/pnas.1717437115 PMC5776996 29295922

[47] Kovács Z, Bajusz C, Szabó A, Borkúti P, Vedelek B, Benke R, Lipinszki Z, Kristó I, Vilmos P. A bipartite NLS motif mediates the nuclear import of Drosophila moesin. Frontiers in Cell and Developmental Biology. 2024; 12:1206067. 10.3389/fcell.2024.1206067 PMC10915024 38450250

[48] Kristó I, Bajusz C, Borsos BN, Pankotai T, Dopie J, Jankovics F, Vartiainen MK, Erdélyi M, Vilmos P. The actin binding cytoskeletal protein Moesin is involved in nuclear mRNA export. Biochimica et Biophysica Acta (BBA)-Molecular Cell Research. 2017; 1864(10):1589–604. 10.1016/j.bbamcr.2017.05.020 28554770

[49] Kaptein A, Paillard V, Saunders M. Dominant Negative Stat3 Mutant Inhibits Interleukin-6-induced Jak-STAT Signal Transduction (∗). J Biol Chem. 1996;271(11):5961–4. 8626374 10.1074/jbc.271.11.5961

[50] Shuai K, Ziemiecki A, Wilks AF, Harpur AG, Sadowski HB, Gilman MZ, Darnell JE. Polypeptide signalling to the nucleus through tyrosine phosphorylation of Jak and Stat proteins. Nature. 1993;366(6455):580–3. 7504784 10.1038/366580a0

[51] Darnell Jr JE, Kerr lM, Stark GR. Jak-STAT pathways and transcriptional activation in response to IFNs and other extracellular signaling proteins. Science. 1994; 264(5164):1415–21. 10.1126/science.8197455 8197455

[52] Zhou J, Wulfkuhle J, Zhang H, Gu P, Yang Y, Deng J, Margolick JB, Liotta LA, Petricoin E III, Zhang Y. Activation of the PTEN/mTOR/STAT3 pathway in breast cancer stem-like cells is required for viability and maintenance. Proc Natl Acad Sci. 2007;104(41):16158–63. 17911267 10.1073/pnas.0702596104 PMC2042178

[53] Lu C-H, Wei S-T, Liu J-J, Chang Y-J, Lin Y-F, Yu C-S, Chang SL-Y. Recognition of a novel gene signature for human glioblastoma. Int. J. Mol. Sci. 2022; 23(8):4157. 10.3390/ijms23084157 PMC9029857 35456975

[54] Ma Q, Geng K, Xiao P, Zeng L. Identification and Prognostic Value Exploration of Radiotherapy Sensitivity-Associated Genes in Non-Small-Cell Lung Cancer. Biomed Res Int. 2021;2021(1):5963868. 34518802 10.1155/2021/5963868 PMC8433590

[55] Lefebvre C, Bachelot T, Filleron T, Pedrero M, Campone M, Soria J-C, Massard C, Levy C, Arnedos M, Lacroix-Triki M. Mutational profile of metastatic breast cancers: a retrospective analysis. PLoS Med. 2016;13(12): e1002201. 28027327 10.1371/journal.pmed.1002201 PMC5189935

[56] Zhang Z, Qiu N, Yin J, Zhang J, Liu H, Guo W, Liu M, Liu T, Chen D, Luo K. SRGN crosstalks with YAP to maintain chemoresistance and stemness in breast cancer cells by modulating HDAC2 expression. Theranostics. 2020; 10(10):4290. 10.7150/thno.41008 PMC7150493 32292495

[57] Du Q, Yuan Z, Huang X, Huang Y, Zhang J, Li R. miR-26b-5p suppresses chemoresistance in breast cancer by targeting serglycin. Anticancer Drugs. 2022; 33(3):308–19. 10.1097/CAD.0000000000001268 PMC8812413 34924500

[58] Zhang Z, Deng Y, Zheng G, Jia X, Xiong Y, Luo K, Qiu Q, Qiu N, Yin J, Lu M. SRGN-TGFβ2 regulatory loop confers invasion and metastasis in triple-negative breast cancer. Oncogenesis. 2017; 6(7):e360-e. 10.1038/oncsis.2017.53 PMC5541705 28692037

[59] Jabali A, Hoffrichter A, Uzquiano A, Marsoner F, Wilkens R, Siekmann M, Bohl B, Rossetti AC, Horschitz S, Koch P. Human cerebral organoids reveal progenitor pathology in EML1-linked cortical malformation. EMBO Rep. 2022;23(5):e54027. 35289477 10.15252/embr.202154027 PMC9066063

[60] Uzquiano A, Cifuentes-Diaz C, Jabali A, Romero DM, Houllier A, Dingli F, Maillard C, Boland A, Deleuze J-F, Loew D. Mutations in the heterotopia gene Eml1/EML1 severely disrupt the formation of primary cilia. Cell Rep. 2019; 28(6):1596–611. e10. 10.1016/j.celrep.2019.06.096 31390572

[61] Zhou J, Ong C-N, Hur G-M, Shen H-M. Inhibition of the JAK-STAT3 pathway by andrographolide enhances chemosensitivity of cancer cells to doxorubicin. Biochem Pharmacol. 2010;79(9):1242–50. 20026083 10.1016/j.bcp.2009.12.014

[62] Kim J-H, Lee SC, Ro J, Kang HS, Kim HS, Yoon S. Jnk signaling pathway-mediated regulation of Stat3 activation is linked to the development of doxorubicin resistance in cancer cell lines. Biochem Pharmacol. 2010;79(3):373–80. 19766599 10.1016/j.bcp.2009.09.008

[63] Cheng G, Hardy M, Topchyan P, Zander R, Volberding P, Cui W, Kalyanaraman B. Potent inhibition of tumour cell proliferation and immunoregulatory function by mitochondria-targeted atovaquone. Sci Rep. 2020;10(1):17872. 33087770 10.1038/s41598-020-74808-0 PMC7578061

[64] Gupta N, Srivastava SK. Atovaquone: an antiprotozoal drug suppresses primary and resistant breast tumor growth by inhibiting HER2/β-catenin signaling. Mol Cancer Ther. 2019;18(10):1708–20. 31270151 10.1158/1535-7163.MCT-18-1286 PMC6905100

[65] Xiang M, Kim H, Ho VT, Walker SR, Bar-Natan M, Anahtar M, Liu S, Toniolo PA, Kroll Y, Jones N. Gene expression–based discovery of atovaquone as a STAT3 inhibitor and anticancer agent. Blood, The Journal of the American Society of Hematology. 2016;128(14):1845–53. 10.1182/blood-2015-07-660506 PMC5054697 27531676

[66] Liu B, Zheng X, Li J, Yao P, Guo P, Liu W, Zhao G. Atovaquone inhibits colorectal cancer metastasis by regulating PDGFRβ/NF-κB signaling pathway. BMC Cancer. 2023;23(1):1070. 37932661 10.1186/s12885-023-11585-9 PMC10629062

[67] Kapur A, Mehta P, Simmons AD, Ericksen SS, Mehta G, Palecek SP, Felder M, Stenerson Z, Nayak A, Dominguez JMA, et al. Atovaquone: An Inhibitor of Oxidative Phosphorylation as Studied in Gynecologic Cancers. Cancers. 2022;14(9):2297. 35565426 10.3390/cancers14092297 PMC9102822

[68] Fiorillo M, Lamb R, Tanowitz HB, Mutti L, Krstic-Demonacos M, Cappello AR, Martinez-Outschoorn UE, Sotgia F, Lisanti MP. Repurposing atovaquone: targeting mitochondrial complex III and OXPHOS to eradicate cancer stem cells. Oncotarget. 2016;7(23):34084–99. 27136895 10.18632/oncotarget.9122 PMC5085139

