# Supplementary material for: MSN/STAT3 drives cancer stemness and chemoresistance via IL-6/LPAR1 ligand receptor complex in triple-negative breast cancer
Source: Breast Cancer Res. 2025 Jul 22;27:136. doi: 10.1186/s13058-025-02072-z (PMC12281688; doi:10.1186/s13058-025-02072-z)
Supplement: Supplementary file 1 — Additional file 1. [file 13058_2025_2072_MOESM1_ESM.docx]

**
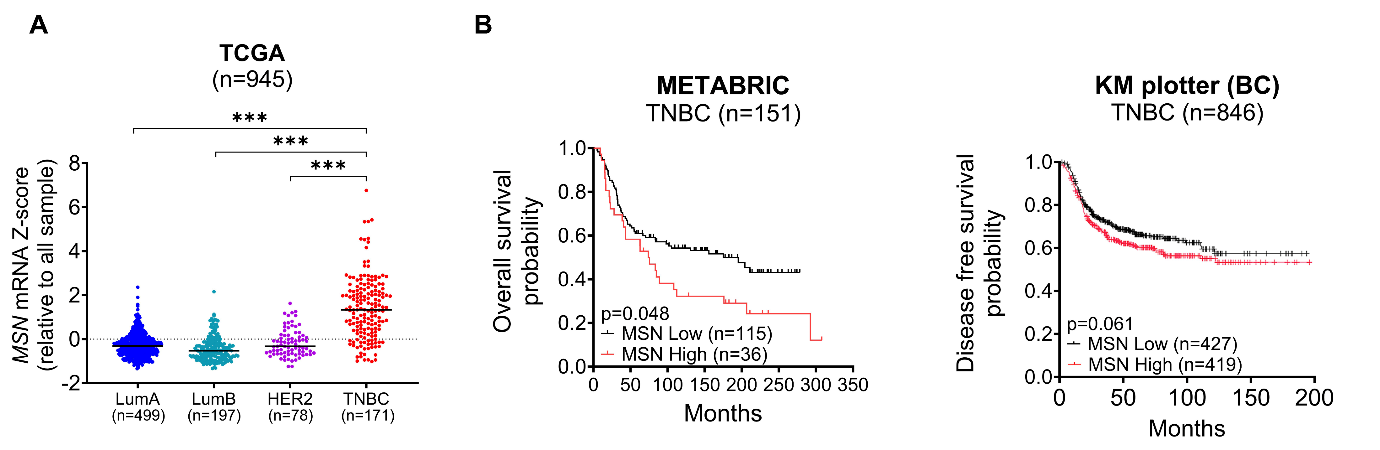
**

**Supplementary Figure 1. High MSN Expression Correlates with Poor Survival and Clinical Outcomes in TNBC Patients. A** MSN mRNA expression across different breast cancer subtypes: Luminal A, Luminal B, HER2-enriched, and TNBC from the TCGA dataset. **B** Kaplan–Meier survival analysis of TNBC patients from the METABRIC dataset stratified by high- and low-MSN expression for overall survival (left) and disease-free survival (right). KM plotter TNBC dataset for DFS. For panel A, statistical significance was assessed using one-way ANOVA followed by Tukey’s multiple comparisons test, with median MSN expression as the cut-off. ***P < 0.001.


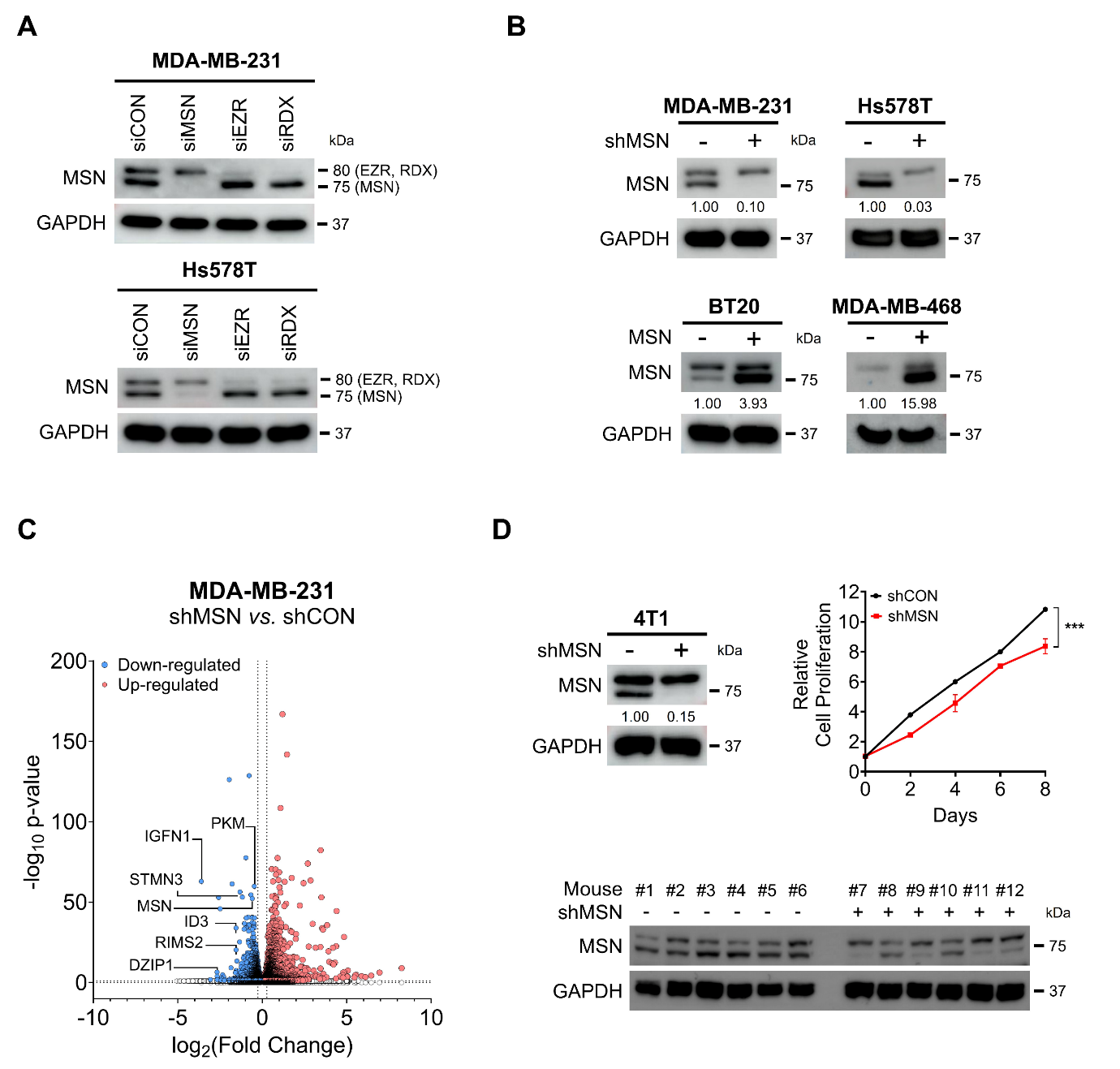


**Supplementary Figure 2. Effects of MSN Knockdown and Overexpression on TNBC and 4T1 Cell Lines. A** Western blot analysis of ERM component knockdown in MDA-MB-231 and Hs578T cells using siRNAs targeting ERM components (siMSN, siEZR, siRDX) and a control siRNA (siCON). Knockdown efficiency and protein expression were analyzed. **B** Western blot of MSN overexpression and knockdown in TNBC cell lines (BT20, MDA-MB-468 for overexpression; MDA-MB-231, Hs578T for knockdown). Lentiviral transduction was used. **C** Volcano plot from RNA-seq analysis comparing gene expression between shMSN and shCON in MDA-MB-231 cells, showing differentially expressed genes. **D** Proliferation assays and Western blot of 4T1 cells (shMSN vs. shCON) showing the effects of MSN knockdown on tumor growth. All experiments were performed in triplicate (*n* = 3), and statistical significance was assessed using mixed regression analysis. ***P < 0.001. In vivo, tumor analysis confirmed MSN expression in 4T1-derived tumors.

**
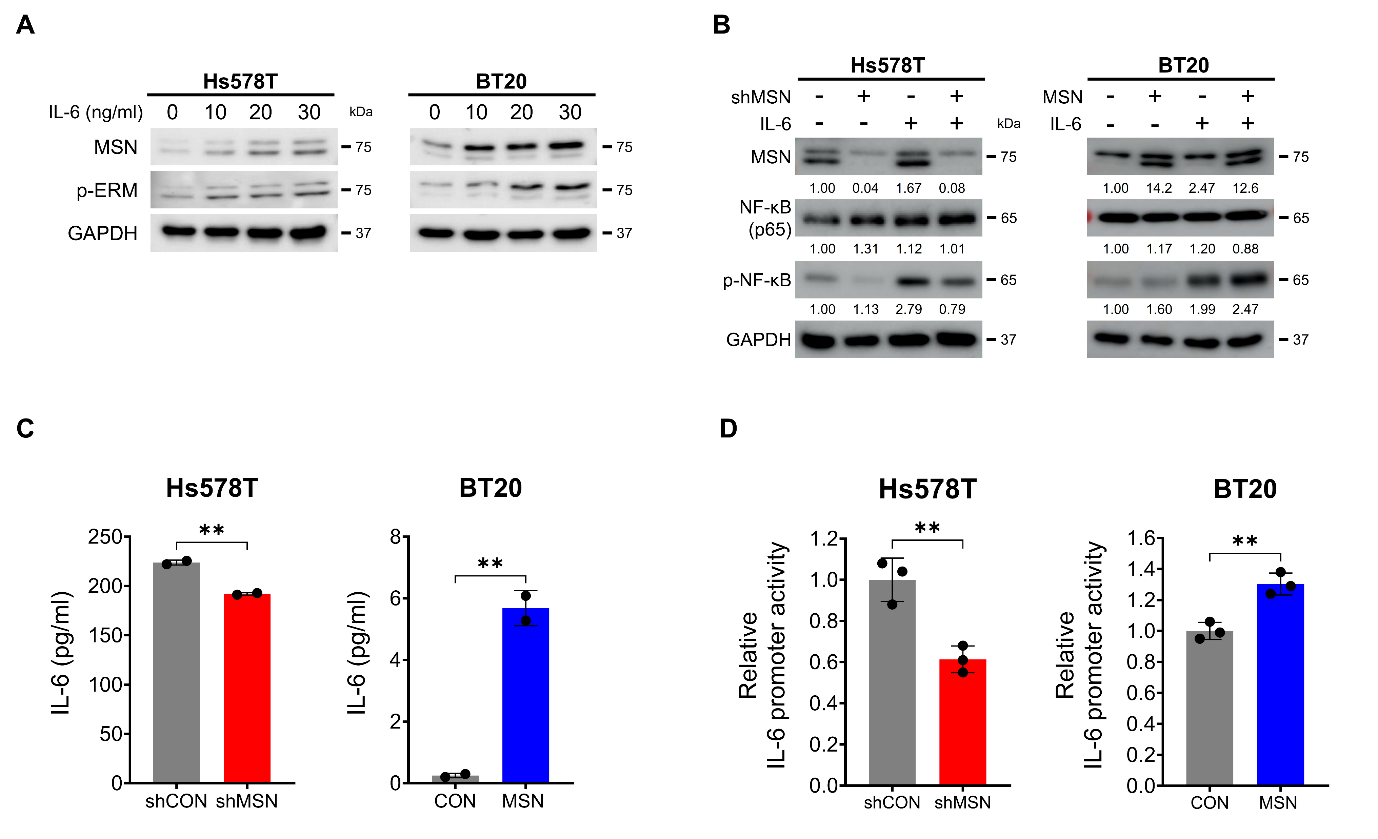
****Supplementary Figure 3. IL-6-Mediated Activation of MSN Promotes a Feedback Loop in IL-6 Transcription and NF-κB Phosphorylation. A** Western blot analysis of MSN and p-ERM in Hs578T and BT20 cells after dose-dependent IL-6 treatment (0, 10, 20, 30 ng/ml). GAPDH was used as an internal loading control. **B** Western blot of MSN, NF-κB, and p-NF-κB in Hs578T/shMSN and BT20/MSN cell lines, with or without IL-6 treatment. **C** Bar graph showing secreted IL-6 levels by ELISA in Hs578T/shMSN and BT20/MSN cells. Experiments were performed in duplicate (*n* = 2), and statistical significance was assessed using unpaired two-tailed *t*-tests (shMSN vs. shCON and MSN vs. CON). **D** Dual-Luciferase Reporter Assay measuring IL-6 promoter activity in Hs578T/shMSN and BT20/MSN cells. Promoter activity was measured using constructs containing NF-κB and STAT3 binding motifs. Experiments were performed in triplicate (*n* = 3), and statistical significance was determined using unpaired two-tailed *t*-tests. **P < 0.01.


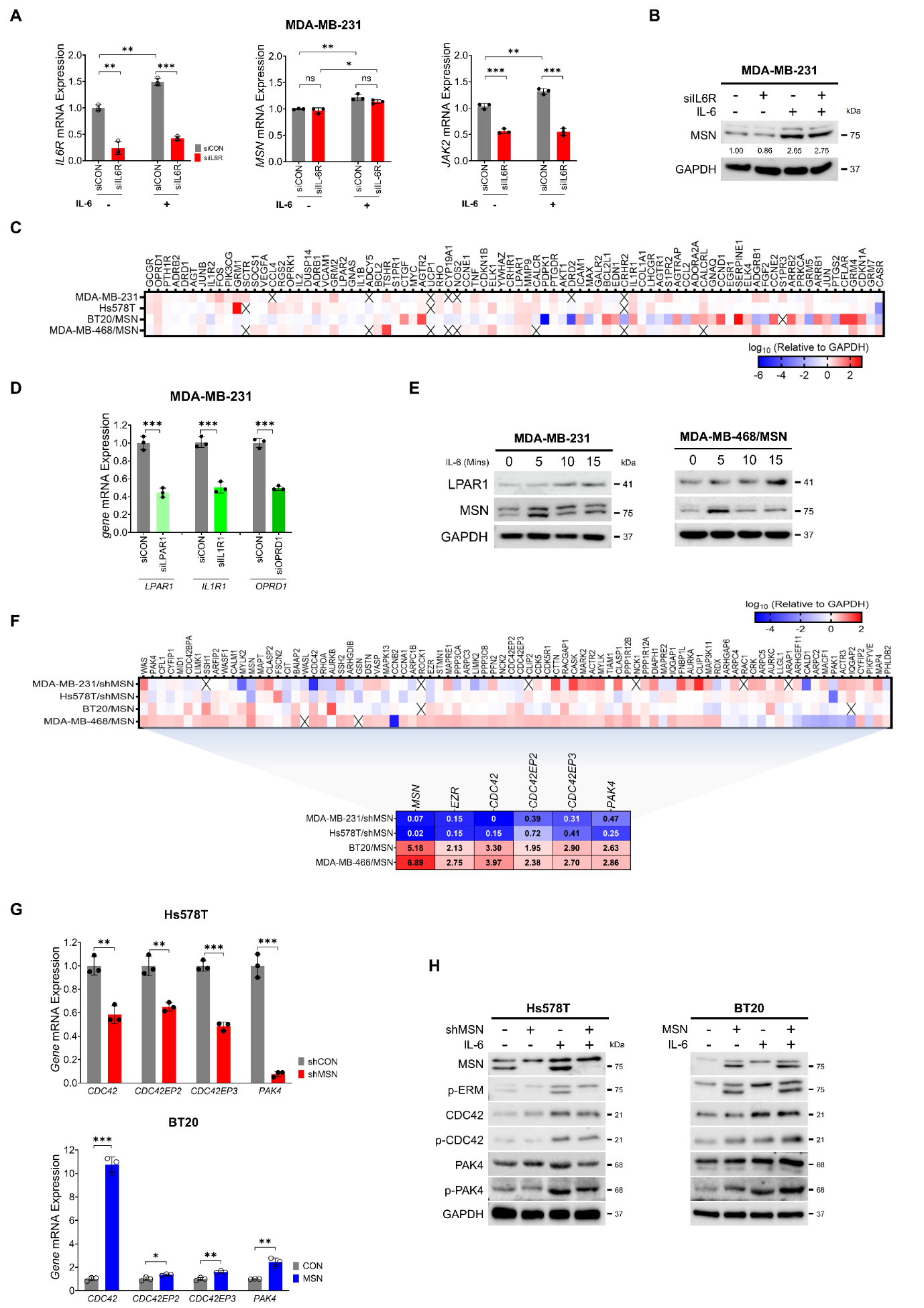


**Supplementary Figure 4. IL-6 Interaction with LPAR1 Activates the MSN-Driven CDC42-PAK4-NF-κB Signaling Cascade. A** Relative mRNA expression of IL6R, MSN, and JAK2 in MDA-MB-231 cells following LPAR1 knockdown, with or without IL-6 treatment, analyzed by RT-qPCR. Statistical analysis: t-tests. **B** Western blot of MSN expression in MDA-MB-231 cells after LPAR1 knockdown with or without IL-6 treatment. **C** **Heatmap of GPCR-related genes (***n* = 84**) from the qPCR panel results, showing** upregulated following IL-6 treatment in MSN-expressing cell lines. **D** Inhibition of relative LPAR1, IL1R1, and OPRD1 mRNA expression in MDA-MB-231 cells after transfection with the indicated siRNAs. Statistical analysis: t-tests. **E** Time-dependent Western blot analysis of LPAR1 and MSN protein levels in MDA-MB-231 and MDA-MB-468/MSN cells following IL-6 treatment. Experiments were performed in duplicate or triplicate (*n* = 2 or 3). **F** Heatmap **of** Rho family GTPase-related genes **(***n* = 84**) from the qPCR panel results,** showing upregulated MSN-overexpressing and downregulated in shMSN cell lines, respectively. Six common genes were identified. **G** Relative mRNA expression levels of the indicated genes in shMSN or MSN-overexpressing cell lines, measured by RT-qPCR. Experiments were performed in triplicate (*n* = 3), and statistical significance between groups was determined using unpaired two-tailed *t*-tests. **H** Western blot analysis of the indicated genes in shMSN or MSN-overexpressing cell lines, with or without IL-6 treatment, compared to their respective control cell lines. *P < 0.05, **P < 0.01, ***P < 0.001.


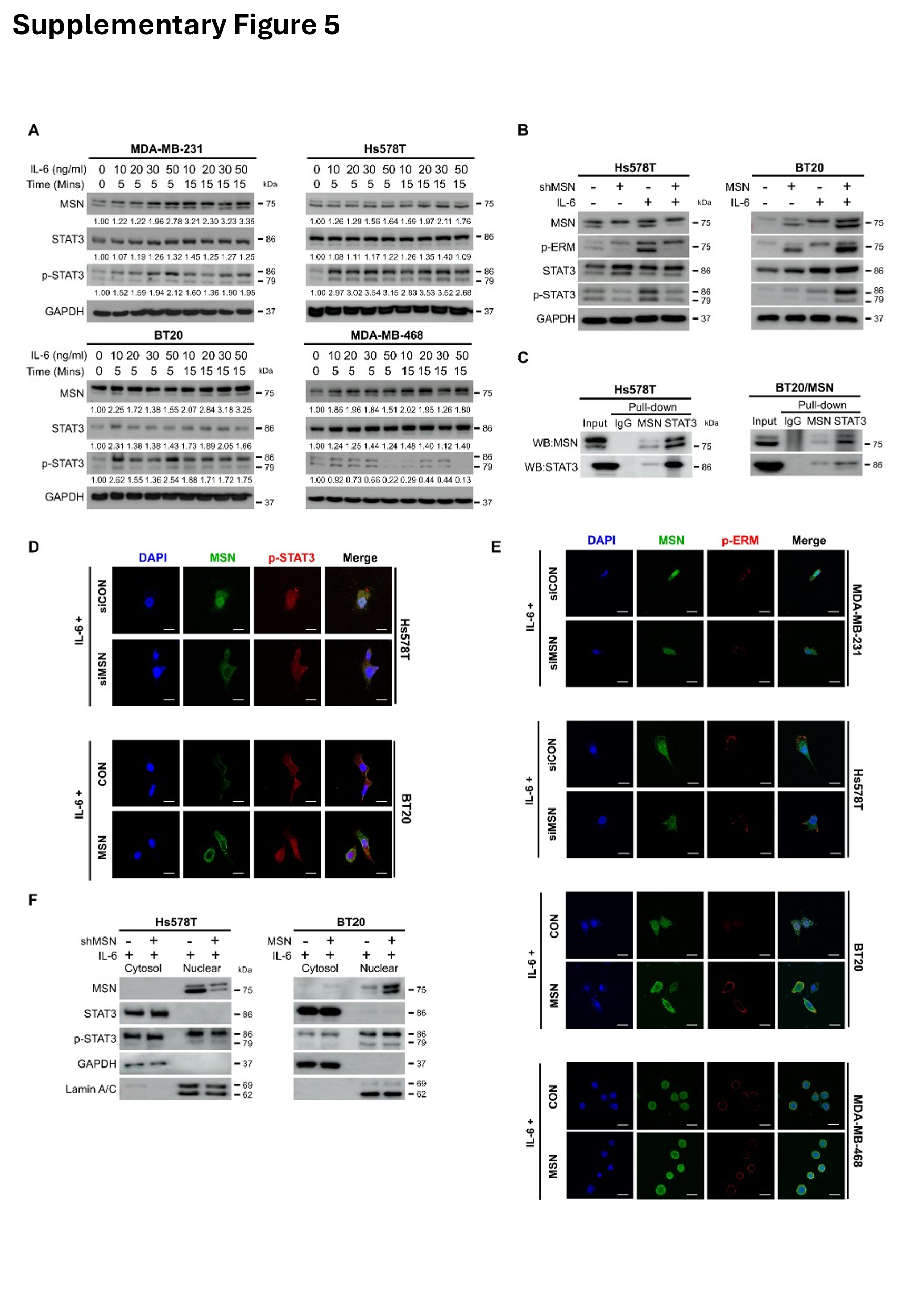


**
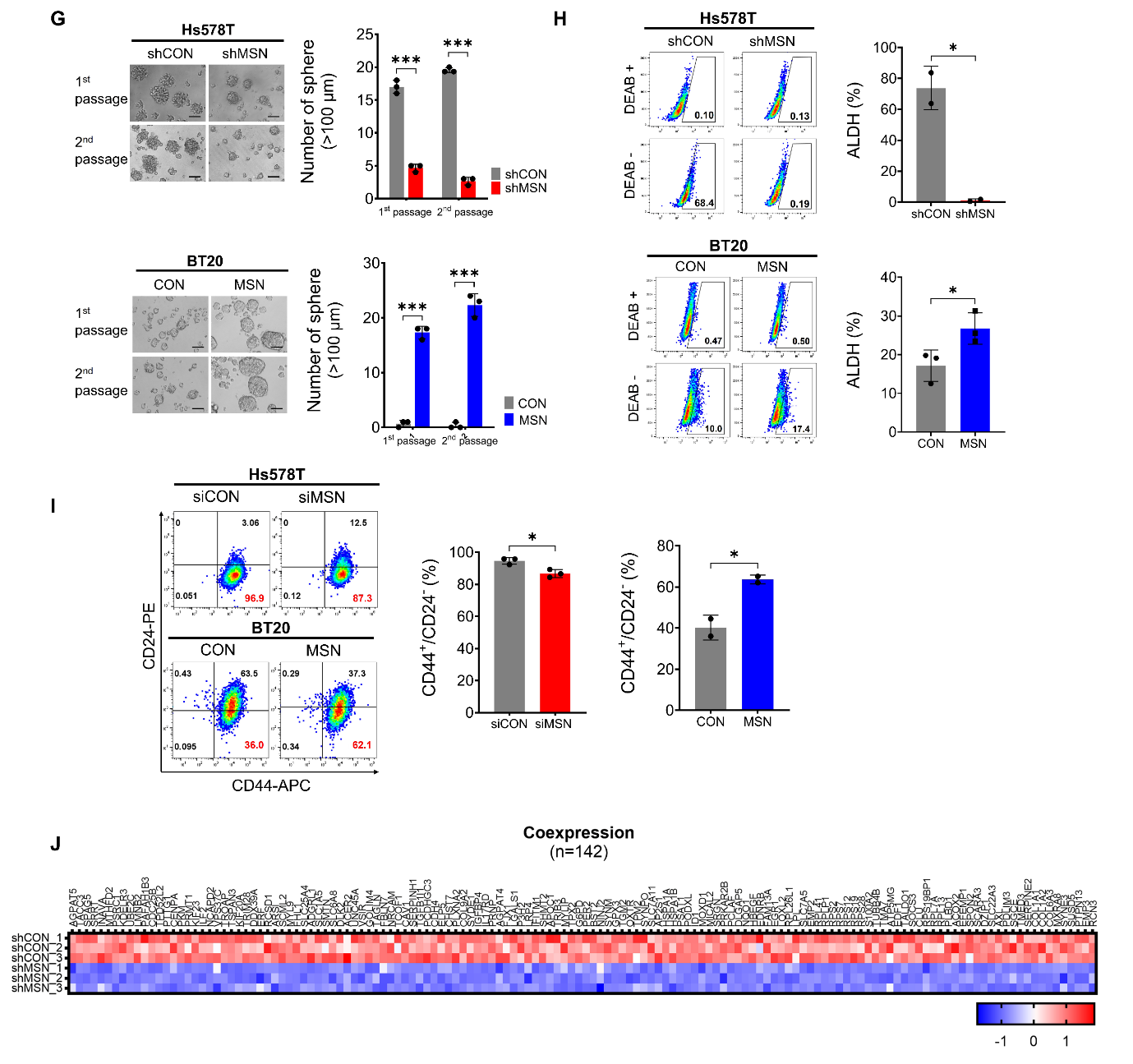

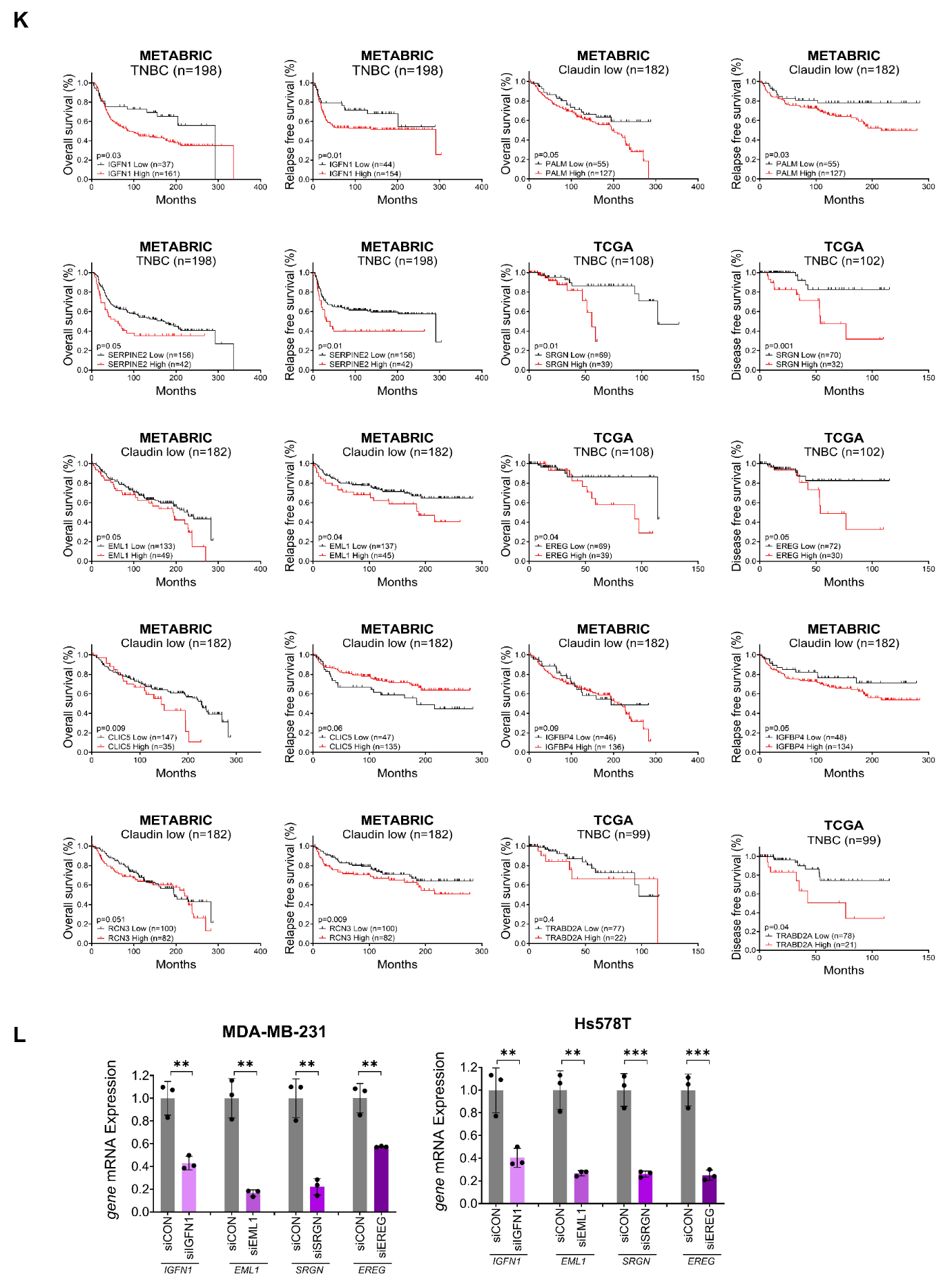
**

**Supplementary Figure 5. MSN Activates STAT3 and Promotes CSC-Related Gene Expression. A** Western blot analysis to determine optimal IL-6 concentration and treatment duration for assessing target expression in each cell line. **B** Western blot of indicated targets in shMSN and MSN-overexpressing cells, with or without IL-6 treatment (30 ng/ml for 15 minutes and 50 ng/ml for 15 minutes), compared to their respective controls. **C** Immunoprecipitation of MSN and STAT3 in Hs578T and BT20/MSN cells using anti-MSN, anti-STAT3, and anti-IgG (negative control). Western blot confirmed binding. **D** Confocal microscopy images of MSN (green), p-STAT3 (red), and DAPI (blue) in Hs578T cells transfected with siMSN or siCON, and in BT20/CON and BT20/MSN cells after IL-6 treatment. Merged images show co-localization. Nuclear co-localization of p-STAT3 and MSN was quantified using ImageJ by measuring overlap with DAPI staining. Bar graphs represent average nuclear co-localization intensity from two independent experiments. Statistical analysis was performed using *t*-tests. Scale bar: 10 μm. **E** Confocal microscopy images of MSN (green), p-ERM (red), and DAPI (blue) in MDA-MB-231 and Hs578T cells transfected with siMSN or siCON, and in MDA-MB-468/CON, BT20/CON, and BT20/MSN cells after IL-6 treatment. Scale bar: 10 μm. **F** Western blot of cytosolic and nuclear fractions from shMSN and MSN-overexpressing cell lines, along with their respective controls. GAPDH and Lamin A/C were used as loading controls for cytosolic and nuclear fractions, respectively. **G** Representative day 7 tumorsphere images from first and second sphere formation assays, quantifying spheres >100 μm. Experiments were performed in triplicate (*n* = 3), and statistical significance was assessed using unpaired two-tailed *t*-tests versus control. **H** ALDH activity in Hs578T/shCON, Hs578T/shMSN, BT20/CON, and BT20/MSN cells. Bar graphs show ALDH results, with DEAB-treated cells as negative controls. Experiments were performed in duplicate (*n* = 2), and statistical significance was assessed using unpaired two-tailed *t*-tests. **I** CD44^+^/CD24^−^ population percentage in Hs578T/shCON, Hs578T/shMSN, BT20/CON, and BT20/MSN cells, quantified by flow cytometry. Experiments were performed in triplicate (*n* = 3), and statistical significance was determined using unpaired two-tailed *t*-tests. **J** Heatmap of 142 stem cell-related genes identified in MDA-MB-231/shMSN cells vs. control from the COEXPRESSION dataset (ToppGene). **K** OS and DFS analysis of the top 10 genes (METABRIC and TCGA datasets) from 33 common genes identified between 1,301 downregulated DEGs in MDA-MB-231/shMSN and 367 upregulated stemness genes from BCSCdb. **L** RT-qPCR of four selected CSC-related genes in MDA-MB-231 and Hs578T cells, comparing siRNA-transfected vs. siCON. Experiments were performed in triplicate (*n* = 3), and statistical significance was assessed using unpaired two-tailed *t*-tests. *P < 0.05, **P < 0.01, ***P < 0.001.


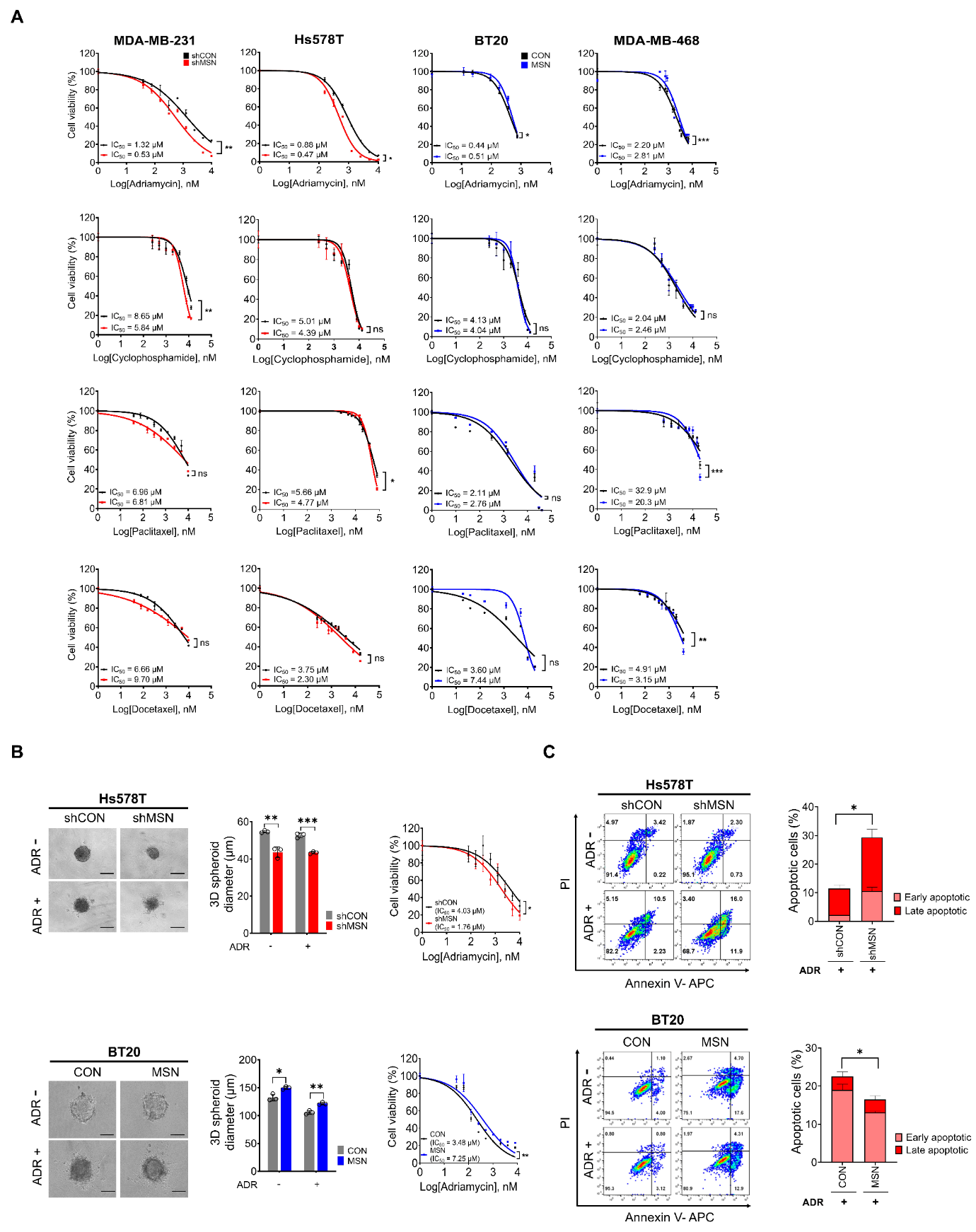


**
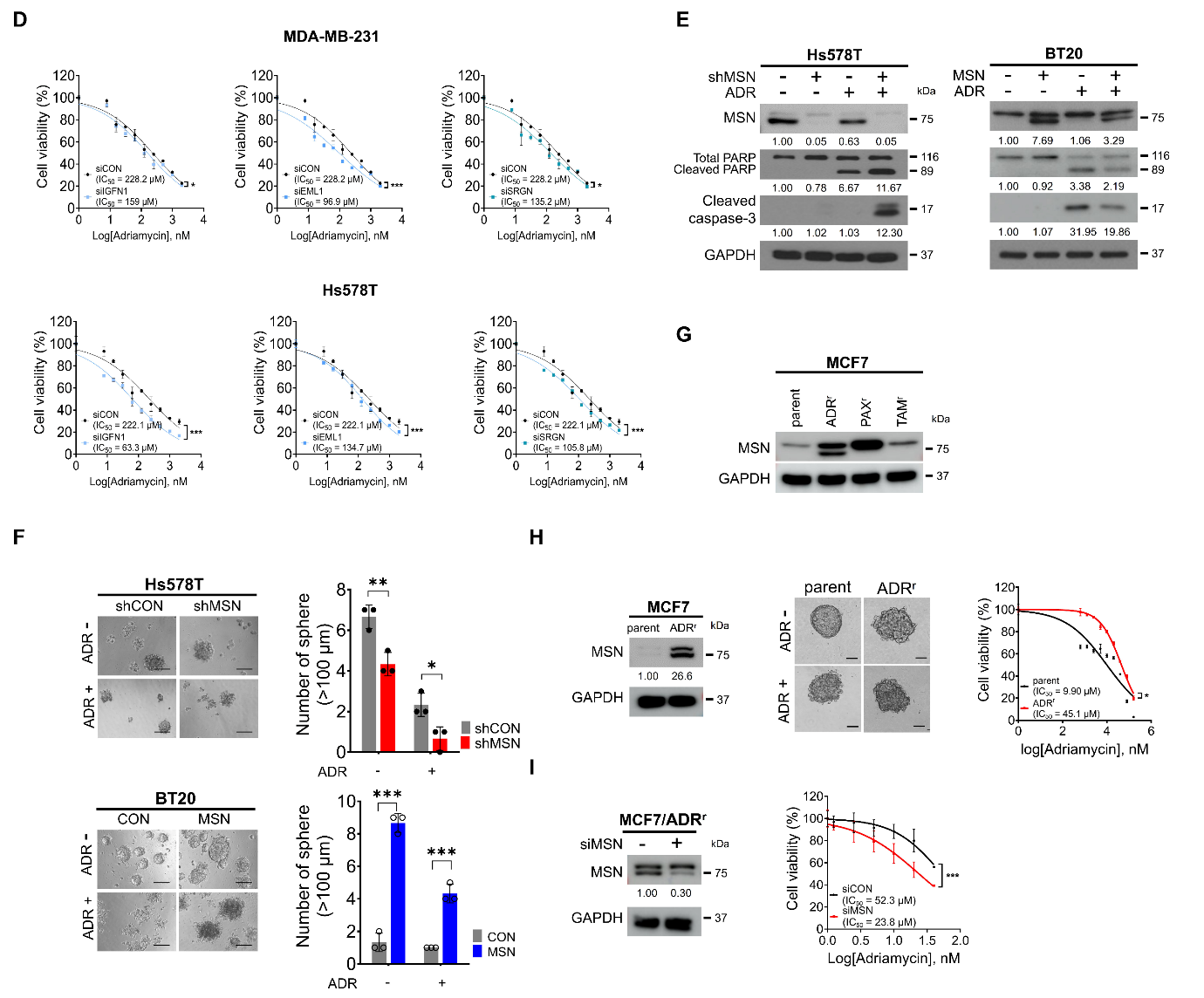
****Supplementary Figure 6. MSN-Induced Chemoresistance to Adriamycin in TNBC Cells. A** Cell viability of shMSN and MSN-overexpressing cells, along with their respective control cells, after treatment with Adriamycin, cyclophosphamide, paclitaxel, and docetaxel. IC50 values were calculated. Experiments were performed in triplicate (*n* = 3), and statistical significance was assessed using dose-dependent mixed regression analysis. **B** 3D spheroid culture of Hs578T/shMSN and BT20/MSN cell lines, with or without ADR treatment. Spheroid diameters were measured, and results were presented as mean ± SD. Experiments were performed in triplicate (*n* = 3), and statistical analysis was performed using unpaired two-tailed *t*-tests. **C** Apoptosis analysis of shMSN and MSN-overexpressing cells, with or without ADR, by PI and Annexin V staining. Apoptotic percentages were recorded. Experiments were performed in duplicate (*n* = 2), and statistical significance was determined using unpaired two-tailed *t*-tests. **D** Cell viability and IC50 values in MDA-MB-231 and Hs578T cell lines after ADR treatment and siRNA-mediated inhibition of three CSC-related genes. Experiments were performed in triplicate (*n* = 3), and statistical analysis was performed using dose-dependent mixed regression analysis. **E** Western blot of indicated targets in shMSN and MSN-overexpressing cells, with or without ADR (2 µM and 4 µM for 24 hours). **F** Tumorsphere images (day 7) from sphere formation assays in shMSN and MSN-overexpressing cells, with or without ADR, and quantifying spheres >100 μm. Experiments were performed in triplicate (*n* = 3), and statistical analysis was performed using unpaired two-tailed *t*-tests. **G** Western blot of MSN in MCF7 parental cells and ADR^r^, PTX^r^, and TAM^r^, using GAPDH as a loading control. **H** Western blot, 3D spheroid images, and ADR response in MCF7 and ADR-resistant cells. Cell viability and IC50 values were calculated. Experiments were performed in triplicate (*n* = 3), and statistical significance was determined using mixed regression analysis. **I** Western blot of MSN in siMSN-transfected vs. siCON-transfected ADR-resistant cells. IC50 values were calculated post-ADR treatment. Statistical analysis: mixed regression. Statistical analysis was performed using mixed regression. *P < 0.05, **P < 0.01, ***P < 0.001.

**
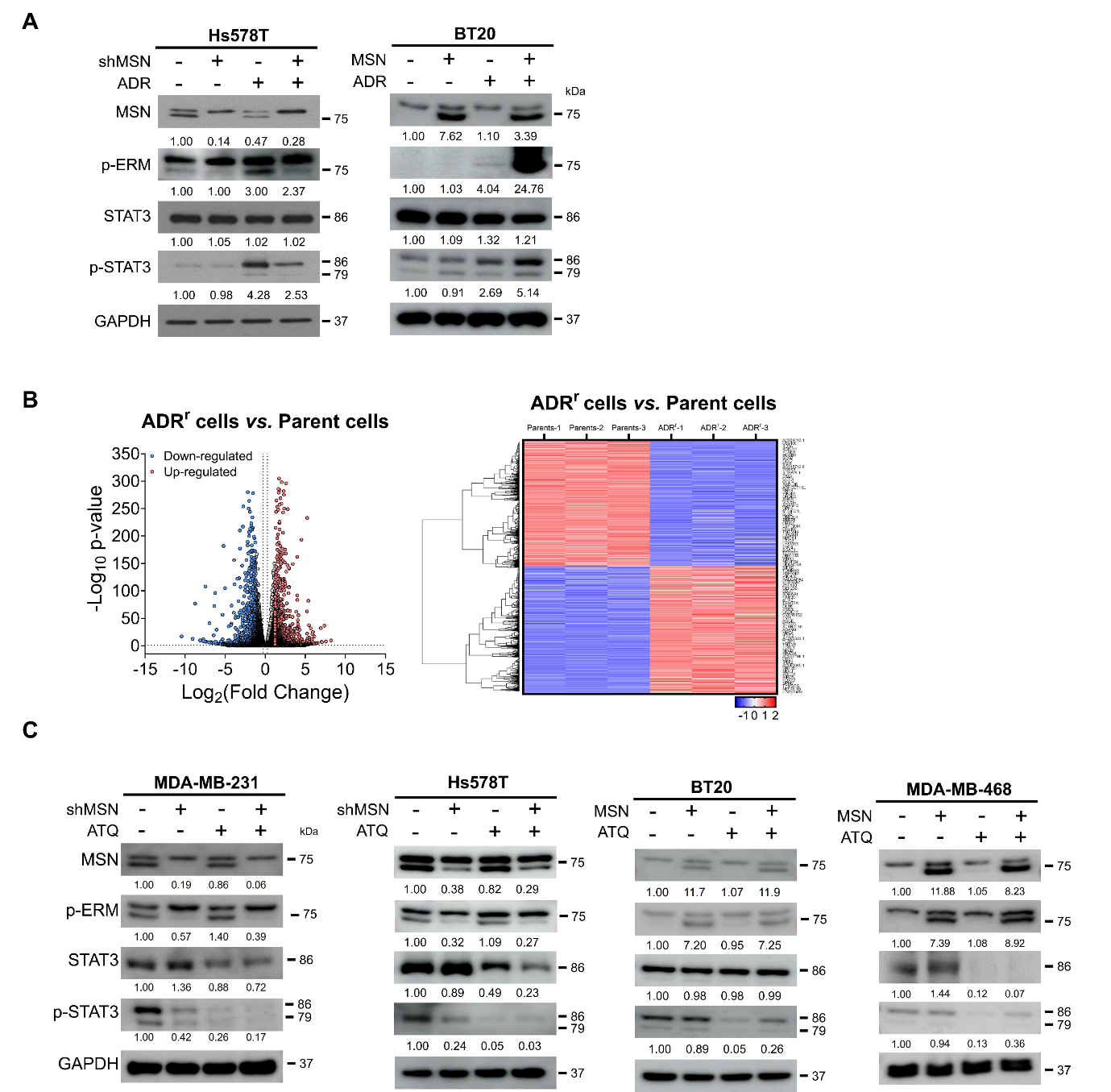
****Supplementary Figure 7. Characteristics of ADR-resistant MDA-MB-231 cells. A** Western blot of indicated targets in shMSN and MSN-overexpressing cells, with or without ADR (2 µM and 4 µM for 24 hours). **B** Volcano RNA-seq plot comparing ADR-resistant (ADR^r^) gene expression profiles vs. MDA-MB-231 parental cells. Significantly upregulated and downregulated genes are highlighted. Heatmap of RNA-seq showing differentially expressed genes between MDA-MB-231 parental cells and ADR-resistant cells. Genes with *p*-values ≤ 0.05 and │Log2(1.2)│ considered significant. Hierarchical clustering applied. **C** Western blot analysis of the indicated target in shMSN and MSN-overexpressing cell lines, treated with or without ATQ, compared to their control cell lines.

**Supplementary Table S1. List of siRNAs used in this study**

| **Gene** | **Forward (5′- 3′)** | **Reverse (5′- 3′)** |
| --- | --- | --- |
| *MSN* | CAGAUCGAGGAACAGACUA | UAGUCUGUUCCUCGAUCUG |
| *EZR* | GAUUCACACAUGCCACUAU | AUAGUGGCAUGUGUGAAUC |
| *RDX* | CAGGCCGUGAUAAGUACAA | UGUACUUAUCACGGCCUG |
| *STAT3* | UGUUCUCUGAGACCCAUGA | UCAUGGGUCUCAGAGAACA |
| *JAK2* | GUCAGUAUUAAGCAAGCAA | UUGCUUGCUUAAUACUGAC |
| *NF-κB (p65)* | GGUGUAUUUCACGGGACCA | UGGUCCCGUGAAAUACACC |
| *CDC42* | GAUUACGACCGCUGAGUUA | UAACUCAGCGGUCGUAAUC |
| *PAK4* | CUAAGAGGUGAACAUGUAU | AUACAUGUUCACCUCUUAG |
| *LPAR1* | CGUUUAUGCCUAUCAGCAU | AUGCUGAUAGGCAUAAACG |
| *IL1R1* | CAGGAUUCAUCAACACAAA | UUUGUGUUGAUGAAUCCUG |
| *ORPD1* | GGGCUUCAACCUUGAGACA | UGUCUCAAGGUUGAAGCCC |
| *IGFN1* | GGUCAGGUUAUAGGGAGGA | UCCUCCCUAUAACCUGACC |
| *EML1* | GUGCUAAGCAGACUCUACU | AGUAGAGUCUGCUUAGCAC |
| *SRGN* | GUAUGAUUGCUGUUACAUA | UAUGUAACAGCAAUCAUAC |
| *EREG* | CUCCAAUAUCCAUUCUGUA | UACAGAAUGGAUAUUGGAG |
| *IL6R* | UCAGCAAAACUCAAACCUU | AAGGUUUGAGUUUUGCUGA |

**Supplementary Table S2.** **Chemotherapeutic response analysis based on MSN expression in TNBC patients who received neoadjuvant chemotherapy (SHUH cohort)**

| **Pathological types** | **No. (%)** | **MSN Low** | **MSN High** |
| --- | --- | --- | --- |
| **Complete remission (CR)** |  |  |  |
| Ductal carcinoma in situ (DCIS) | 4 (4.65%) | 2 (2.33%) | 2 (2.33%) |
| No residual tumor | 42 (48.84%) | 27 (31.40%) | 15 (17.44%) |
| **Non-Complete remission (nCR)** |  |  |  |
| Microinvasive carcinoma | 1 (1.16%) | 1 (1.16%) | 0 (0%) |
| Infiltrating duct carcinoma | 24 (27.91%) | 13 (15.12%) | 11 (12.79%) |
| Invasive ductal carcinoma (IDC) | 13 (15.12%) | 3 (3.49%) | 10 (11.63%) |
| Metaplastic carcinoma | 2 (2.33%) | 0 (0%) | 2 (2.33%) |

**Supplementary Table S3. List of antibodies used for western blot in this study**

| **Antigen** | **Clone** | **Type** | **Dilution** |  | **RRID** | **Vendor** |
| --- | --- | --- | --- | --- | --- | --- |
| MSN | 38/87 | Monoclonal | 1:1000 |  | AB_784475 | Santa Cruz |
| p-ERM |  | Polyclonal | 1:500 |  | AB_330232 | Cell Signaling |
| STAT3 | 124H6 | Monoclonal | 1:2000 |  | AB_331757 | Cell Signaling |
| p-STAT3 | D3A7 | Monoclonal | 1:1000 |  | AB_2491009 | Cell Signaling |
| NF-κB (p65) | D14E12 | Monoclonal | 1:2000 |  | AB_10859369 | Cell Signaling |
| p-NF-κB (p65) | 93H1 | Monoclonal | 1:1000 |  | AB_331284 | Cell Signaling |
| CDC42 | 11A11 | Monoclonal | 1:500 |  | AB_2078082 | Cell Signaling |
| p-CDC42 |  | Polyclonal | 1:500 |  | AB_2300703 | Cell Signaling |
| PAK4 | B-3 | Monoclonal | 1:500 |  |  | Santa Cruz |
| p-PAK4 | 93 | Monoclonal | 1:500 |  | AB_2158453 | Santa Cruz |
| LPAR1 | B-10 | Monoclonal | 1:500 |  | AB_2687441 | Santa Cruz |
| IL-6 | D3K2N | Monoclonal | 1:500 |  | AB_2687897 | Cell Signaling |
| Cleaved Caspase 3 | 5A1E | Monoclonal | 1:500 |  | AB_2070042 | Cell Signaling |
| PARP |  | Polyclonal | 1:1000 |  | AB_2160739 | Cell Signaling |
| GAPDH | 6C5 | Monoclonal | 1:4000 |  | AB_627679 | Santa Cruz |

**Supplementary Table S4. List of primers used for RT-qPCR in this study**

| **Gene** | **Forward (5′- 3′)** | **Reverse (5′- 3′)** |
| --- | --- | --- |
| *MSN* | CCTAGGTGGTGTGATTCCCA | ACACTCAGAAGCAGTCCTGT |
| *EZR* | ATCCAGGACATCACCCAGAAA | GTACCCAGACTTGTGCACTTC |
| *RDX* | TGCCGAAACCAATCAACGTAA | TGTCTACATACTGCAGCCCAA |
| *JAK2* | TTTGCTGTCGAGCGAGAAAAT | TGAGCGAACAGTTTCCATCTG |
| *CDC42* | TAGGCCTGTGAGTTGGGAAG | GTATCCCTGACCGTTTGCA |
| *PAK4* | CGAGGTGGTAATCATGAGGGA | GATCTGCTCCTCGTTCATCCT |
| *CDC42EP2* | ATCTATCTGAAGCGTGGCAGT | GAGATGTCGCCAAACATGTCA |
| *CDC42EP3* | AGGATCCCAAGCTCTCATGTT | TGTCCCATTCTCCAACAGACT |
| *LPAR1* | TATCATCTGCTGGACTCCTGG | TCTTTGTCGCGGTAGGAGTAA |
| *IL1R1* | GCTAAGGTGGAGGATTCAGGA | GCAACGGGTAGTTTCTGCTTA |
| *ORPD1* | ACATCTGTATCTGGGTCCTGG | GAACACGCAGATCTTGGTCA |
| *IGFN1* | GGTCCCCTTGGTCATGGTAA | TGGACAAGCAGGGAGAGAAG |
| *EML1* | AACATCAAGAGGACCAGCTCT | CTCCTTGGGTTTGCTTTCACT |
| *SRGN* | GATCTGGGAGTGGCTTCCTAA | ATCCATGTTGACCCAAGTCCT |
| *EREG* | TCCTCGTGTCAAATTCCGGA | CATTGATGTCGGTTCCGTCC |
| *IL6R* | CAAACCTTTCAGGGTTGTGGA | TGAGCTCAAACCGTAGTCTGT |
| *GAPDH* | GAAGGTGAAGGTCGGAGT | GAAGATGGTGATGGGATTTC |

**Supplementary Table S5. List of primers used for ChIP assay in this study**

| **Gene** | **Sequence (5’- 3’)** | **Location** | **Accession** |
| --- | --- | --- | --- |
| *IGFN1* | (F) GGAGTCAAAGAGGAGGCAGA  (R) TGGAGTACAGTGACGCGATCT | -863 nt ~ -656 nt | NM_001164586.2 |
| *EML1* | (F) CCTACTTGCCATCACCCAGA  (R) TTAATGGCCACGAGGGAAGT | -19802 nt ~ -19610 nt | NM_001008707.2 |
| *PALM* | (F) CATCTGTGGCCTGCTTTGAC  (R) AGCTTCCCCTTTCCCTTCTC | -7533 nt ~ -7350 nt | NM_002579.3 |
| *SERPINE2* | (F) TGGCAGGAAAAGCTGTTGAG  (R) TCCCCGGATTTATCTCAGCC | -7444 nt ~ -7261 nt | NM_006216.4 |
| *SRGN* | (F) ACAAACTGTCCCAAATGCTGA  (R) TCCAAAACCTCAAACCCTGC | -315 nt ~ -109 nt | NM_002727.4 |
| *EREG* | (F) TCAGAGGGACACAGCCAAC  (R) GAGCATCTCCATCCTCCTCC | -124 nt ~ 9 nt | NM_001432.3 |

| **Supplementary Table S6. Clinicopathologic characteristics of TNBC patients based on MSN expression** | | | |
| --- | --- | --- | --- |
| **SHUH cohort** | **MSN Low**  **N (%)** | **MSN High**  **N (%)** | ***p*-value** |
| **Pathological parameter** |  |  |  |
| Age(yr) |  |  | 0.61 |
| <50 years | 76/181 (42.0%) | 19/181 (10.5%) |  |
| ≥50 years | 71/181 (39.2%) | 15/181 (8.3%) |  |
| Histologic Grade |  |  | **0.03** |
| grade 1 | 0/170 (0%) | 0/170 (0%) |  |
| grade 2 | 32/170 (18.8%) | 2/170 (1.2%) |  |
| grade 3 | 105/170 (61.8%) | 31/170 (18.2%) |  |
| Lymphatic invasion |  |  | 0.90 |
| Negative | 87/179 (48.6%) | 20/179 (11.2%) |  |
| Positive | 58/179 (32.4%) | 14/179 (7.8%) |  |
| Lymph nodes metastasis |  |  | 0.88 |
| Negative | 97/181 (53.6%) | 22/181 (12.2%) |  |
| Positive | 50/181 (27.8%) | 12/181 (6.7%) |  |
| Nuclear Grade |  |  | **0.04** |
| N1 | 0/170 (0%) | 0/170 (0%) |  |
| N2 | 30/170 (17.6%) | 2/170 (1.2%) |  |
| N3 | 107/170 (62.9%) | 31/170 (18.2%) |  |
| Stage |  |  | 0.83 |
| stage 0 | 3/181 (1.7%) | 0/181 (0%) |  |
| stage 1 | 35/181 (19.3%) | 8/181 (4.4%) |  |
| stage 2 | 84/181 (46.4%) | 21/181 (11.6%) |  |
| stage 3 | 25/181 (13.8%) | 5/181 (2.8%) |  |
| T Stage |  |  | 0.85 |
| T1 | 47/178 (26.4%) | 11/178 (6.2%) |  |
| T2 | 89/178 (50.0%) | 22/178 (12.4%) |  |
| T3 | 7/178 (3.9%) | 1/178 (0.6%) |  |
| T4 | 1/178 (0.6%) | 0/178 (0%) |  |
| Vascular invasion |  |  | 0.40 |
| Negative | 142/179 (79.3%) | 34/179 (19.0%) |  |
| Positive | 3/179 (1.7%) | 0/179 (0%) |  |
|  | | | |

**List of Supplementary Table**

**Supplementary Table S1.** List of siRNAs used in this study

**Supplementary Table S2.** Chemotherapeutic response analysis based on MSN expression in TNBC patients who received neoadjuvant chemotherapy (SHUH cohort)

**Supplementary Table S3.** List of antibodies used for western blot in this study

**Supplementary Table S4.** List of primers used for RT-qPCR in this study

**Supplementary Table S5.** List of primers used for ChIP assay in this study

**Supplementary Table S6.** Clinicopathologic characteristics of TNBC patients based on MSN expression

**Supplementary Table S7.** Downregulated gene list from RNA-seq comparing MDA-MB-231/shMSN to control cells

**Supplementary Table S8.** Gene ontology enrichment analysis of downregulated genes comparing MDA-MB-231/shMSN to control cells

**Supplementary Table S9.** qPCR panel results for GPCR-related genes in MSN-expressing cell lines following IL-6 treatment

**Supplementary Table S10.** qPCR panel results for Rho GTPase-related genes in shMSN or MSN-overexpressing cell lines

**Supplementary Table S11.** Analysis of Coexpression dataset from ToppGene and RNA-seq results of shMSN cells

**Supplementary Table S12.** Analysis of upregulated cancer stemness-related genes from BCSCdb and RNA-seq data of shMSN cells

**Supplementary Table S13**. Upregulated gene list in ADR-resistant MDA-MB-231 cells from RNA-seq analysis

**Supplementary Table S14**. Gene ontology enrichment analysis of upregulated genes in ADR-resistant MDA-MB-231 cells

**Supplementary Table S15**. Analysis of Coexpression data from ToppGene and RNA-seq results in ADR-resistant MDA-MB-231 cells
