# Supplementary material for: MSN/STAT3 drives cancer stemness and chemoresistance via IL-6/LPAR1 ligand receptor complex in triple-negative breast cancer
Source: Breast Cancer Res. 2025 Jul 22;27:136. doi: 10.1186/s13058-025-02072-z (PMC12281688; doi:10.1186/s13058-025-02072-z)
Supplement: Supplementary file 14 — Additional file 14. [file 13058_2025_2072_MOESM14_ESM.pptx]

## Slide 1
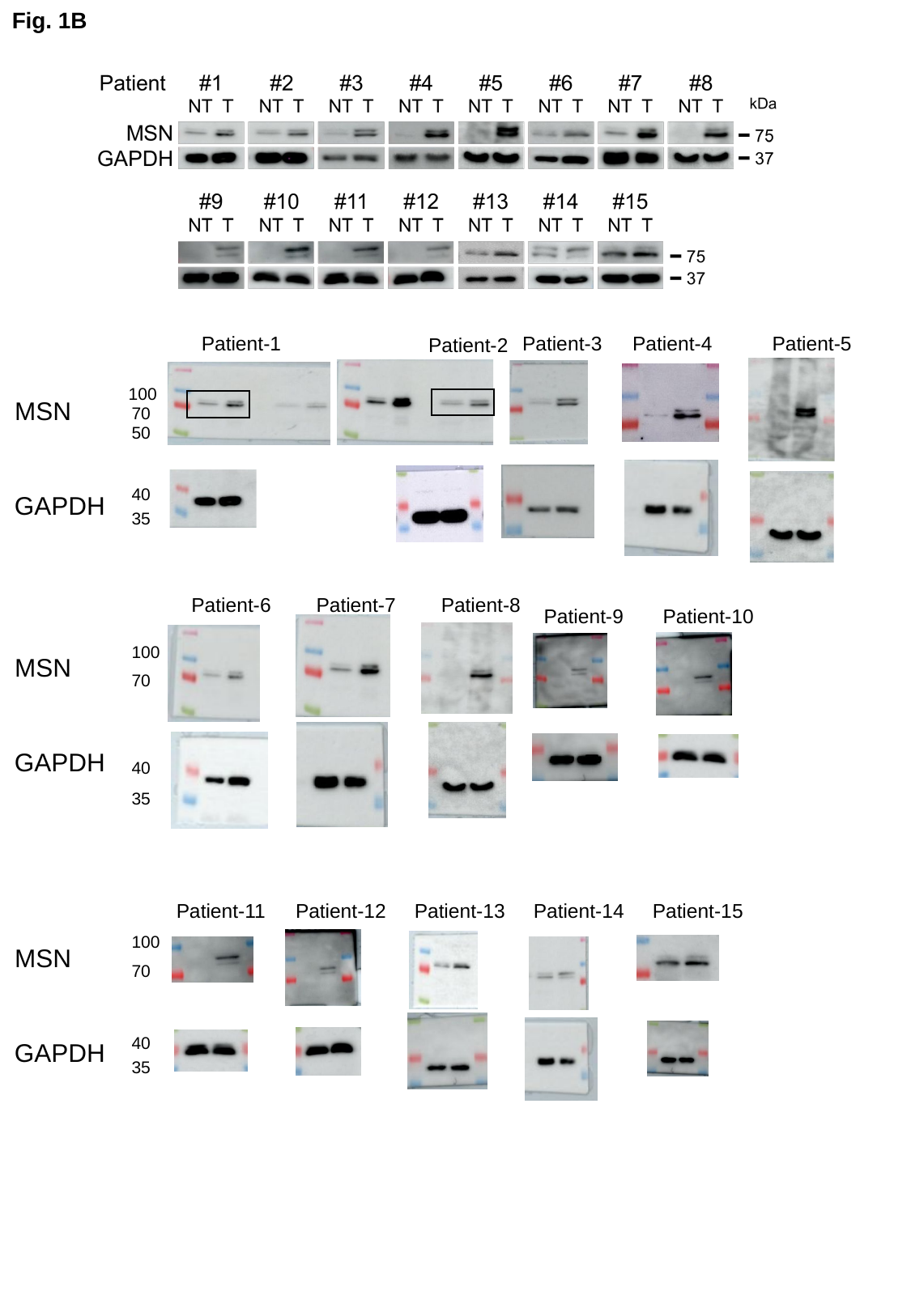

Fig. 1B
Patient-4
Patient-1
Patient-3
Patient-5
Patient-2
100
MSN
70
50
40
GAPDH
35
Patient-6
Patient-7
Patient-8
Patient-9
Patient-10
100
MSN
70
GAPDH
40
35
Patient-11
Patient-12
Patient-13
Patient-14
Patient-15
100
MSN
70
40
GAPDH
35

## Slide 2
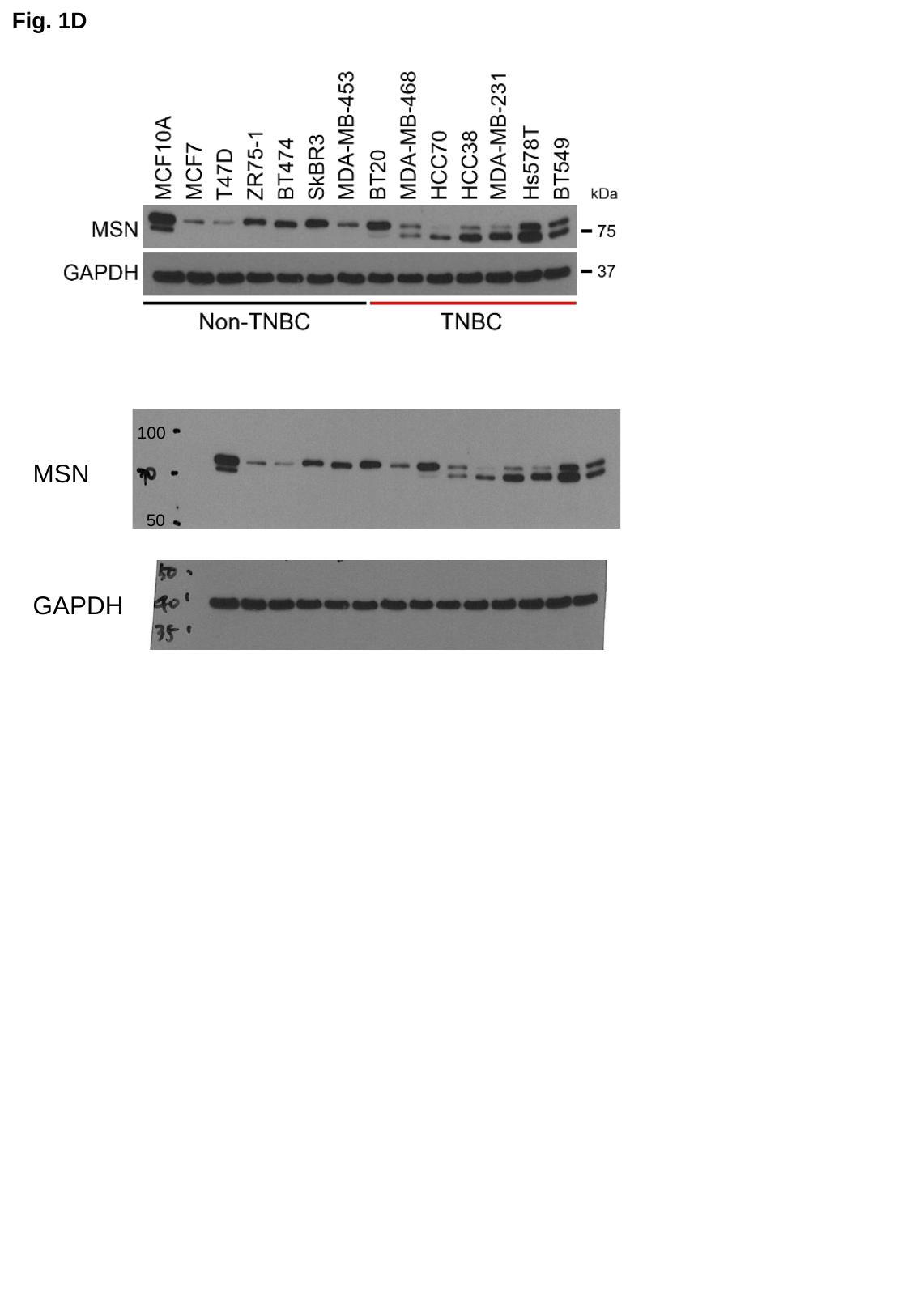

Fig. 1D
100
MSN
50
GAPDH

## Slide 3
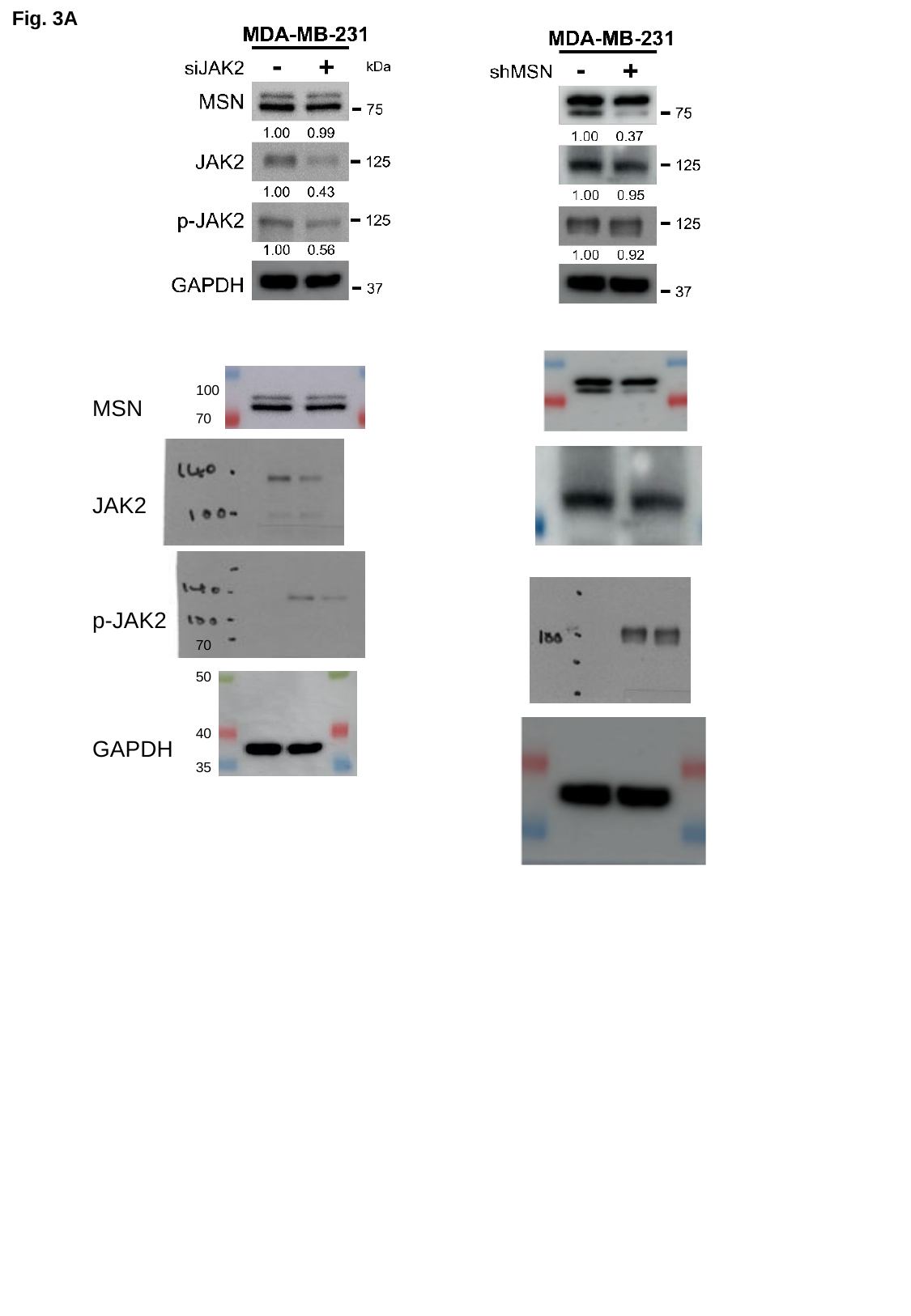

Fig. 3A
100
MSN
70
JAK2
p-JAK2
70
50
40
GAPDH
35

## Slide 4
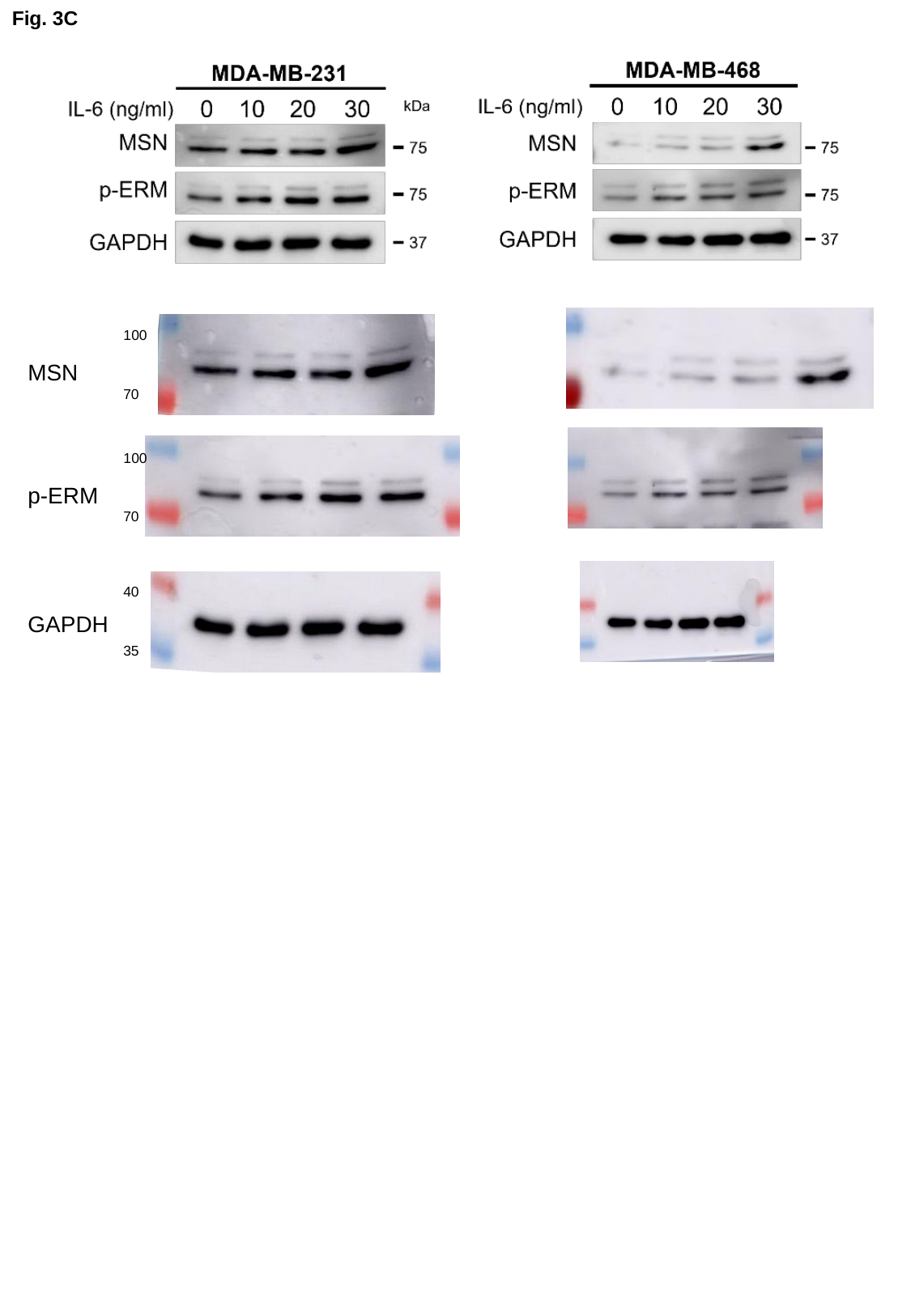

Fig. 3C
100
MSN
70
100
p-ERM
70
40
GAPDH
35

## Slide 5
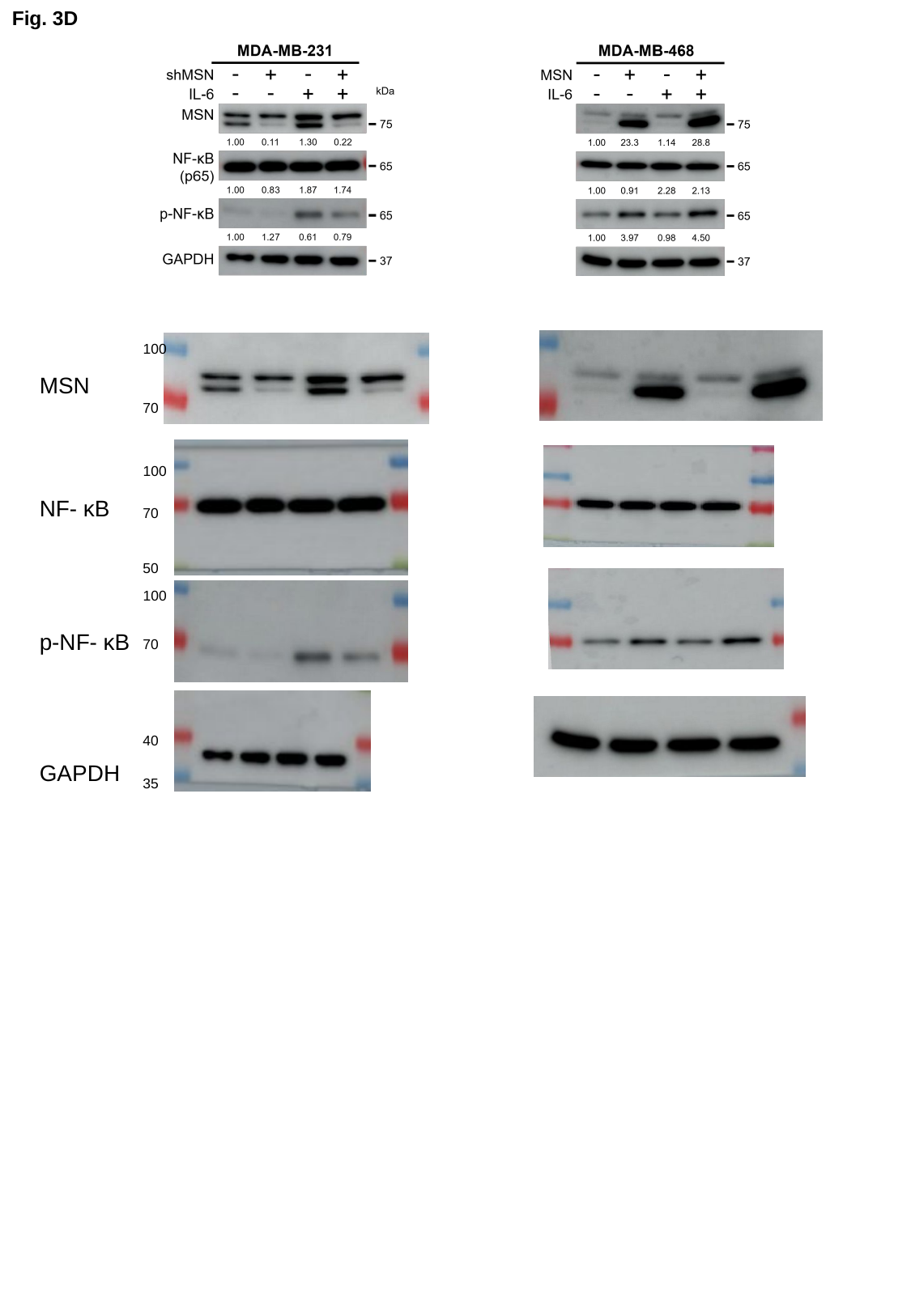

Fig. 3D
100
MSN
70
100
NF- κB
70
50
100
p-NF- κB
70
40
GAPDH
35

## Slide 6
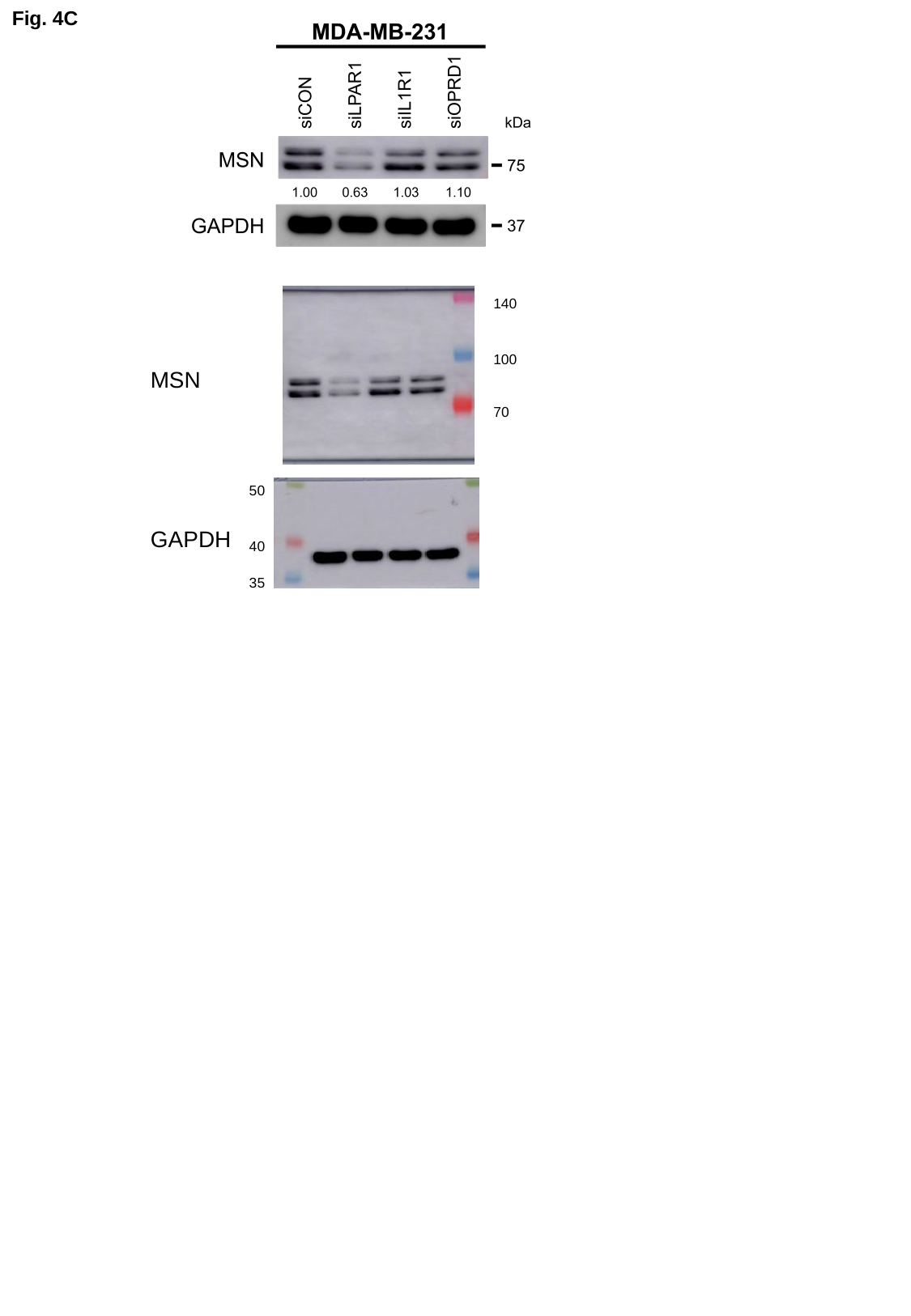

Fig. 4C
140
100
MSN
70
50
GAPDH
40
35

## Slide 7
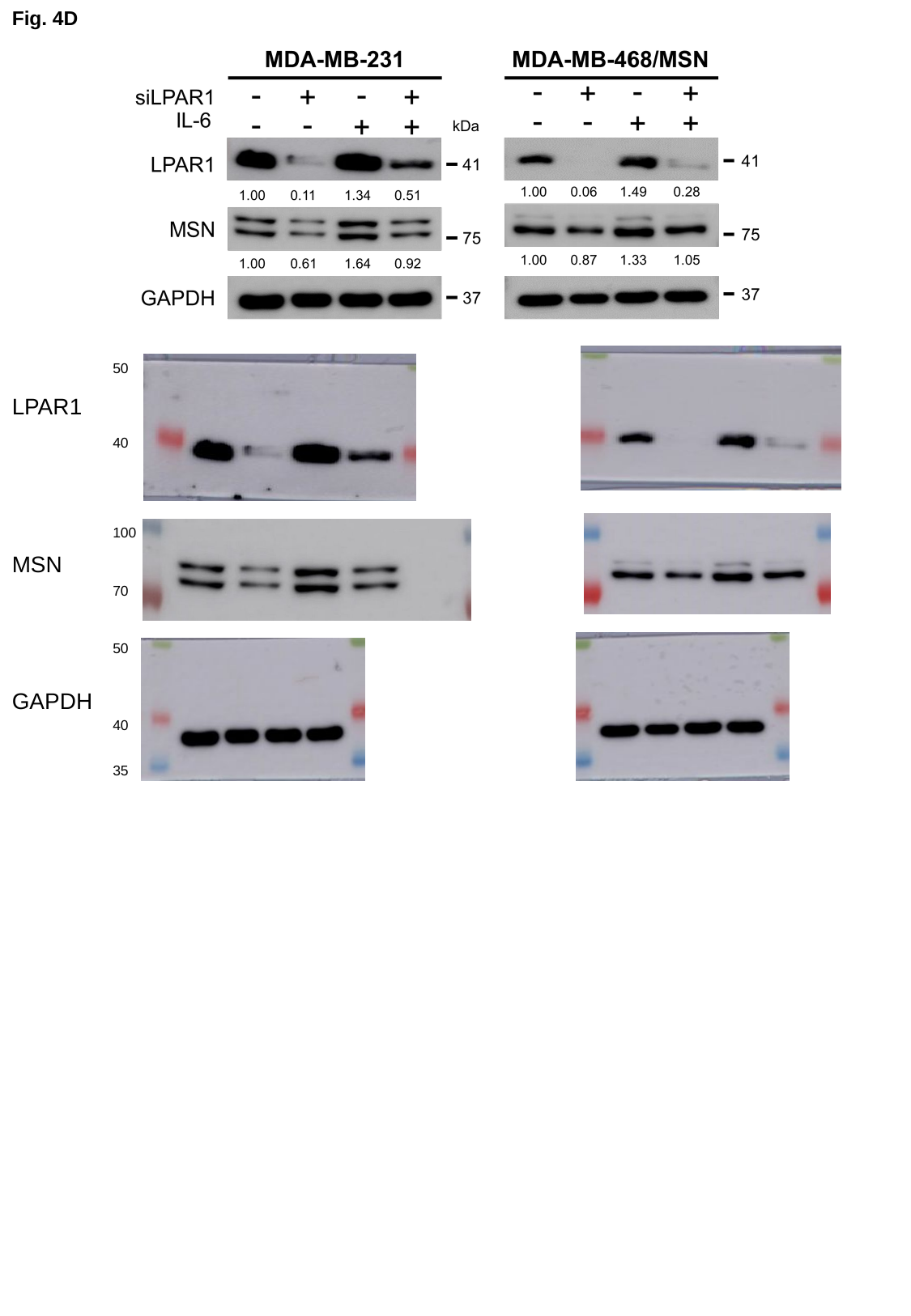

Fig. 4D
50
LPAR1
40
100
MSN
70
50
GAPDH
40
35

## Slide 8
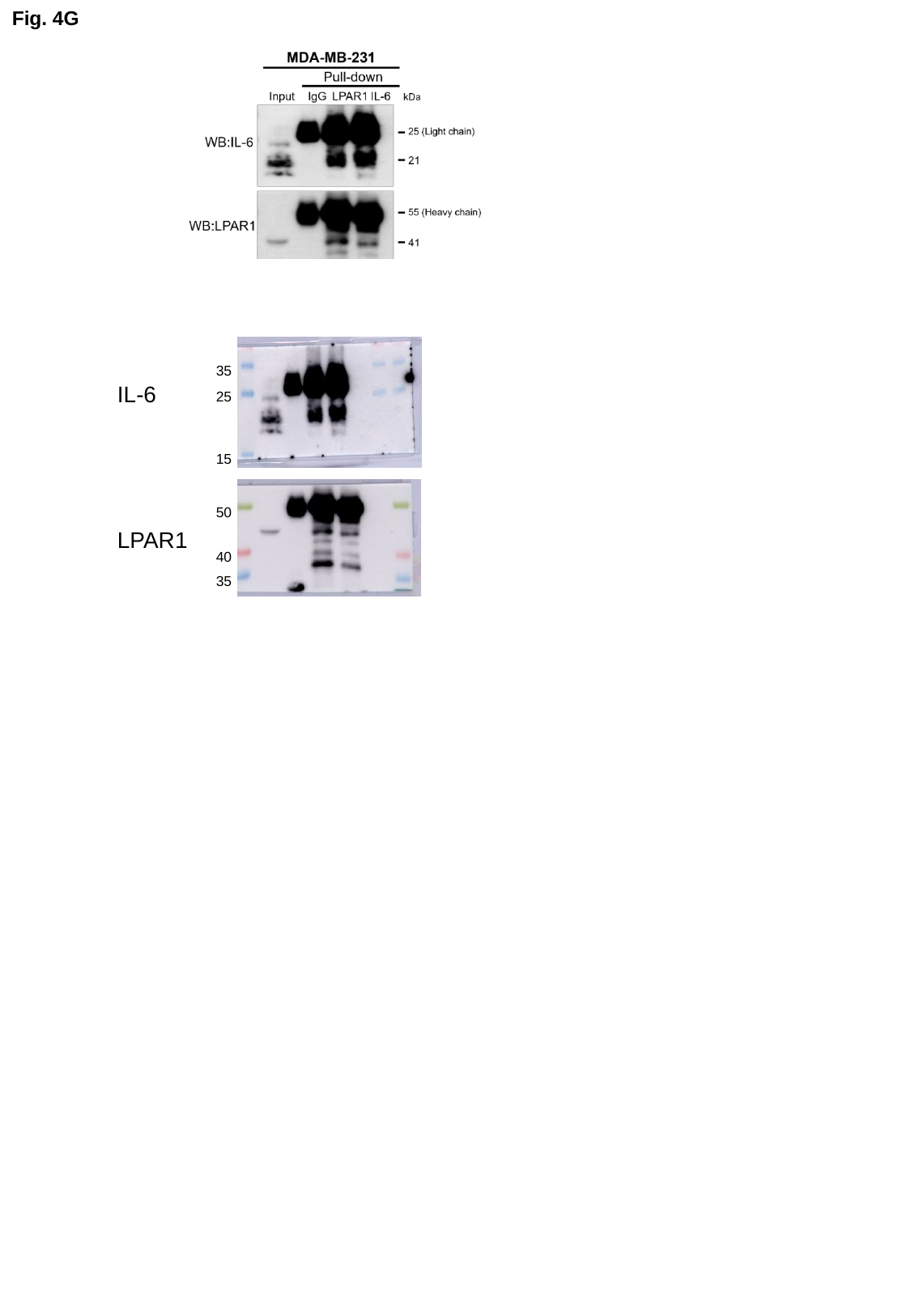

Fig. 4G
35
IL-6
25
15
50
LPAR1
40
35

## Slide 9
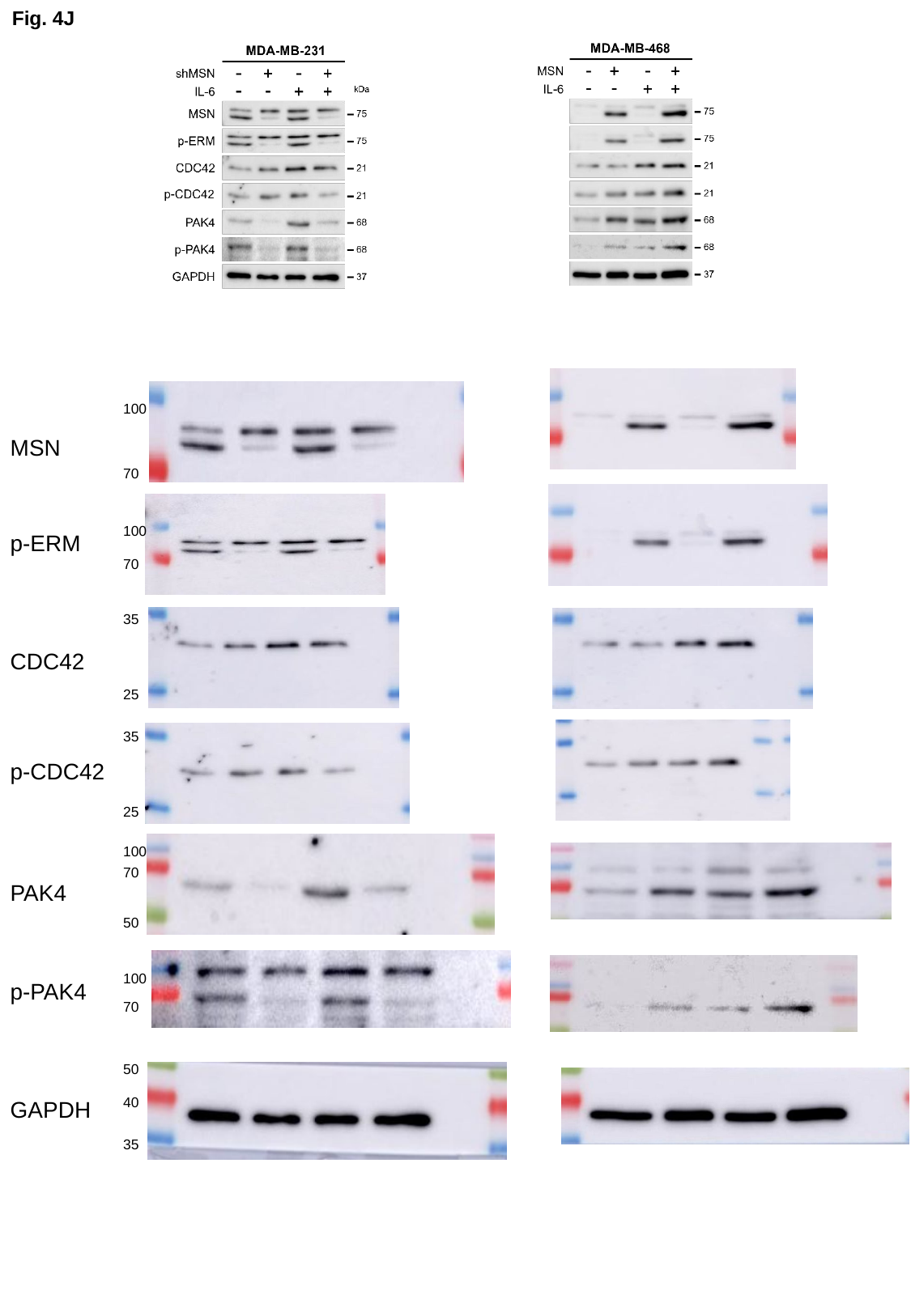

Fig. 4J
100
MSN
70
100
p-ERM
70
35
CDC42
25
35
p-CDC42
25
100
70
PAK4
50
100
p-PAK4
70
50
40
GAPDH
35

## Slide 10
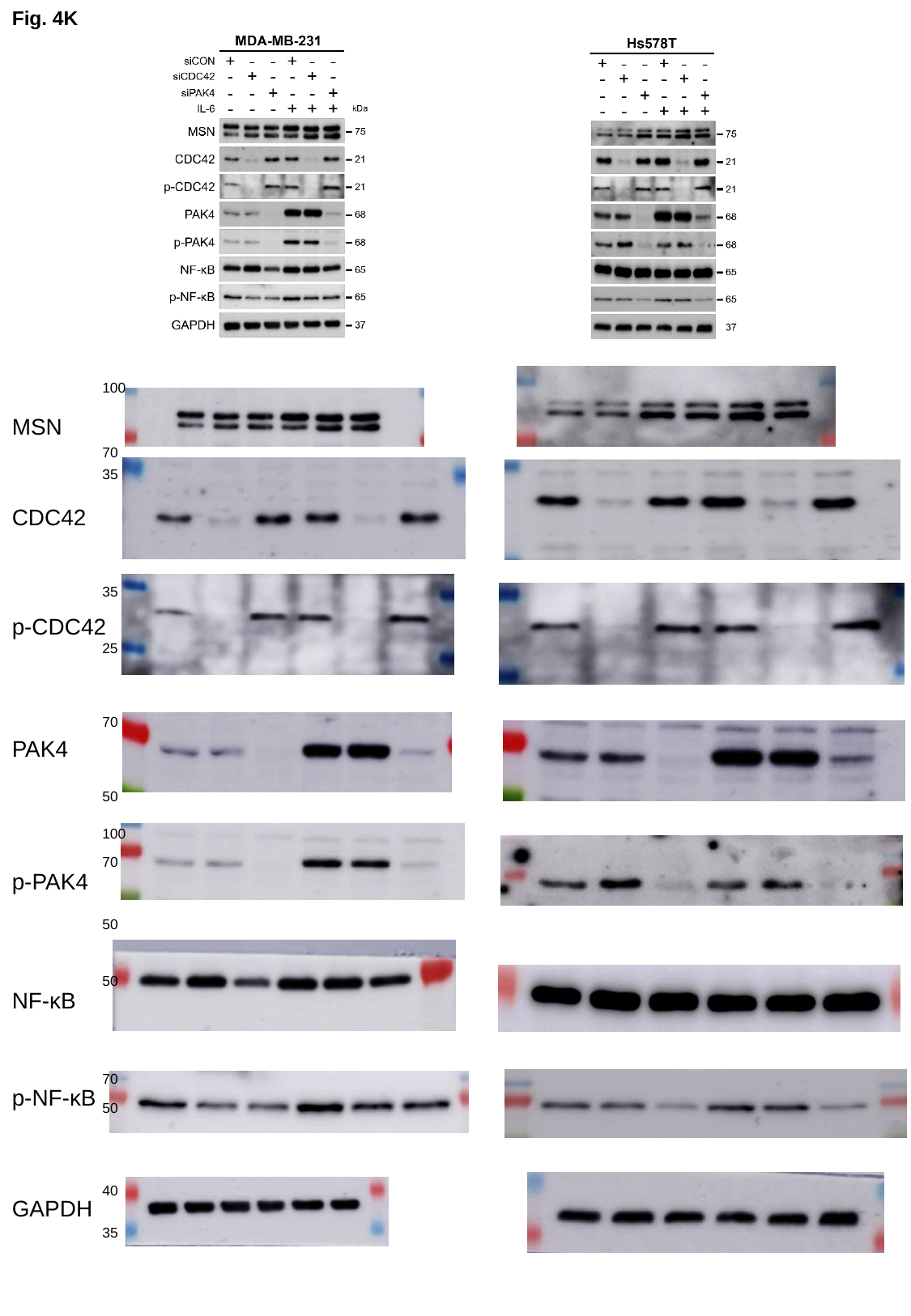

Fig. 4K
100
MSN
70
35
CDC42
35
p-CDC42
25
70
PAK4
50
100
70
p-PAK4
50
50
NF-κB
70
p-NF-κB
50
40
GAPDH
35

## Slide 11
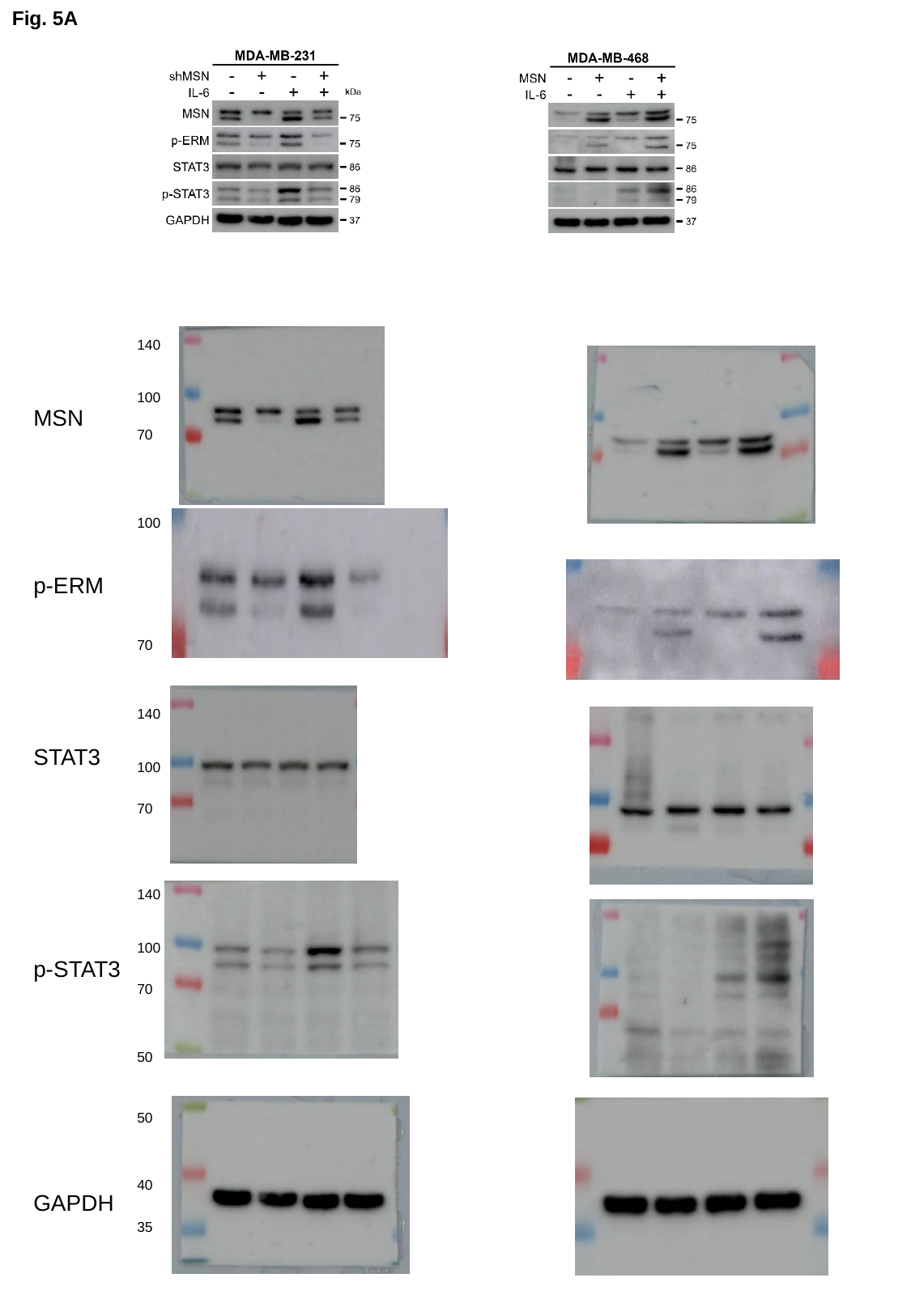

Fig. 5A
140
100
MSN
70
100
p-ERM
70
140
STAT3
100
70
140
100
p-STAT3
70
50
50
40
GAPDH
35

## Slide 12
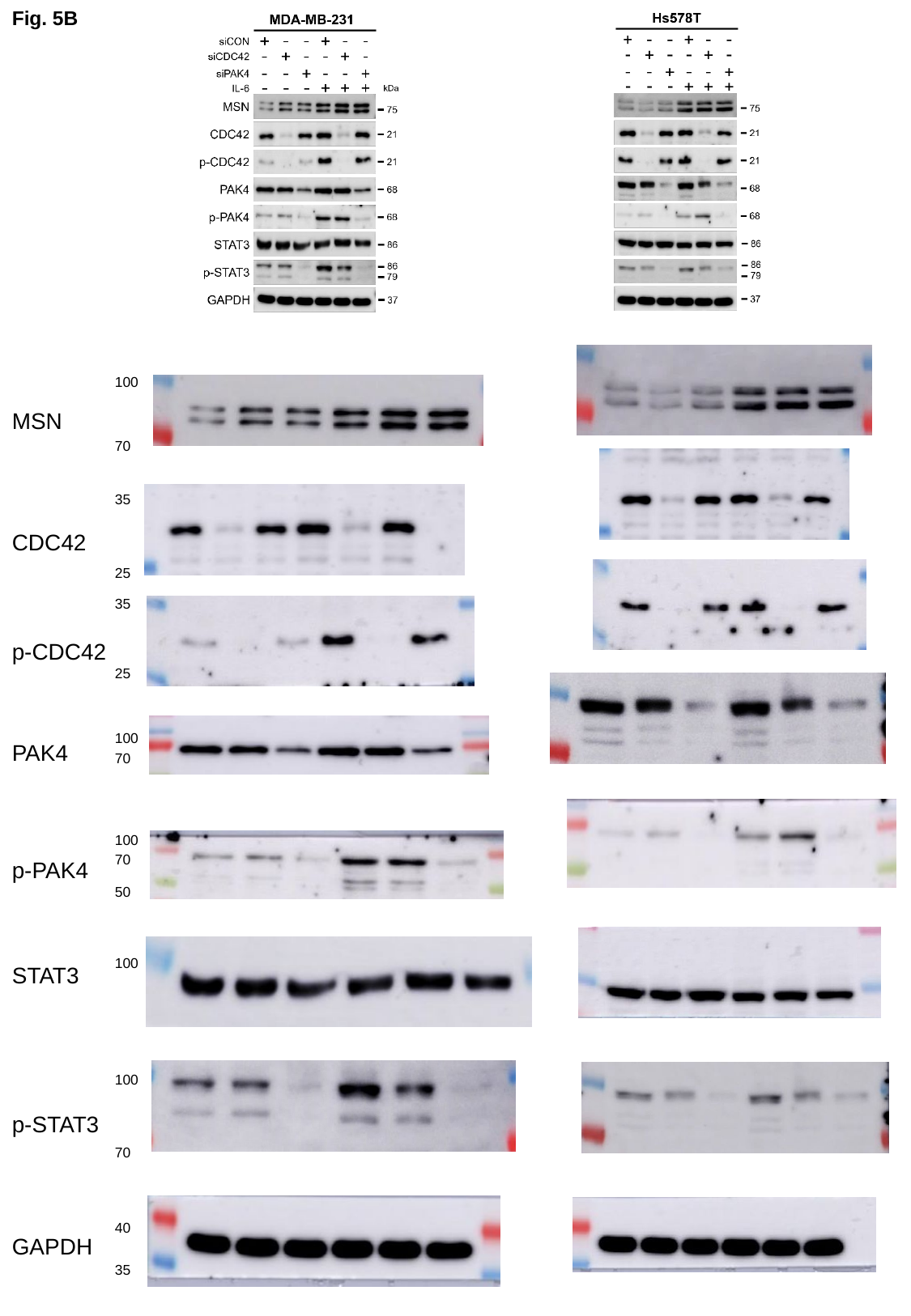

Fig. 5B
100
MSN
70
35
CDC42
25
35
p-CDC42
25
100
PAK4
70
100
70
p-PAK4
50
100
STAT3
100
p-STAT3
70
40
GAPDH
35

## Slide 13
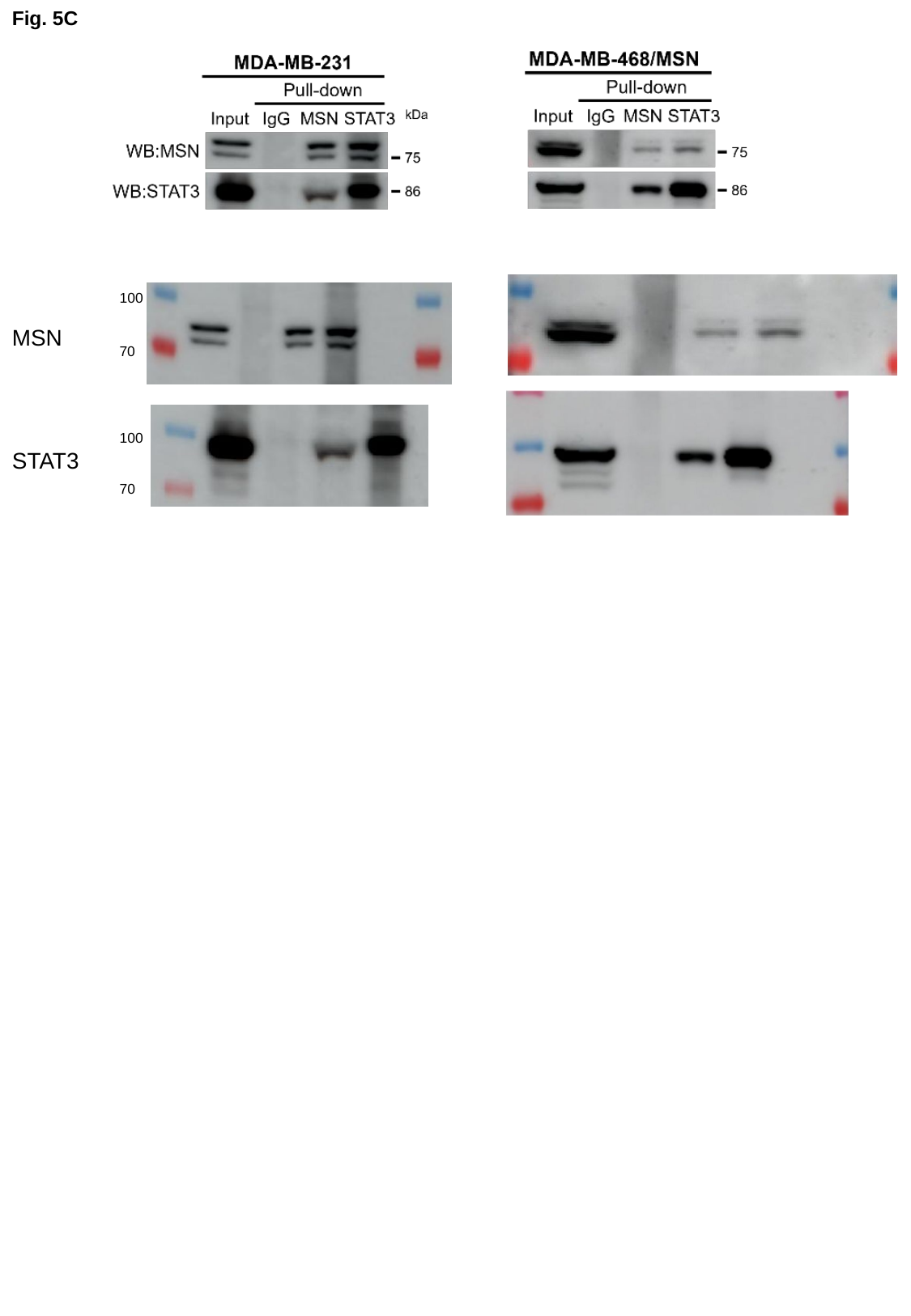

Fig. 5C
100
MSN
70
100
STAT3
70

## Slide 14
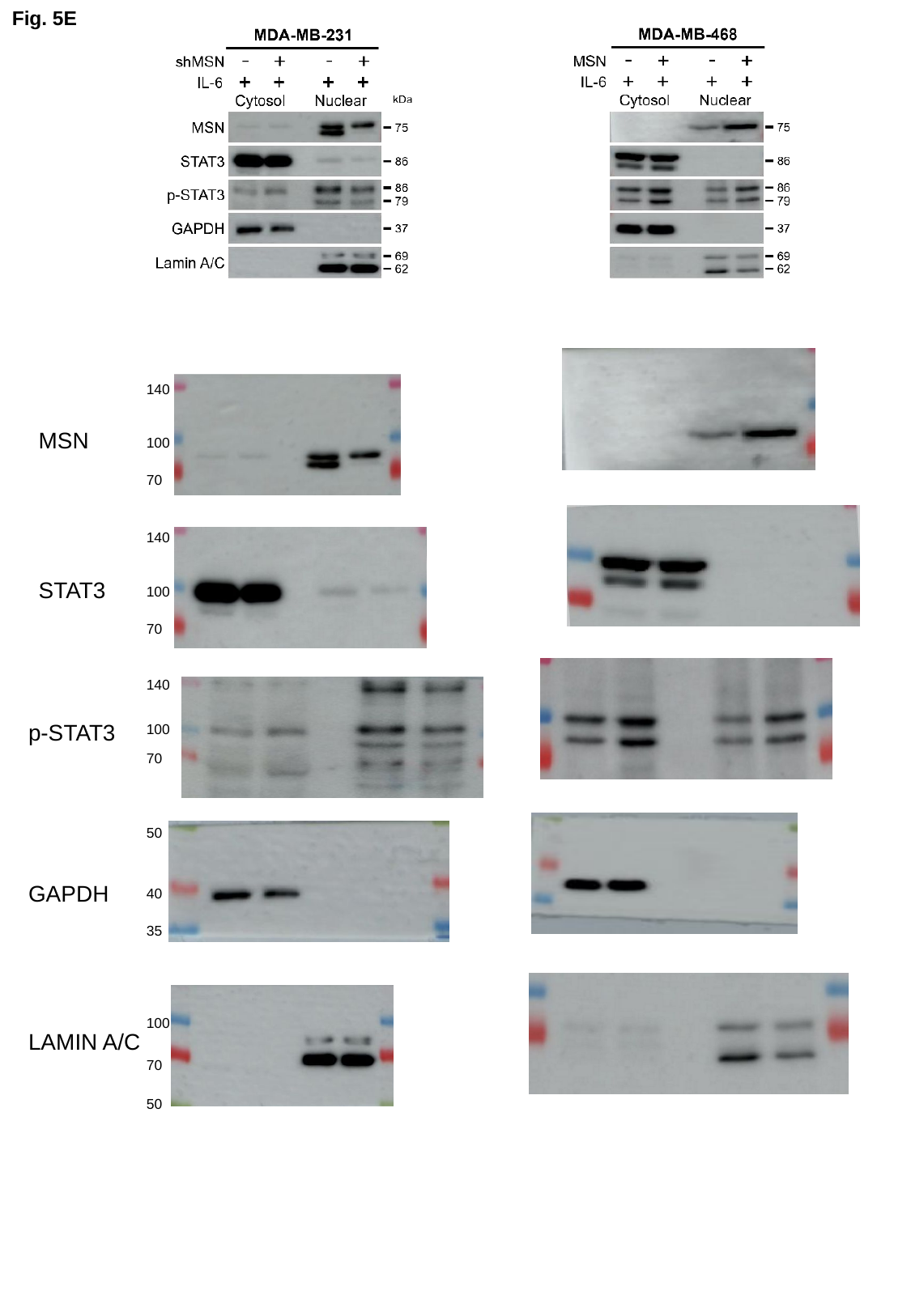

Fig. 5E
140
MSN
100
70
140
STAT3
100
70
140
p-STAT3
100
70
50
GAPDH
40
35
100
LAMIN A/C
70
50

## Slide 15
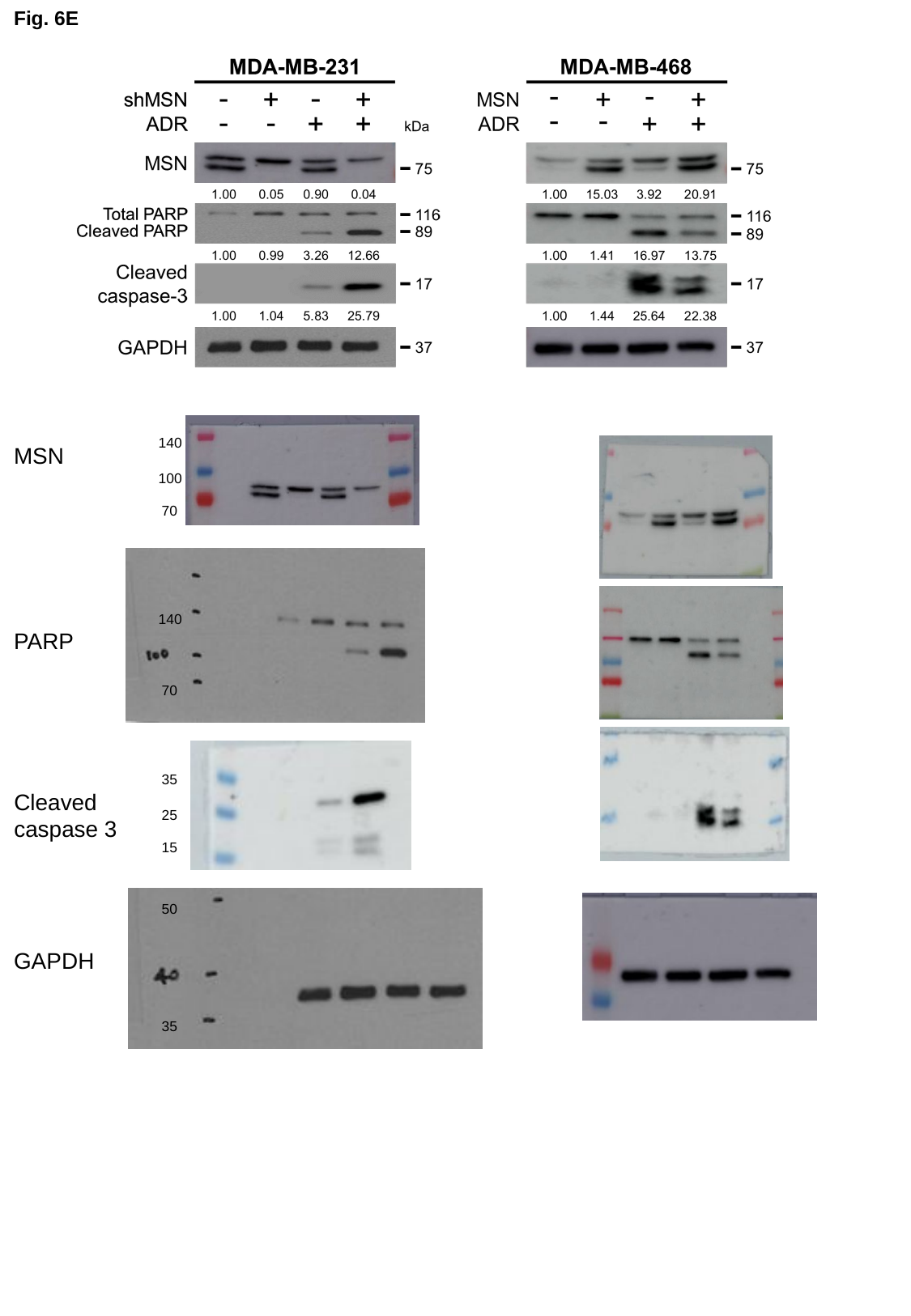

Fig. 6E
140
MSN
100
70
140
PARP
70
35
Cleaved caspase 3
25
15
50
GAPDH
35

## Slide 16
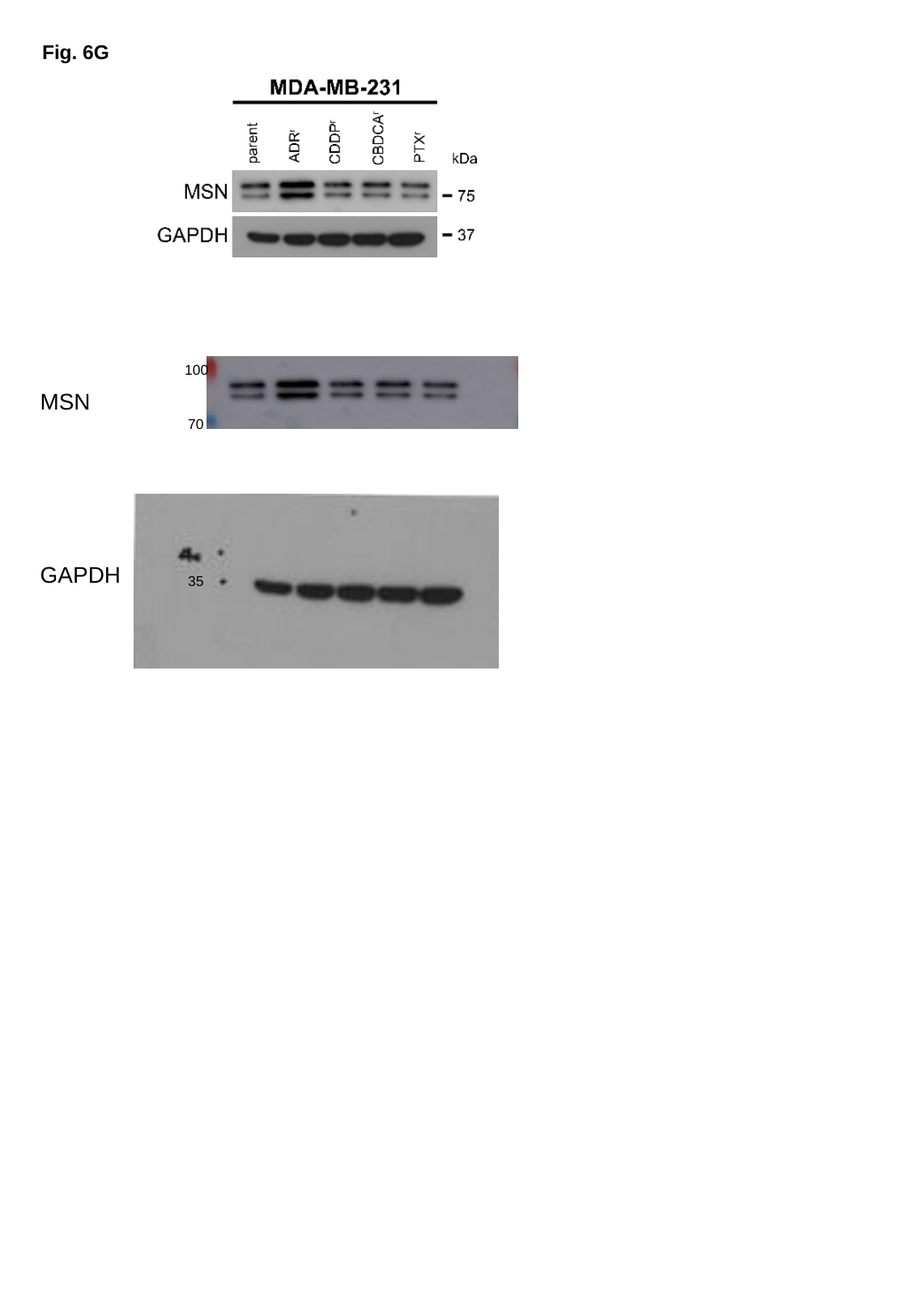

Fig. 6G
100
MSN
70
GAPDH
35

## Slide 17
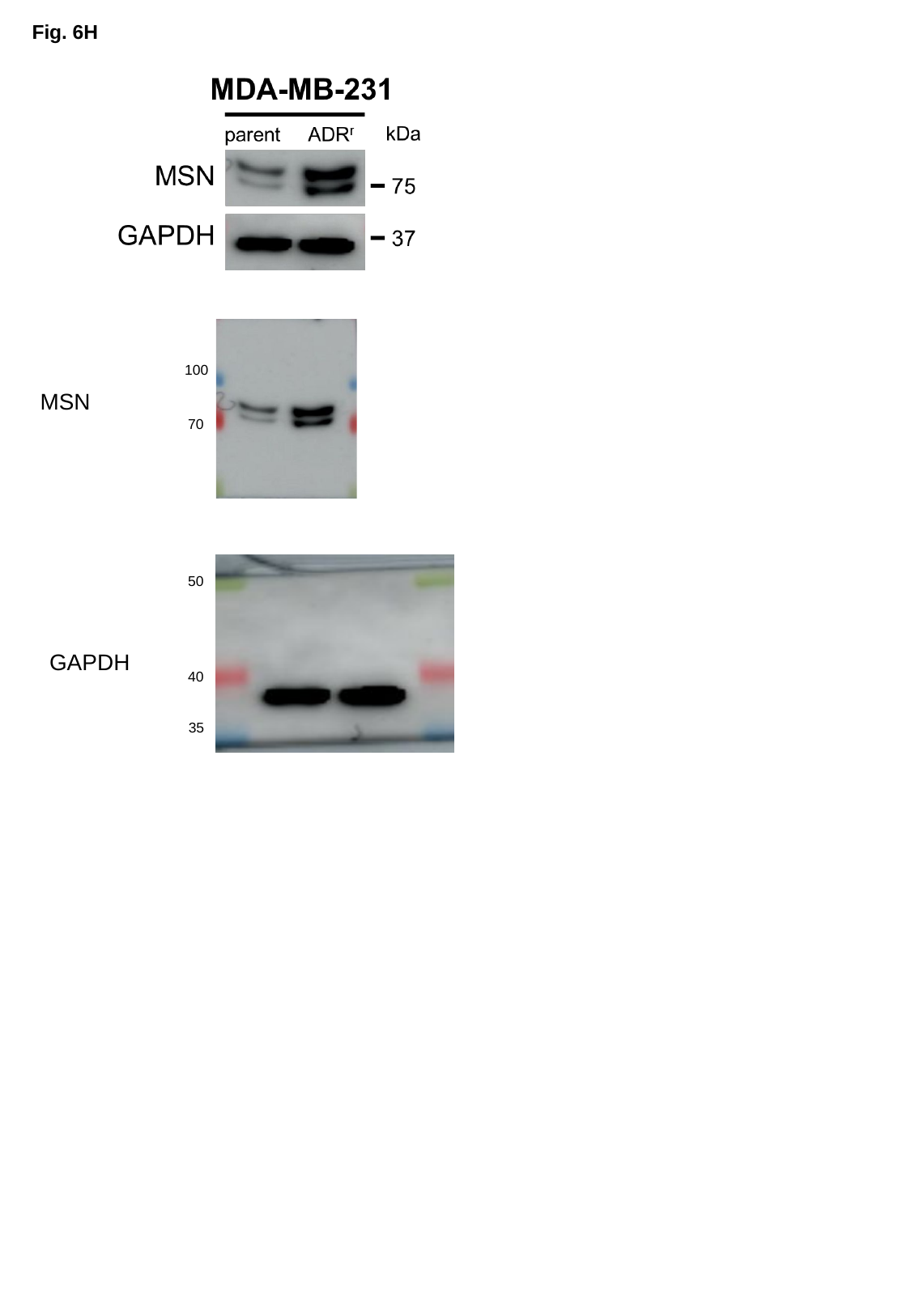

Fig. 6H
100
MSN
70
50
GAPDH
40
35

## Slide 18
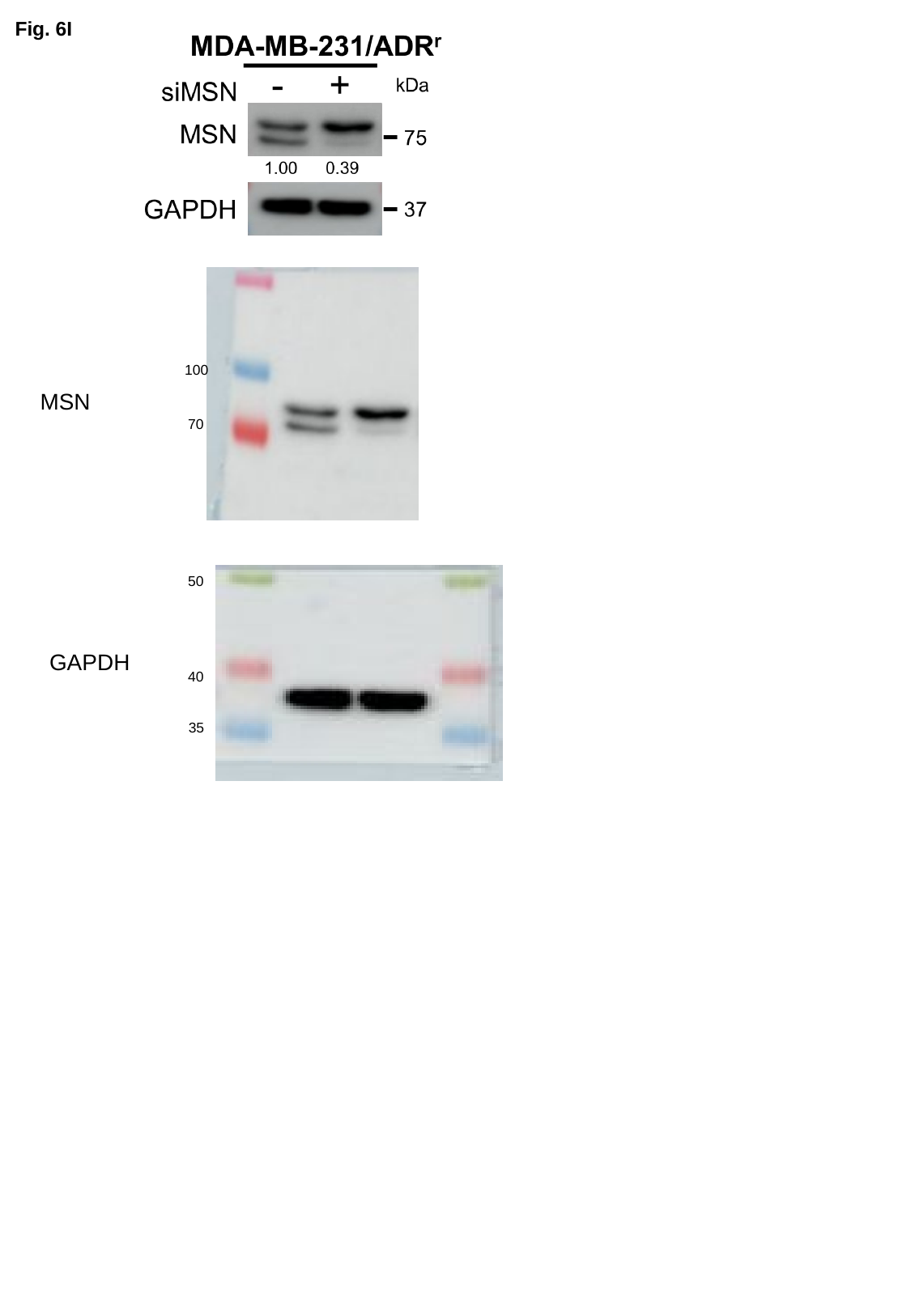

Fig. 6I
100
MSN
70
50
GAPDH
40
35

## Slide 19
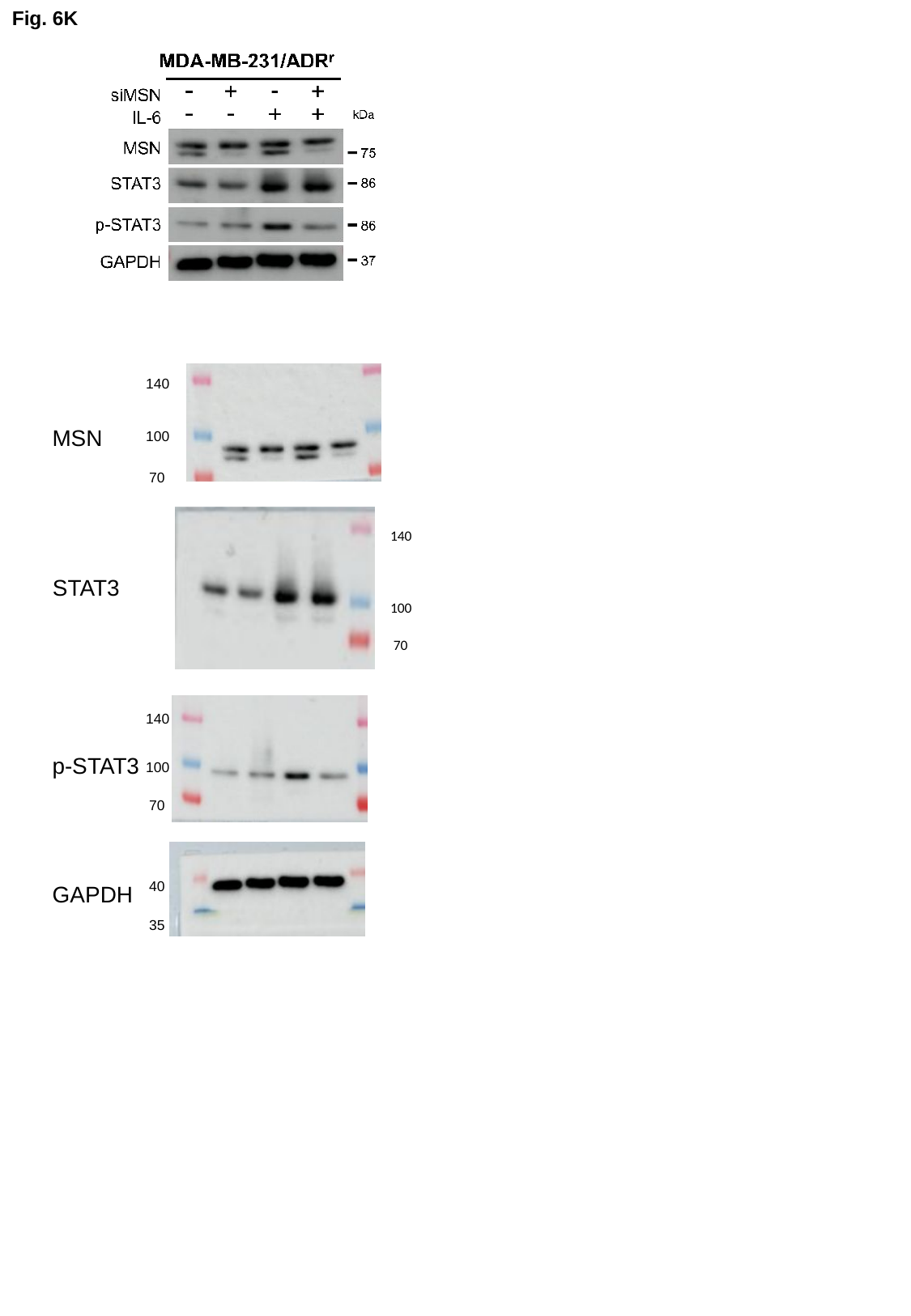

Fig. 6K
140
MSN
100
70
140
STAT3
100
70
140
p-STAT3
100
70
40
GAPDH
35

## Slide 20
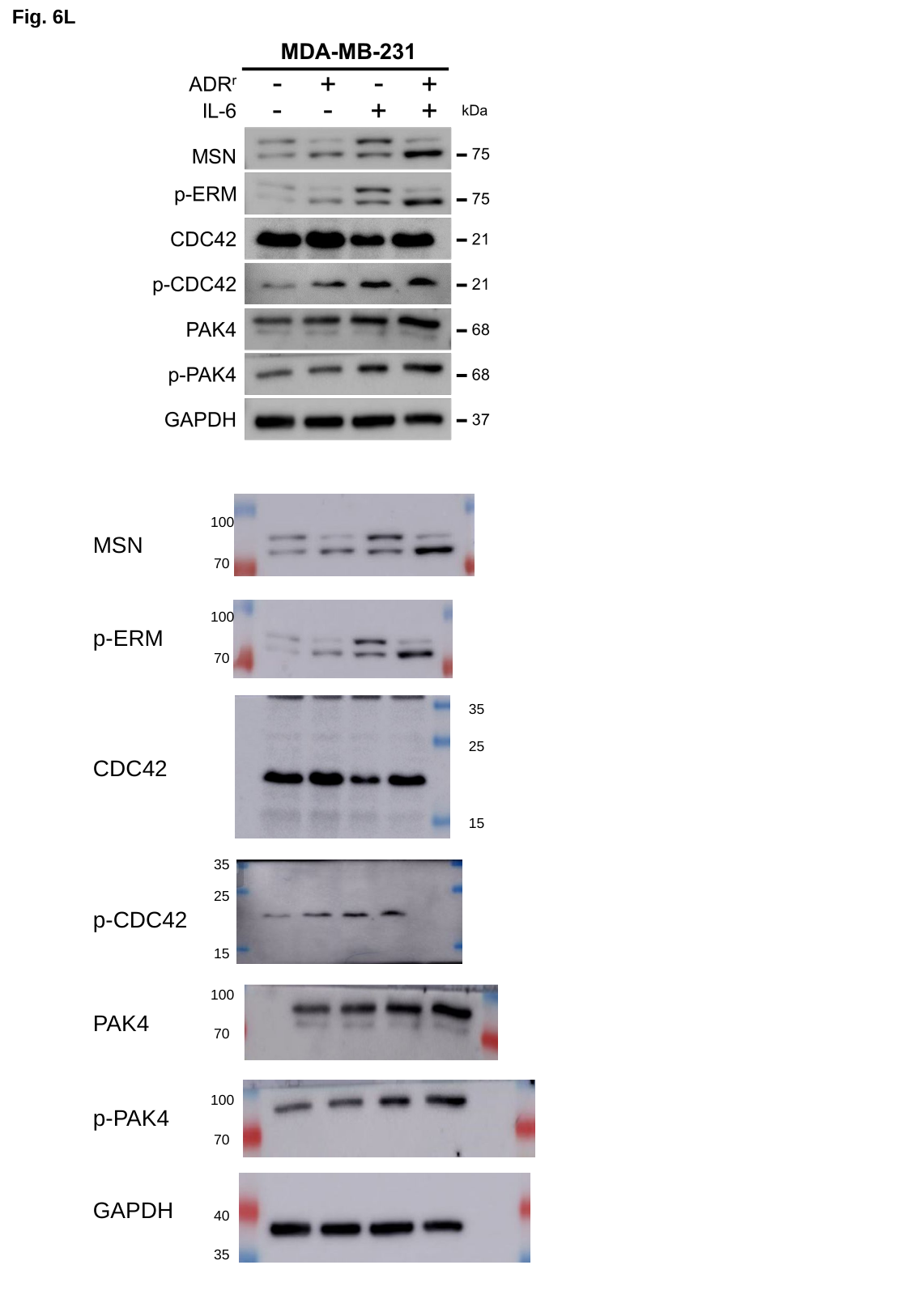

Fig. 6L
100
MSN
70
100
p-ERM
70
35
25
CDC42
15
35
25
p-CDC42
15
100
PAK4
70
100
p-PAK4
70
GAPDH
40
35

## Slide 21
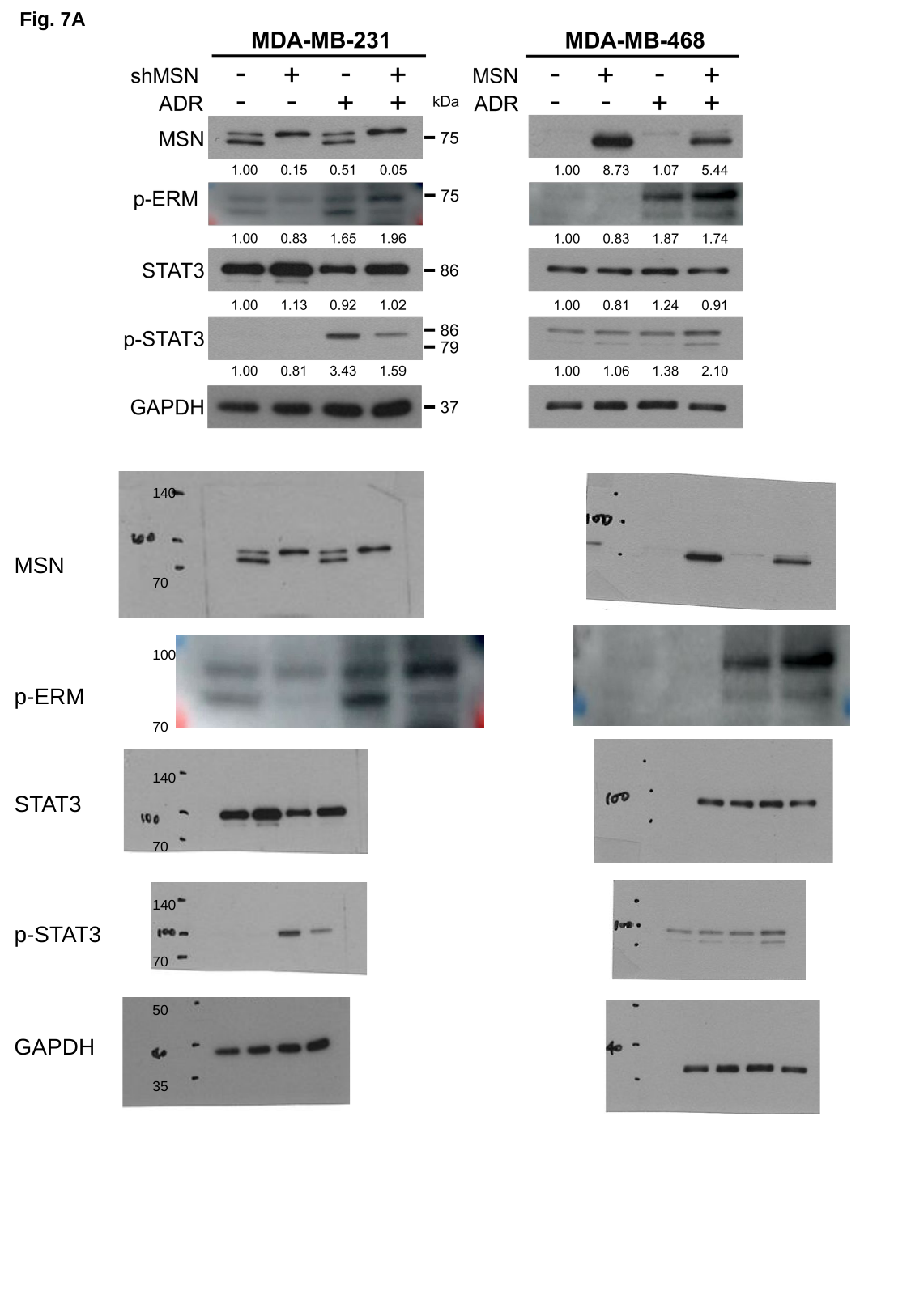

Fig. 7A
140
MSN
70
100
p-ERM
70
140
STAT3
70
140
p-STAT3
70
50
GAPDH
35

## Slide 22
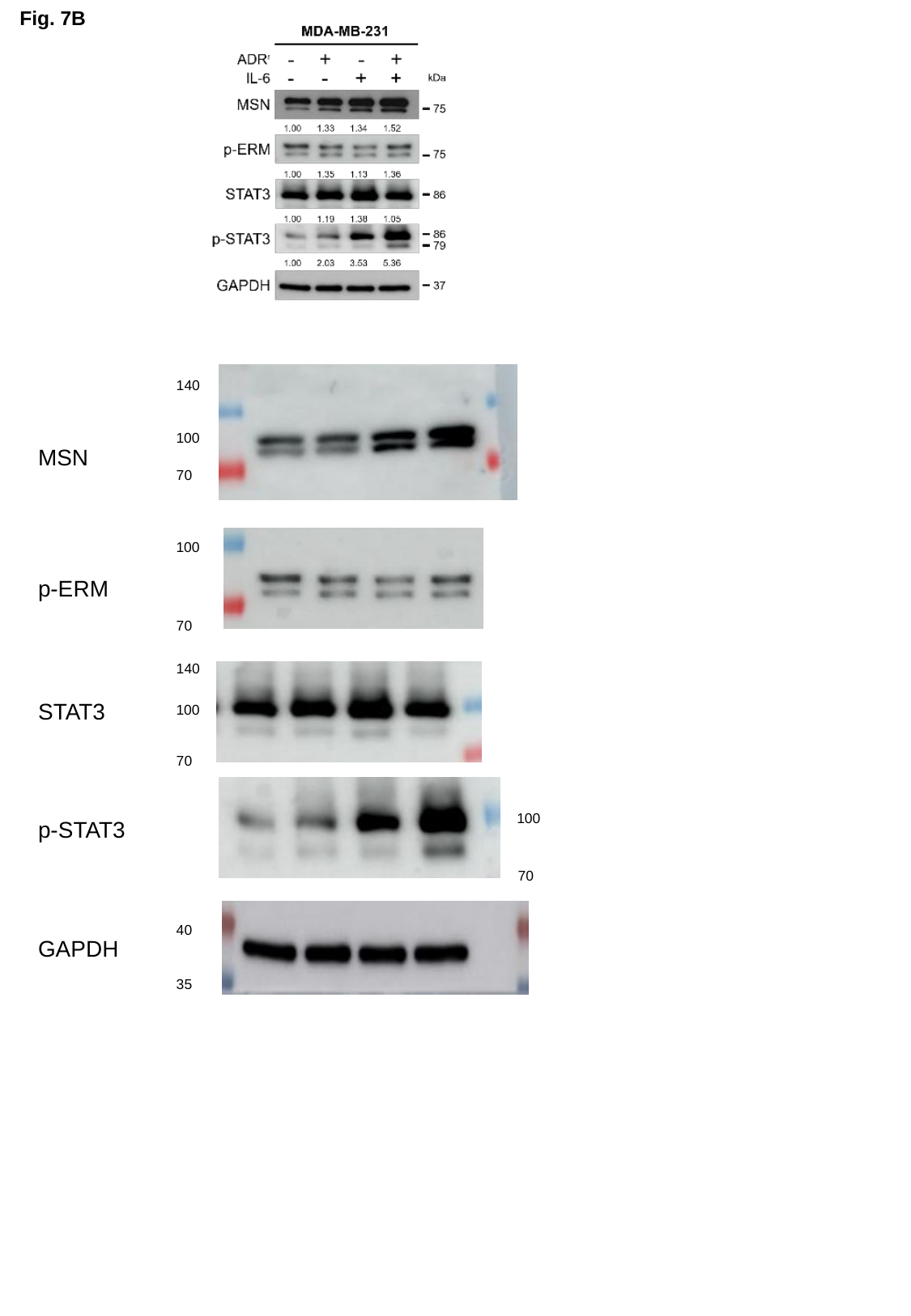

Fig. 7B
140
100
MSN
70
100
p-ERM
70
140
STAT3
100
70
100
p-STAT3
70
40
GAPDH
35

## Slide 23
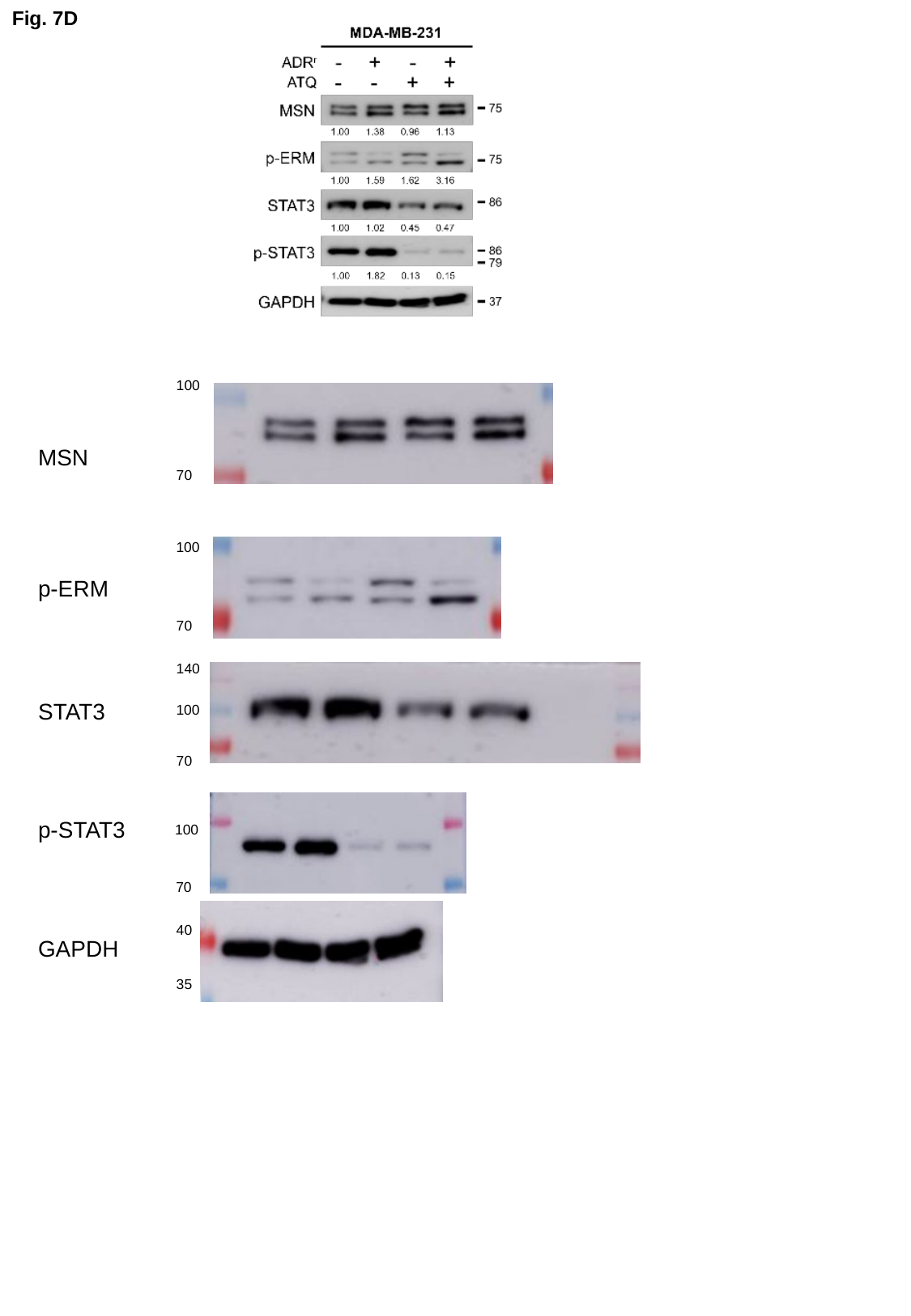

Fig. 7D
100
MSN
70
100
p-ERM
70
140
STAT3
100
70
p-STAT3
100
70
40
GAPDH
35

## Slide 24
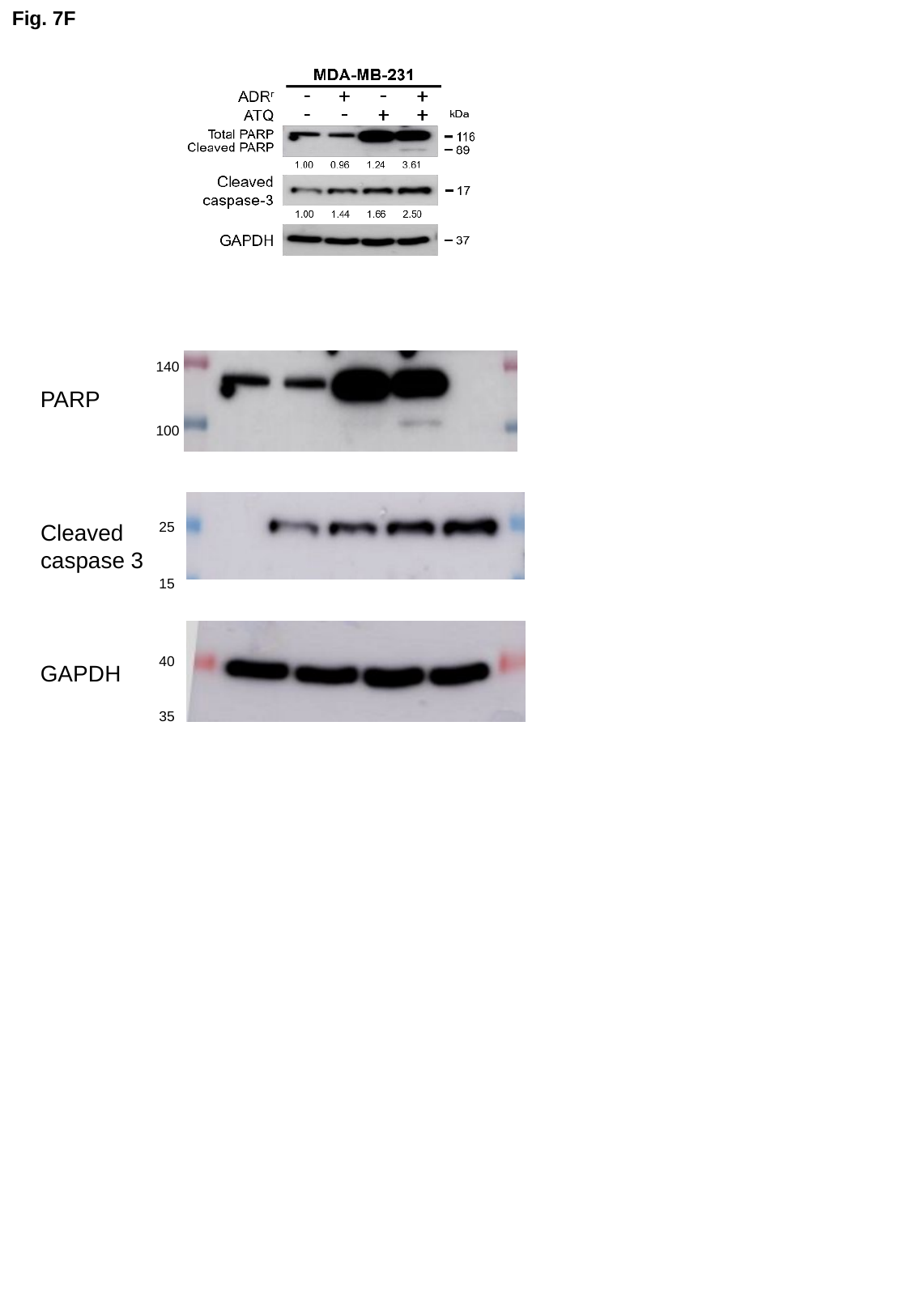

Fig. 7F
140
PARP
100
25
Cleaved caspase 3
15
40
GAPDH
35

## Slide 25
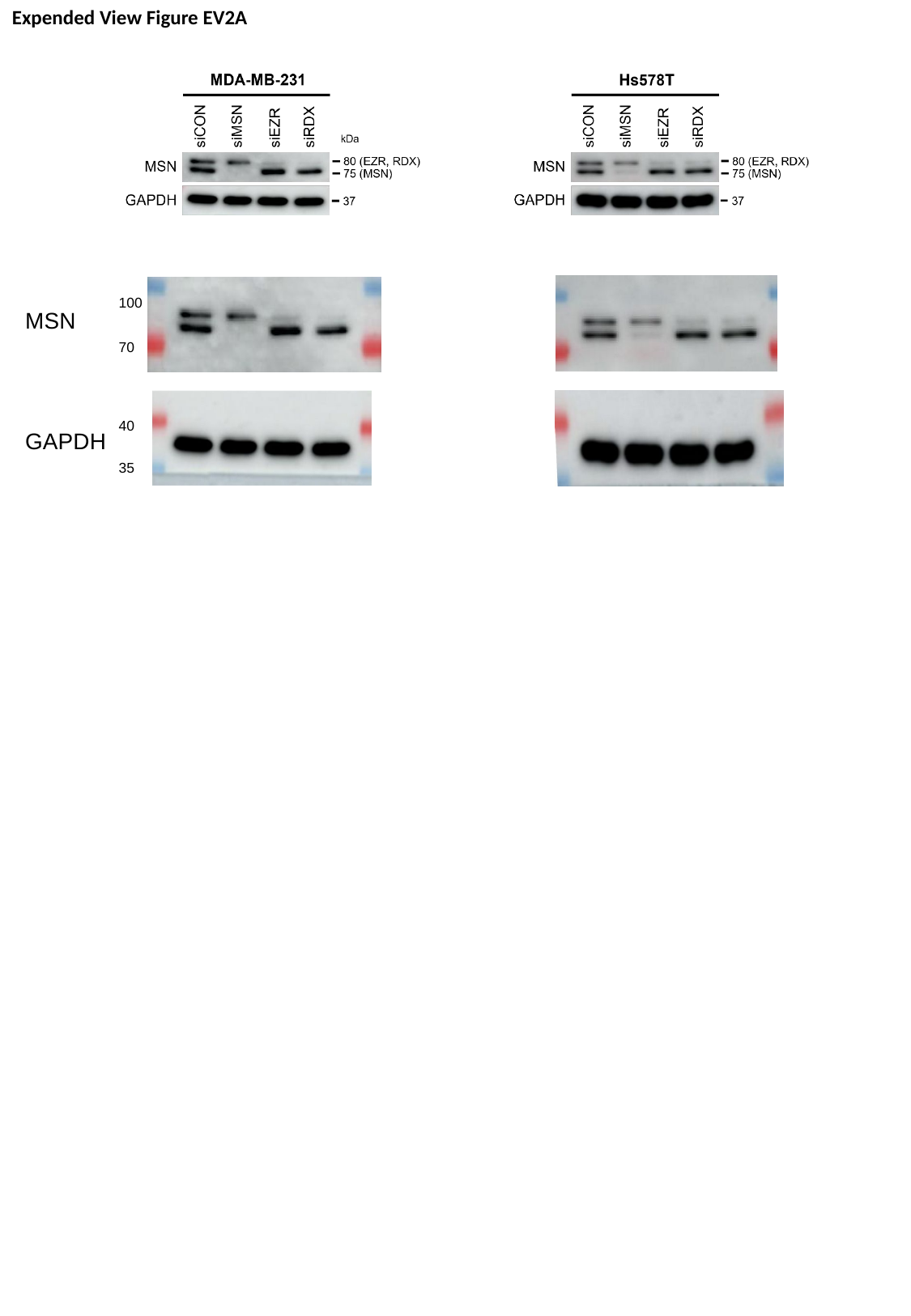

Expended View Figure EV2A
100
MSN
70
40
GAPDH
35

## Slide 26
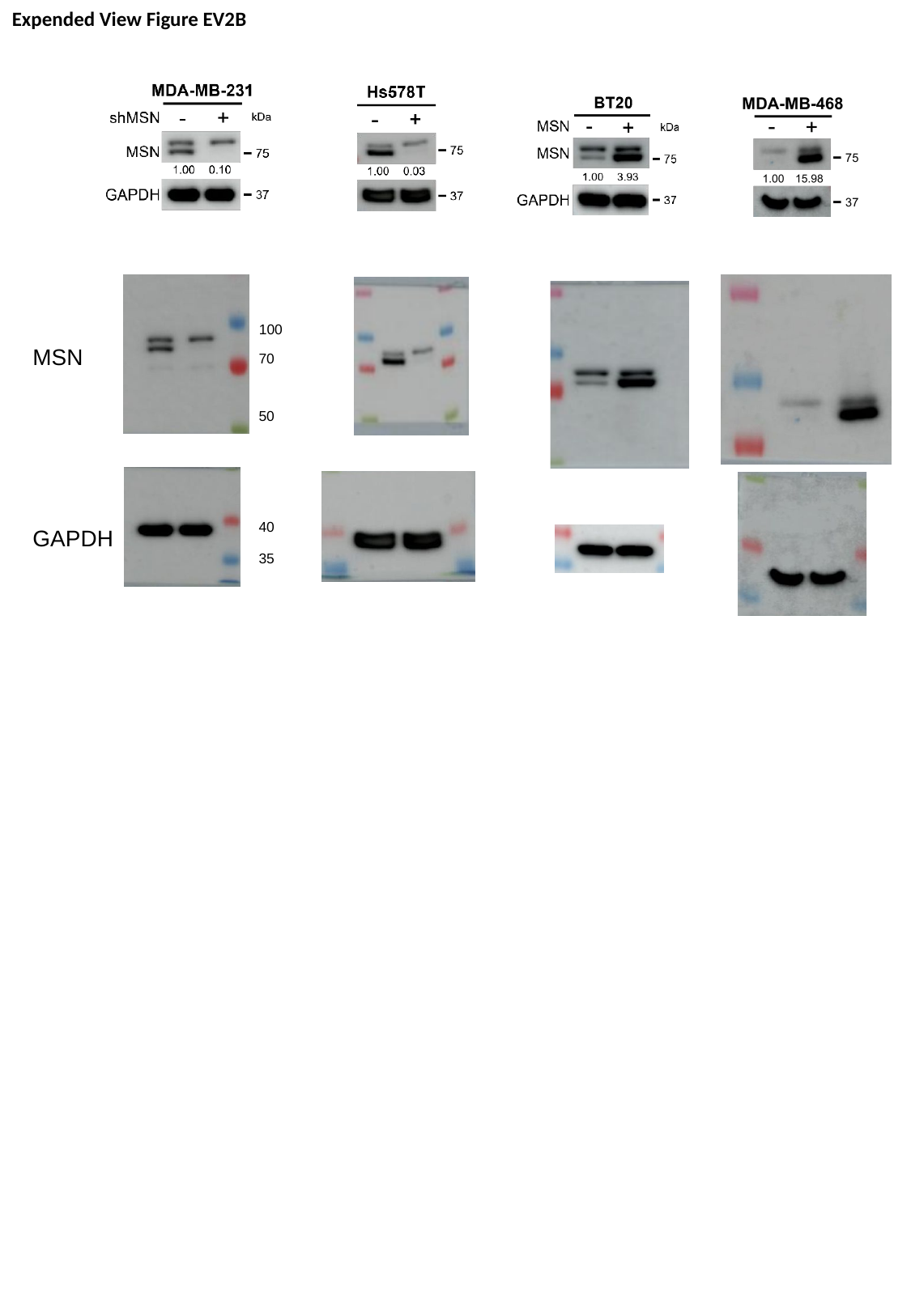

Expended View Figure EV2B
100
MSN
70
50
40
GAPDH
35

## Slide 27
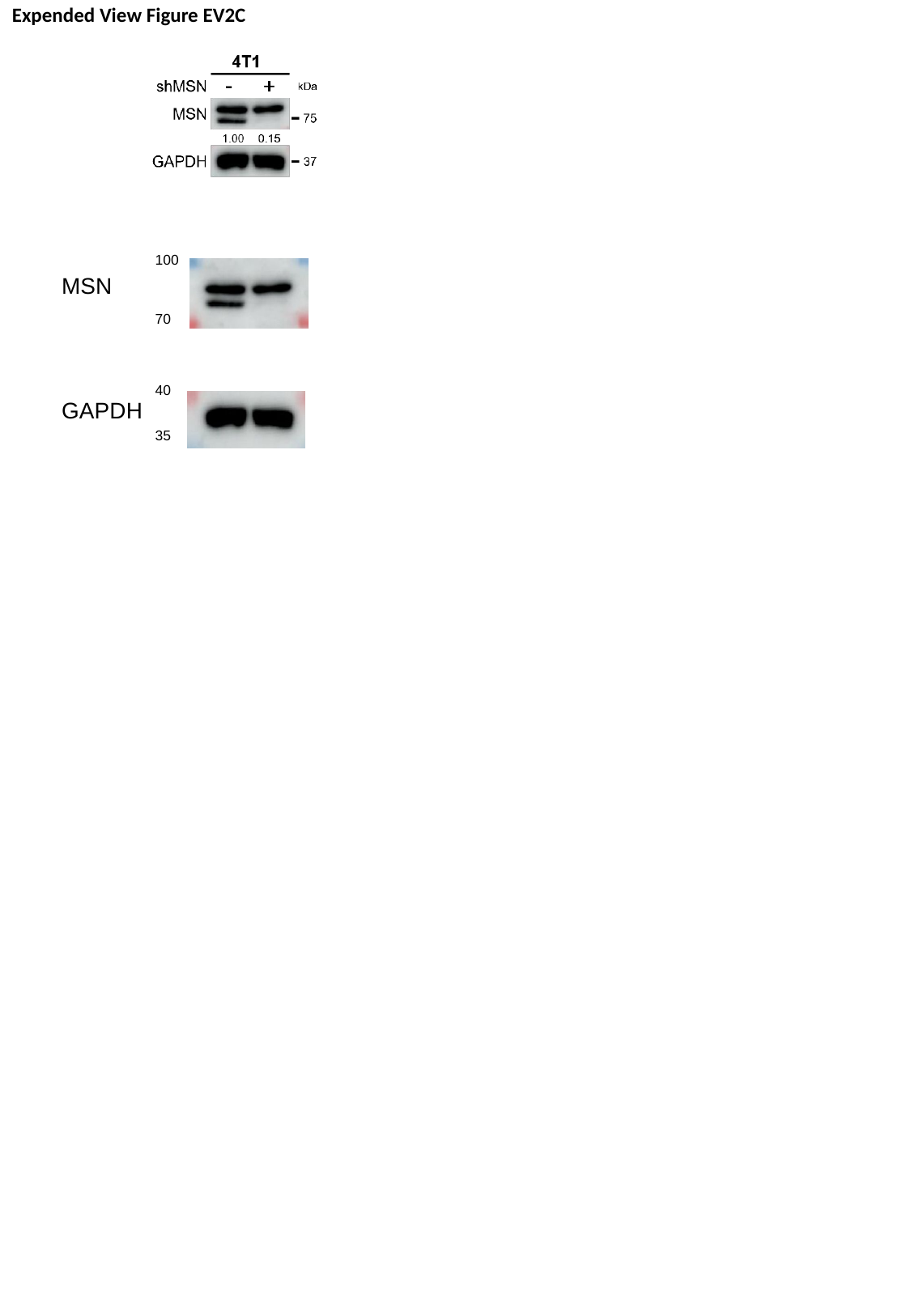

Expended View Figure EV2C
100
MSN
70
40
GAPDH
35

## Slide 28
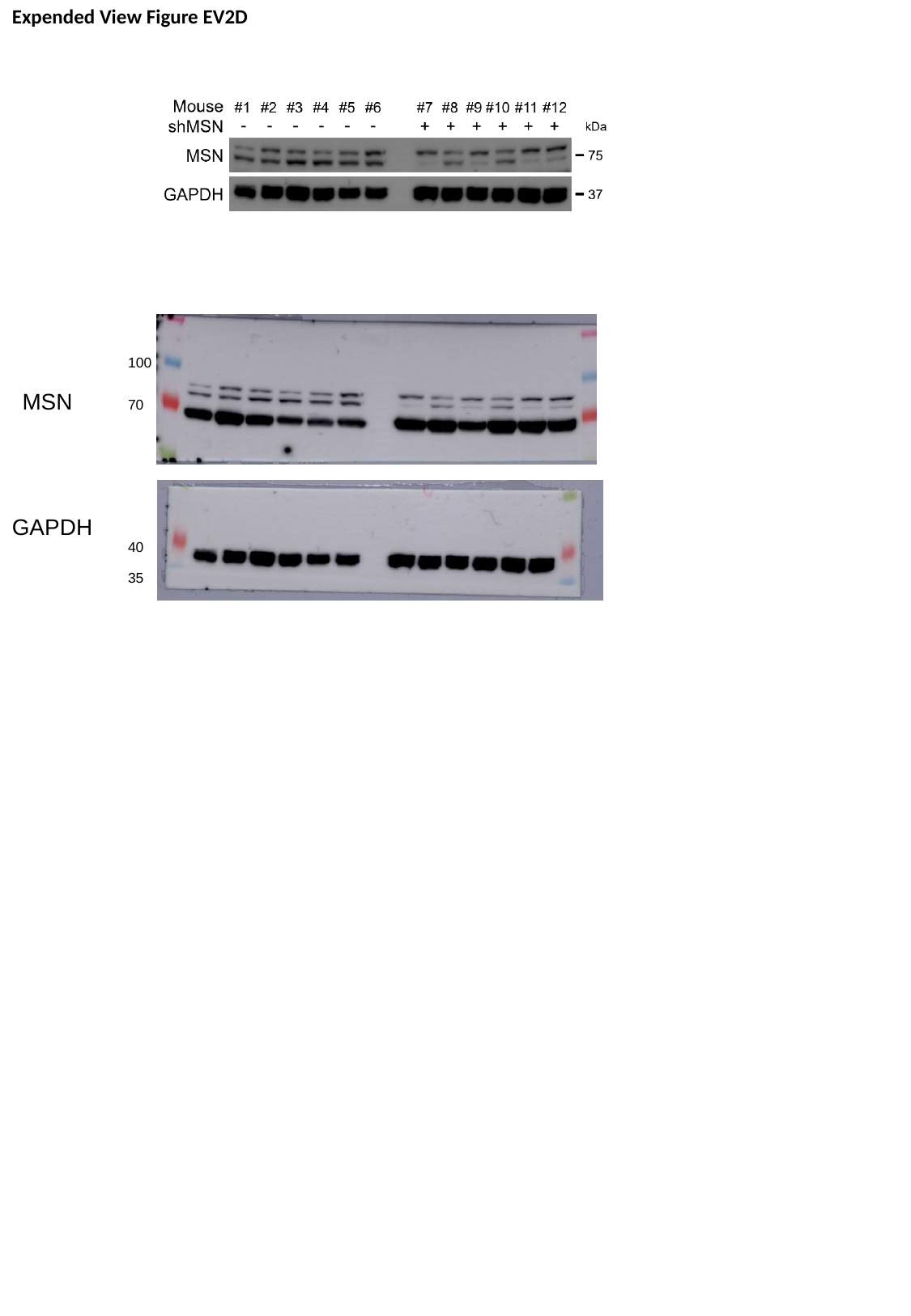

Expended View Figure EV2D
100
MSN
70
GAPDH
40
35

## Slide 29
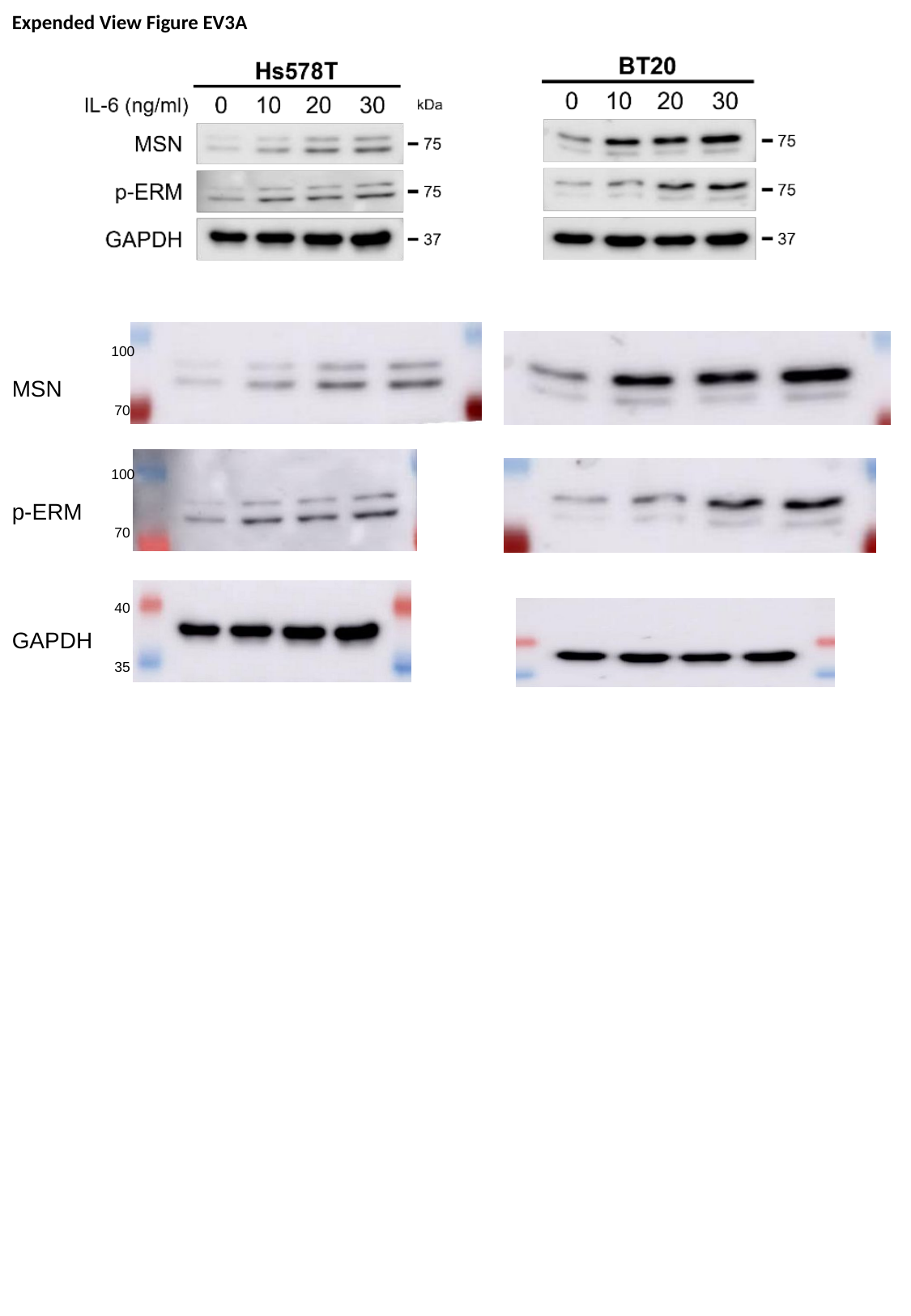

Expended View Figure EV3A
100
MSN
70
100
p-ERM
70
40
GAPDH
35

## Slide 30
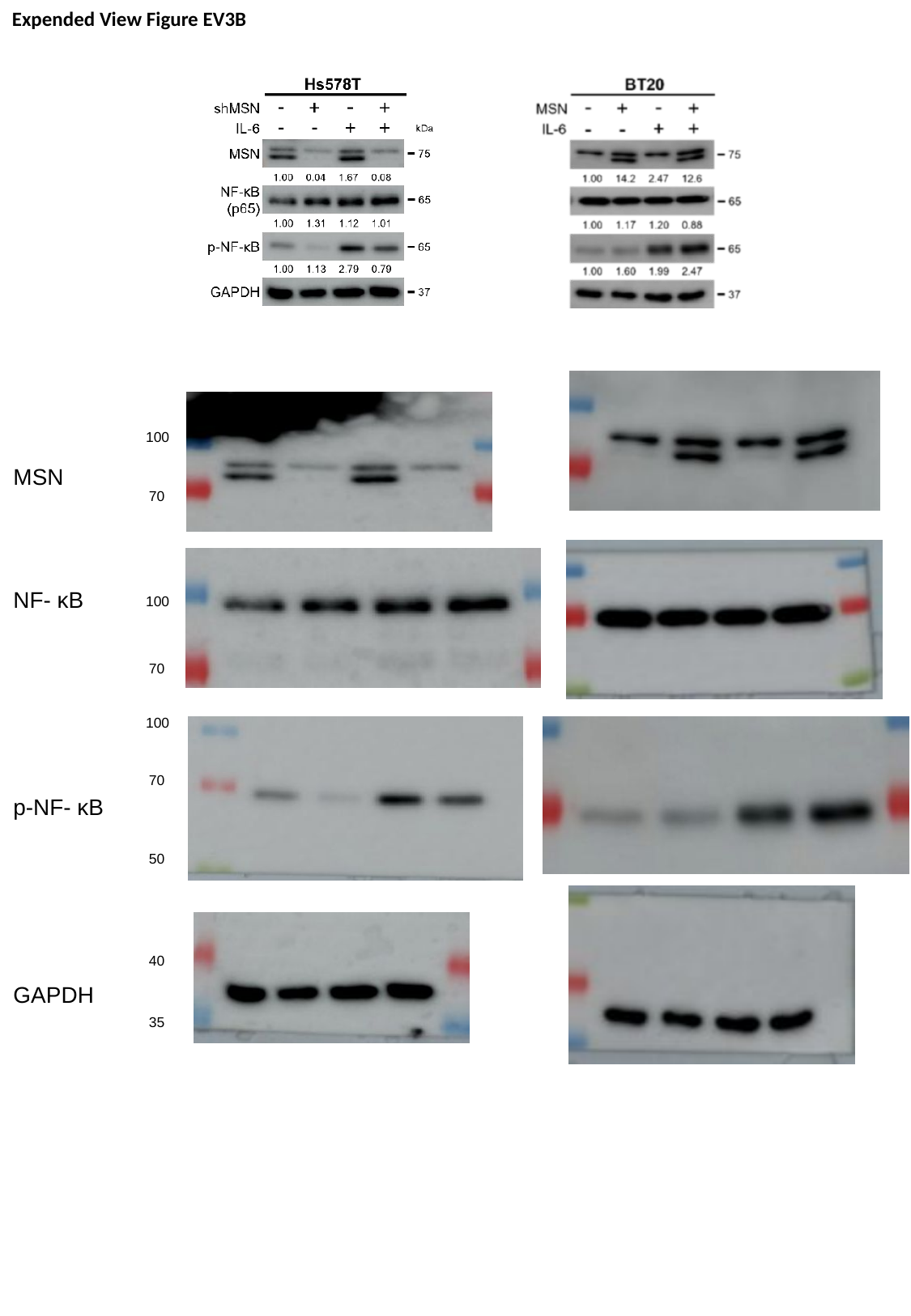

Expended View Figure EV3B
100
MSN
70
NF- κB
100
70
100
70
p-NF- κB
50
40
GAPDH
35

## Slide 31
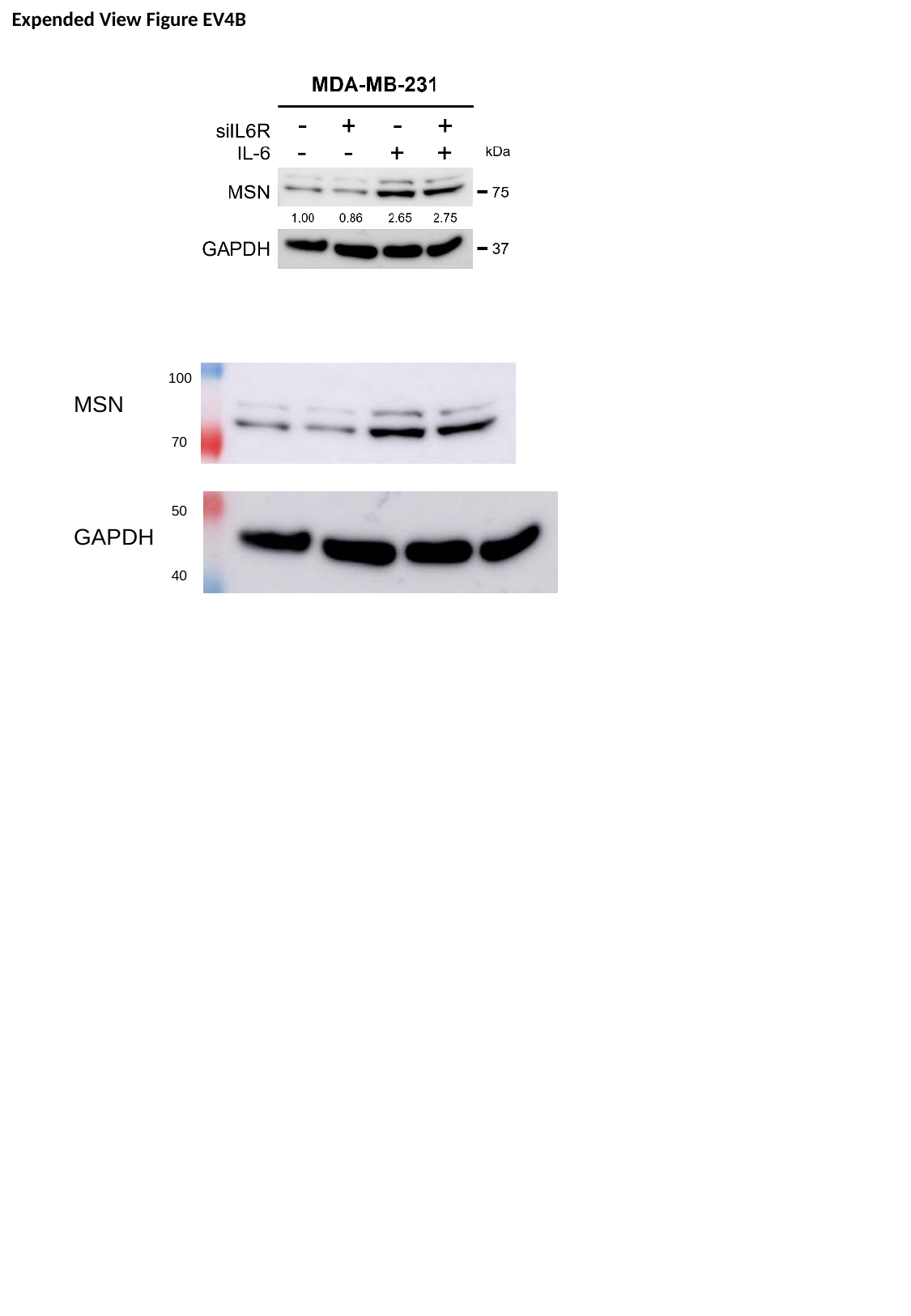

Expended View Figure EV4B
100
MSN
70
50
GAPDH
40

## Slide 32
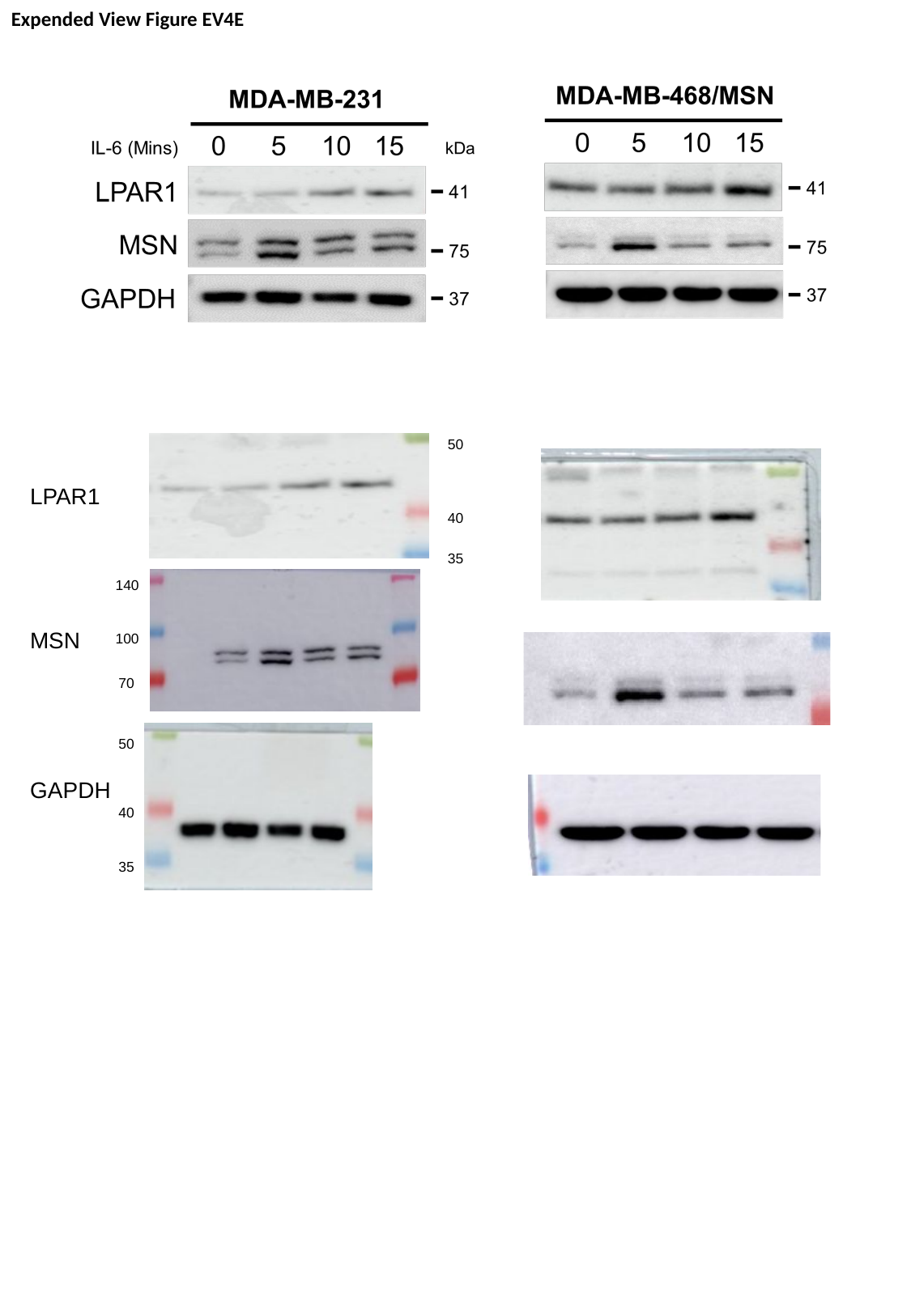

Expended View Figure EV4E
50
LPAR1
40
35
140
MSN
100
70
50
GAPDH
40
35

## Slide 33
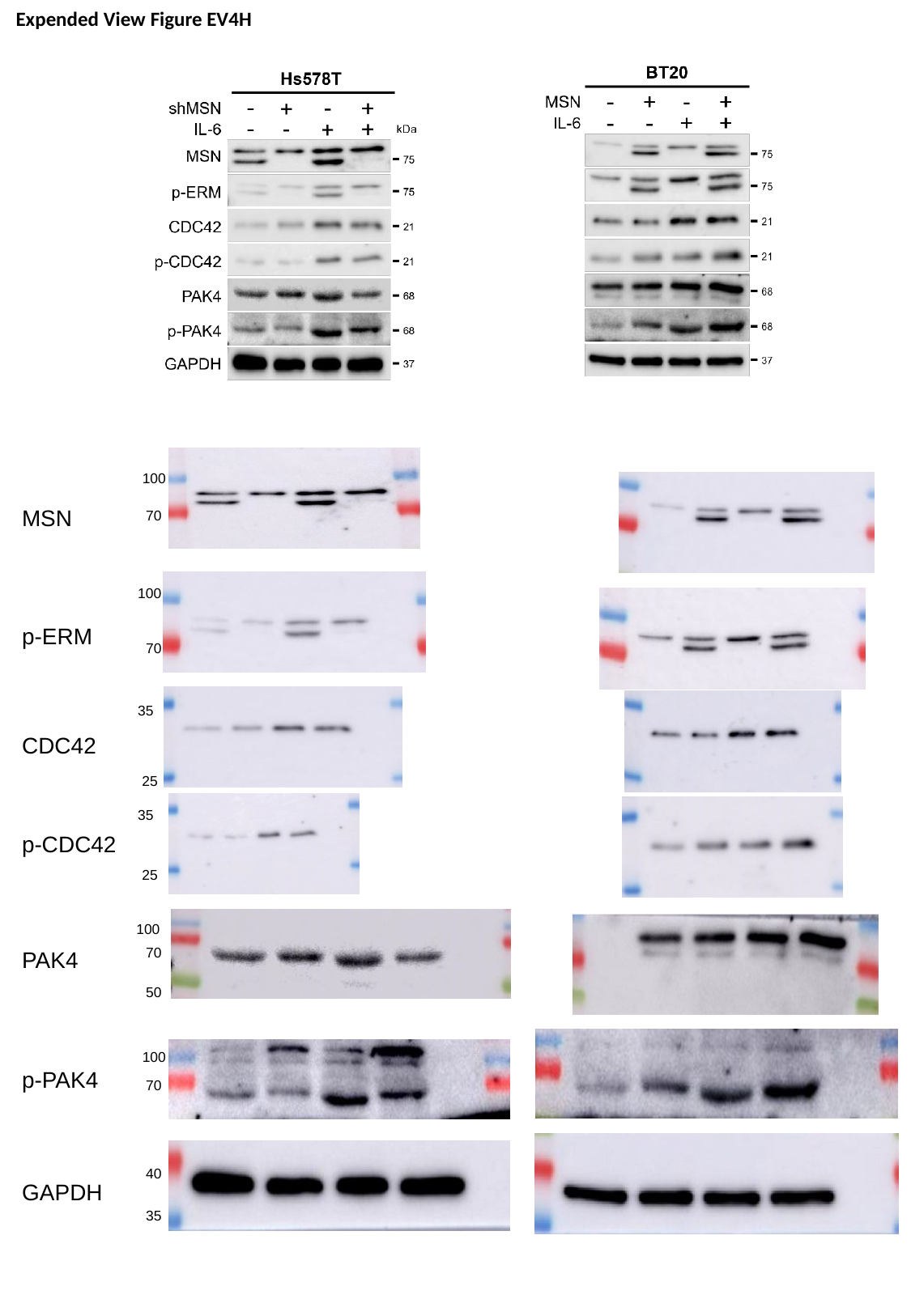

Expended View Figure EV4H
100
MSN
70
100
p-ERM
70
35
CDC42
25
35
p-CDC42
25
100
70
PAK4
50
100
p-PAK4
70
40
GAPDH
35

## Slide 34
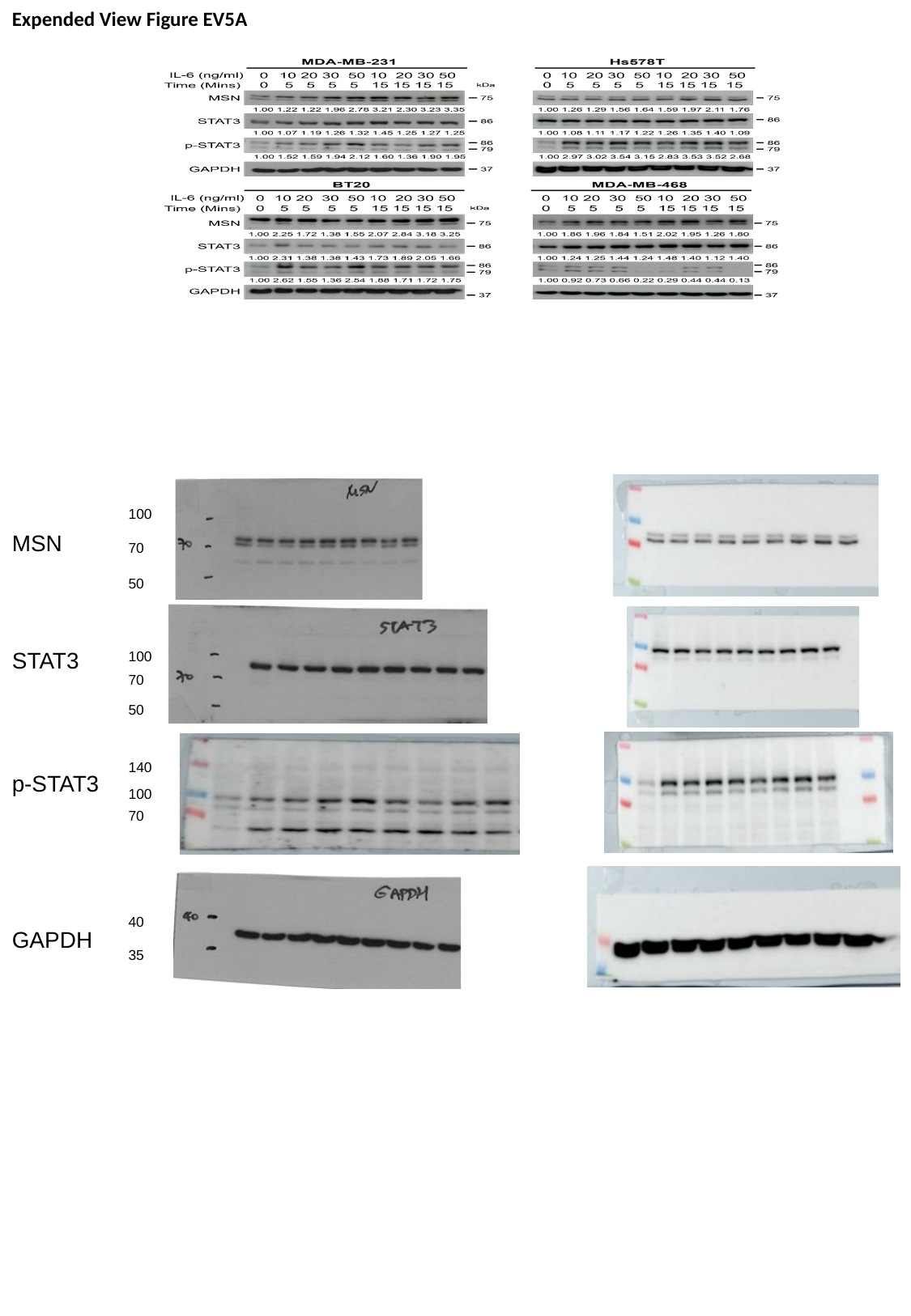

Expended View Figure EV5A
100
MSN
70
50
STAT3
100
70
50
140
p-STAT3
100
70
40
GAPDH
35

## Slide 35
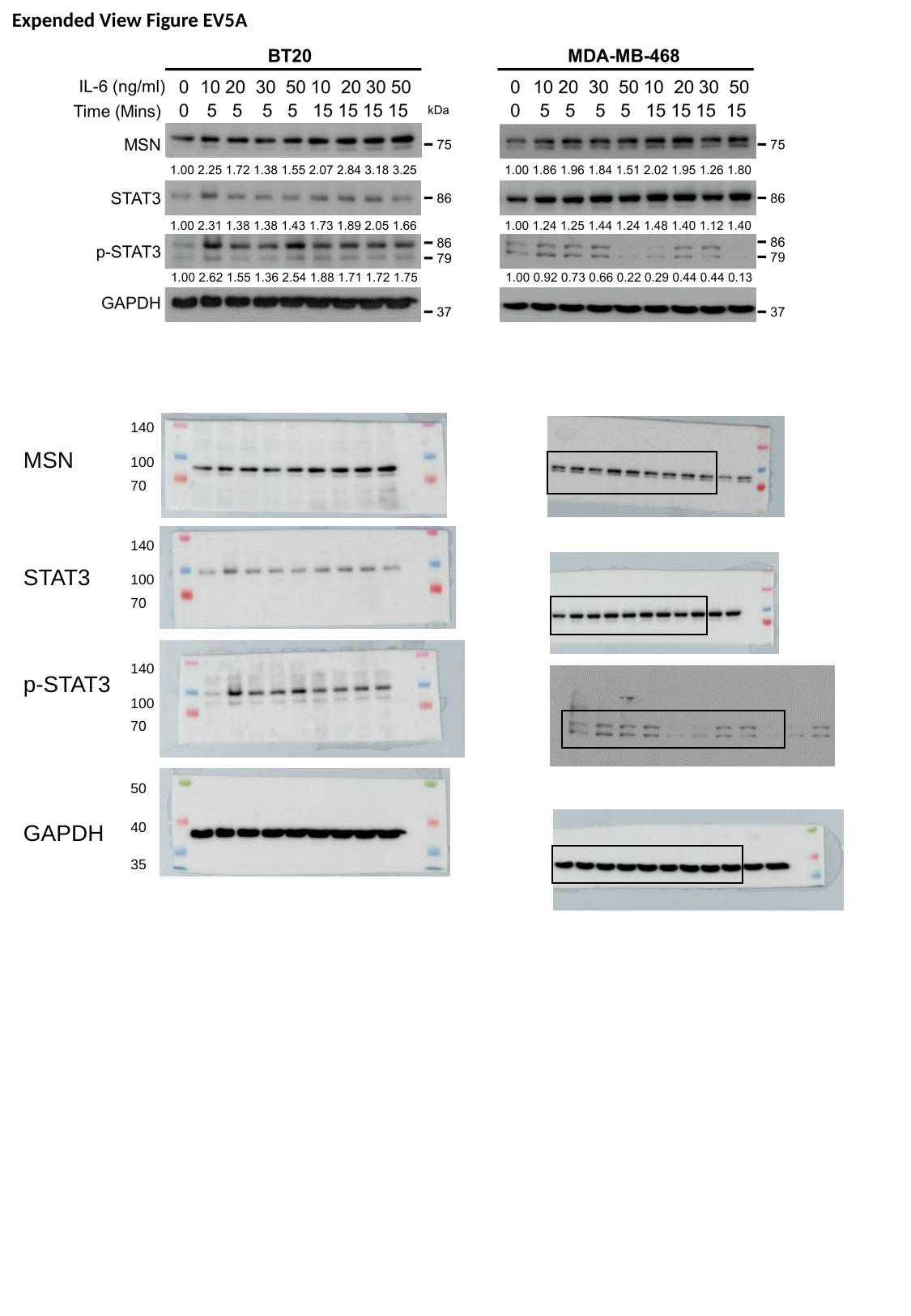

Expended View Figure EV5A
140
MSN
100
70
140
STAT3
100
70
140
p-STAT3
100
70
50
40
GAPDH
35

## Slide 36
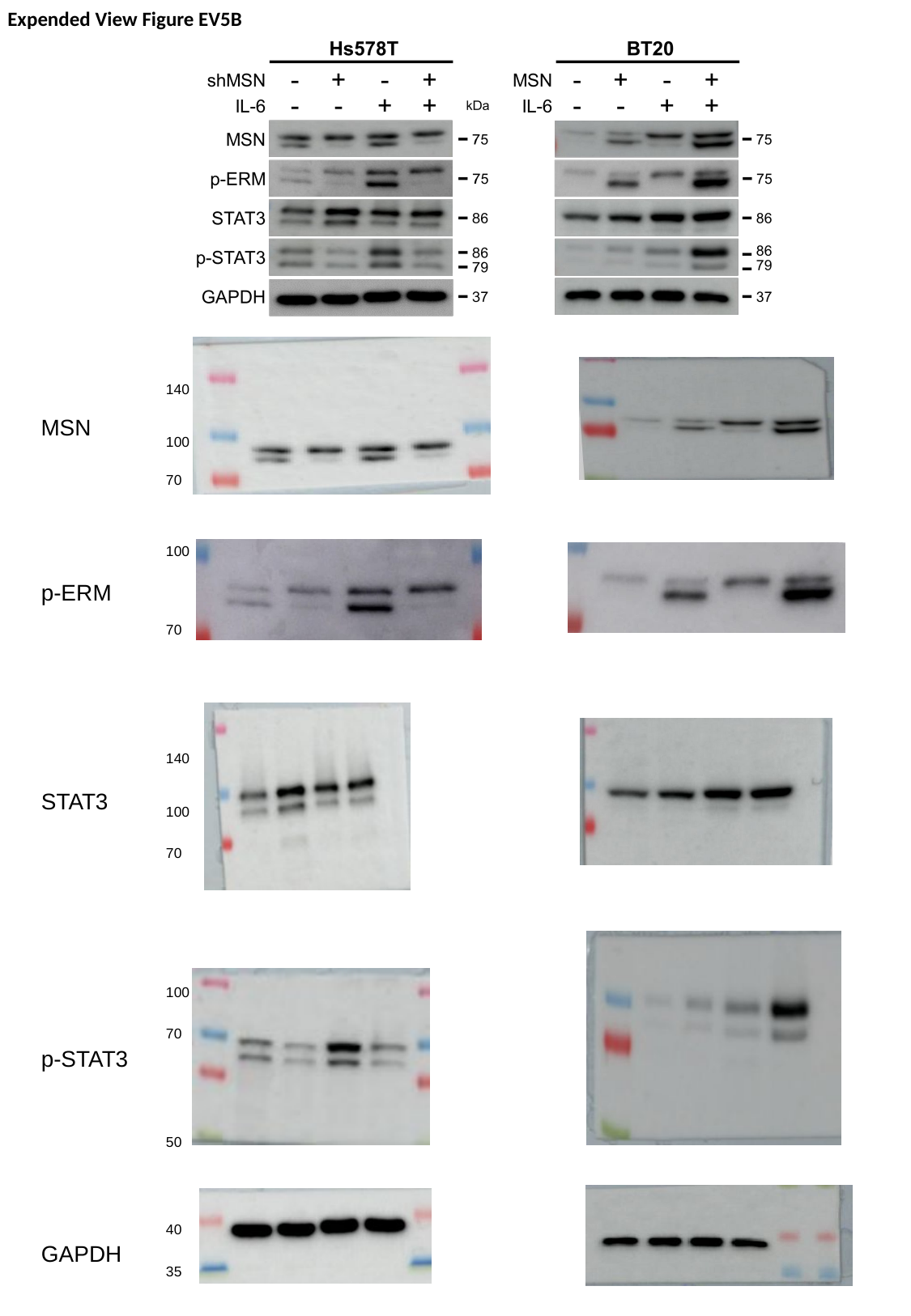

Expended View Figure EV5B
140
MSN
100
70
100
p-ERM
70
140
STAT3
100
70
100
70
p-STAT3
50
40
GAPDH
35

## Slide 37
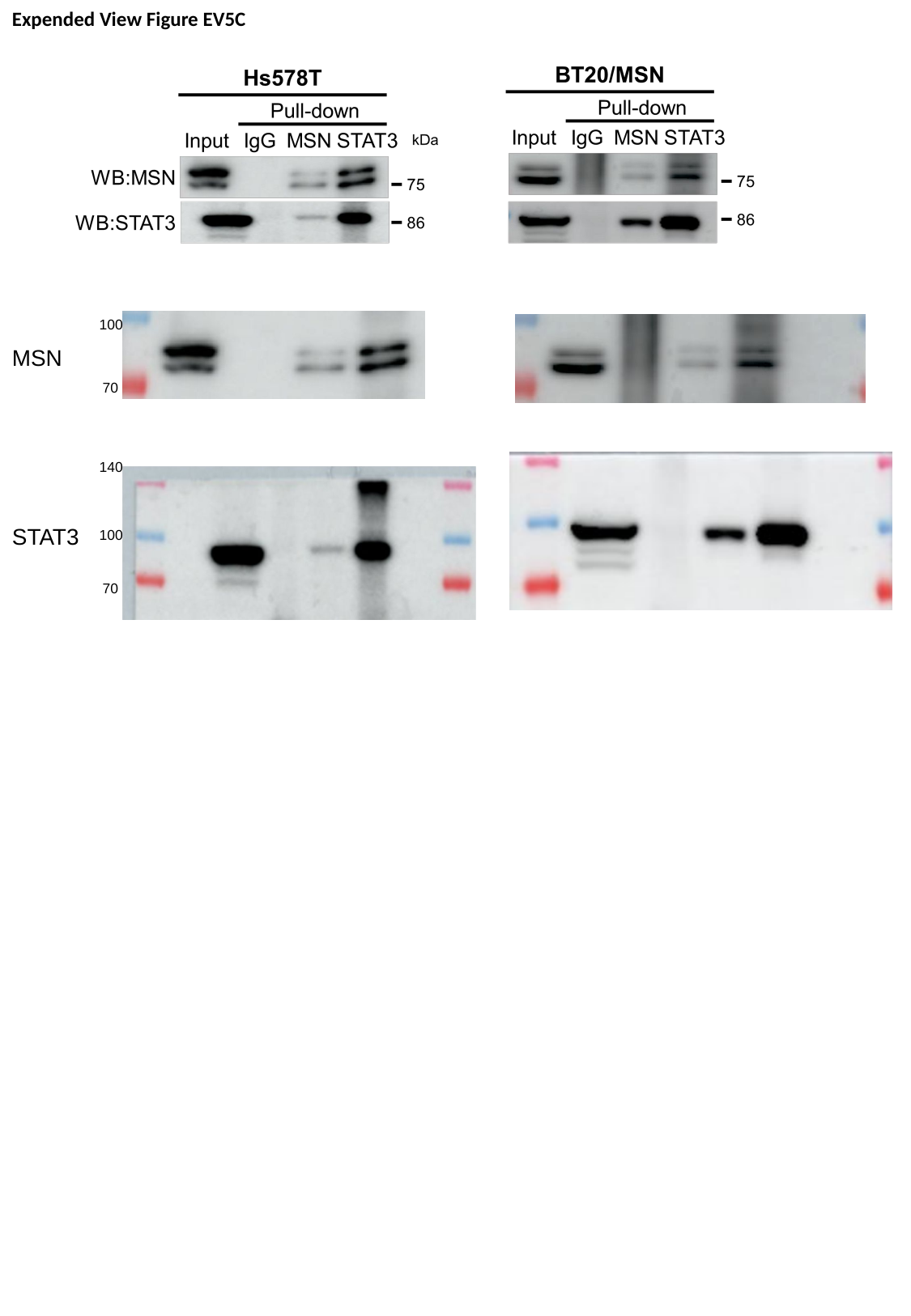

Expended View Figure EV5C
100
MSN
70
140
STAT3
100
70

## Slide 38
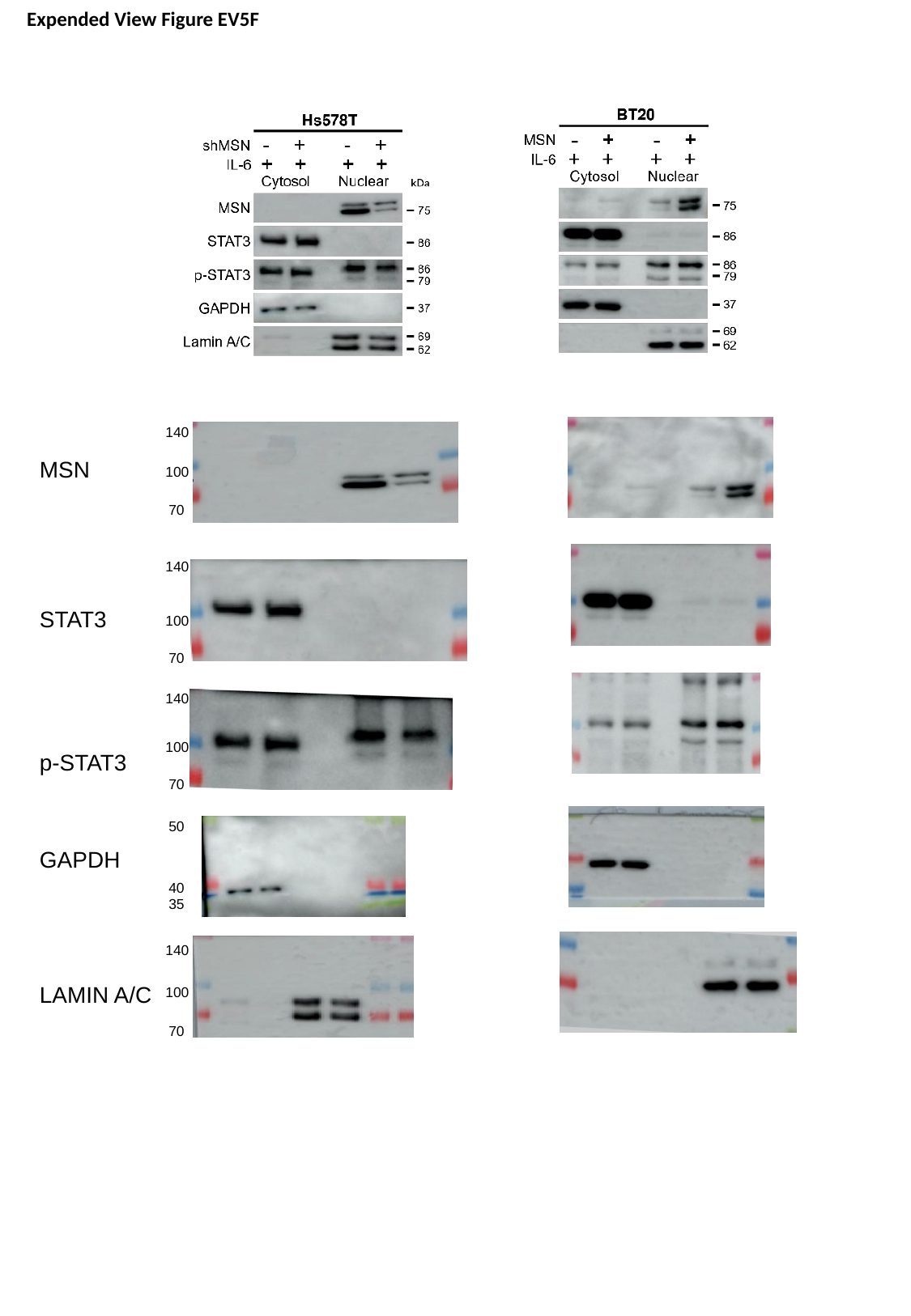

Expended View Figure EV5F
140
MSN
100
70
140
STAT3
100
70
140
100
p-STAT3
70
50
GAPDH
40
35
140
LAMIN A/C
100
70

## Slide 39
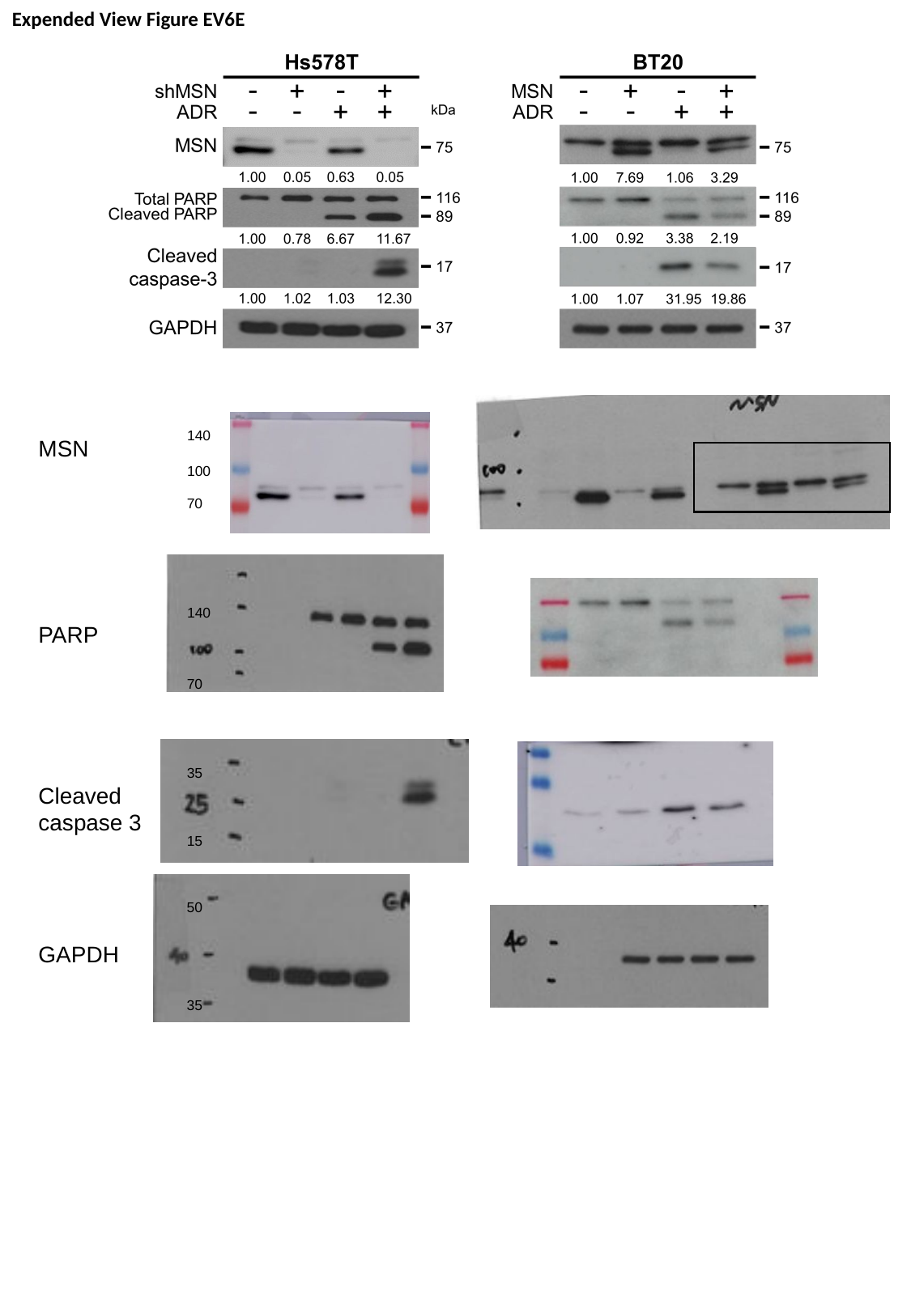

Expended View Figure EV6E
140
MSN
100
70
140
PARP
70
35
Cleaved caspase 3
15
50
GAPDH
35

## Slide 40
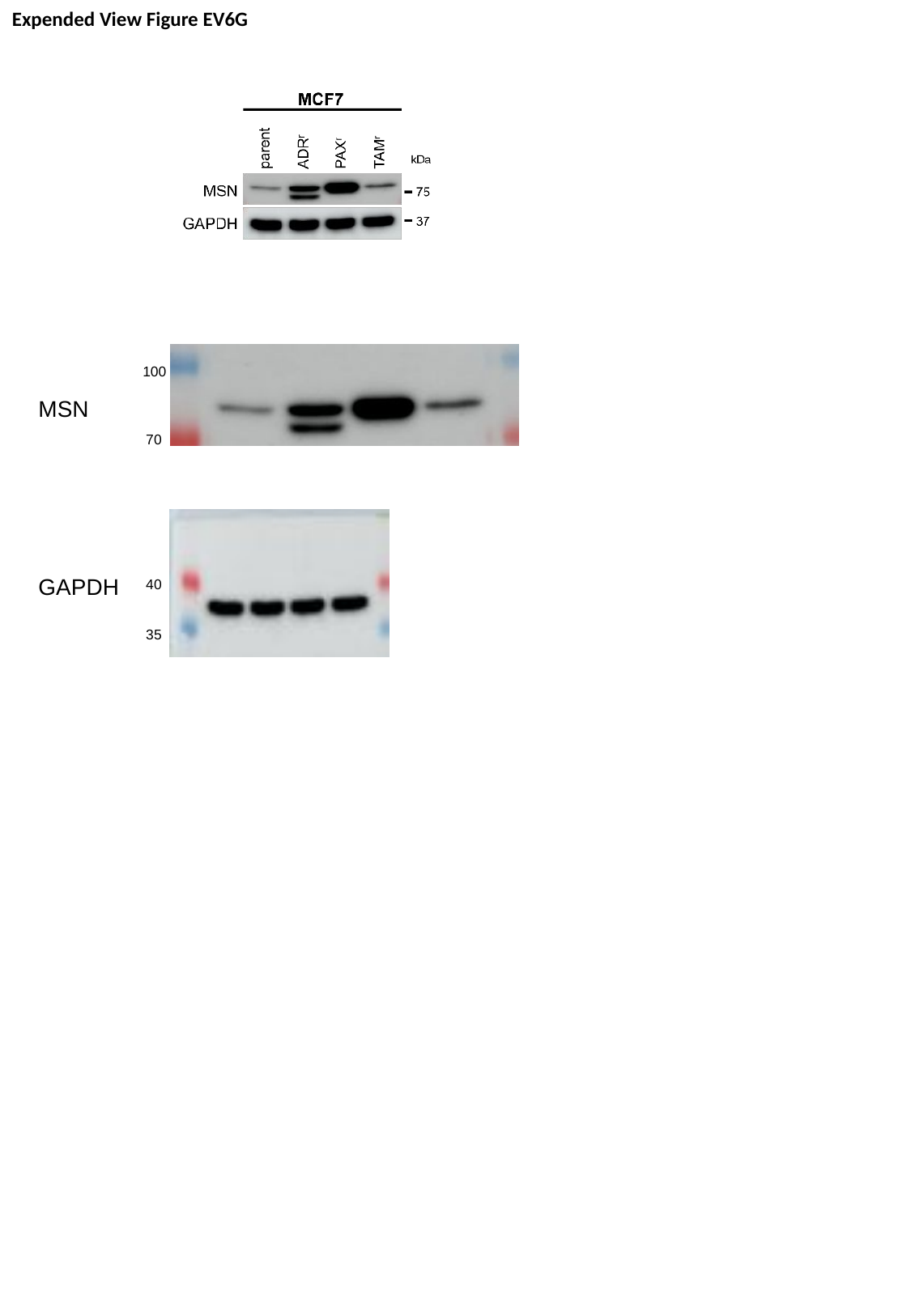

Expended View Figure EV6G
100
MSN
70
GAPDH
40
35

## Slide 41
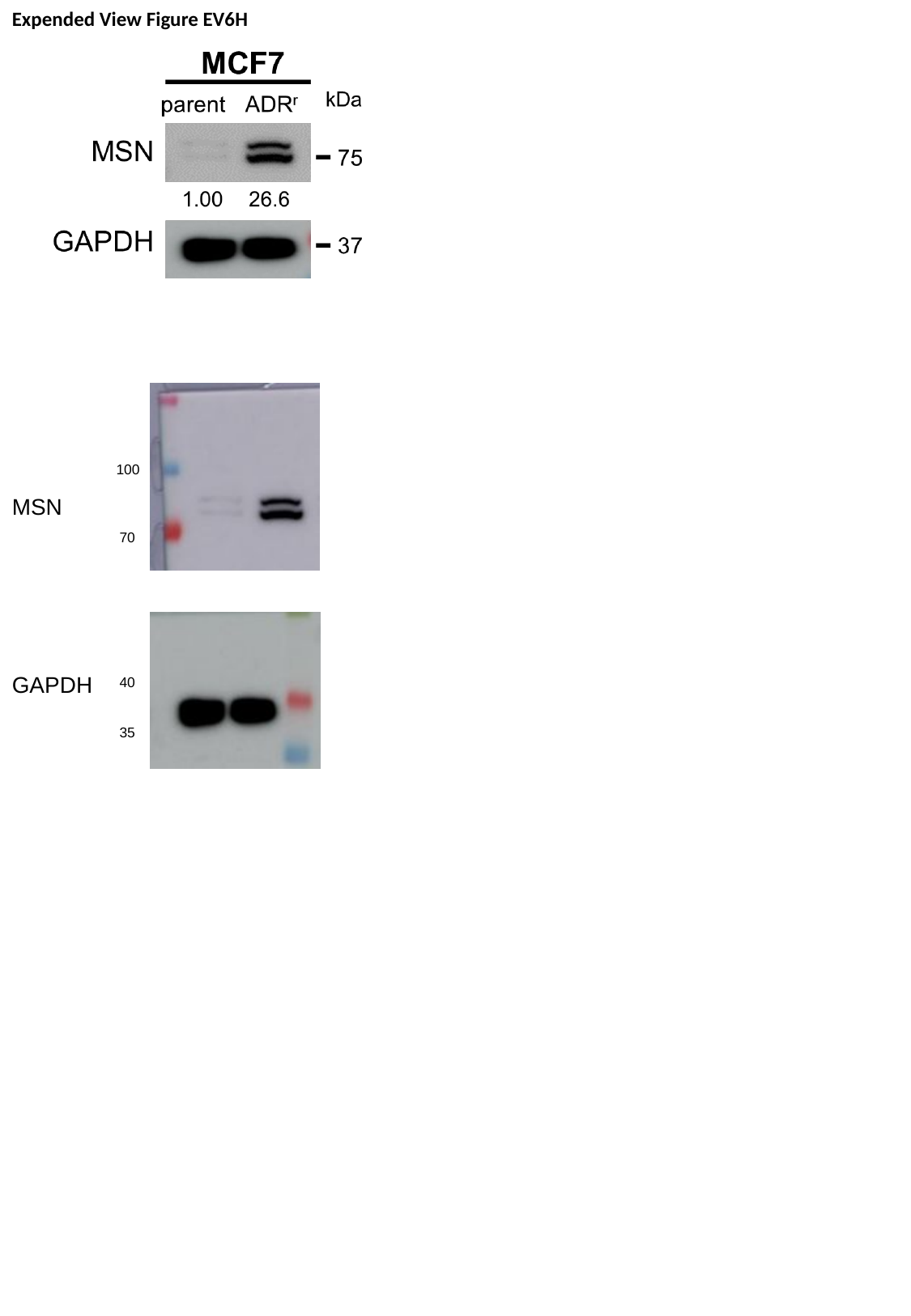

Expended View Figure EV6H
100
MSN
70
GAPDH
40
35

## Slide 42
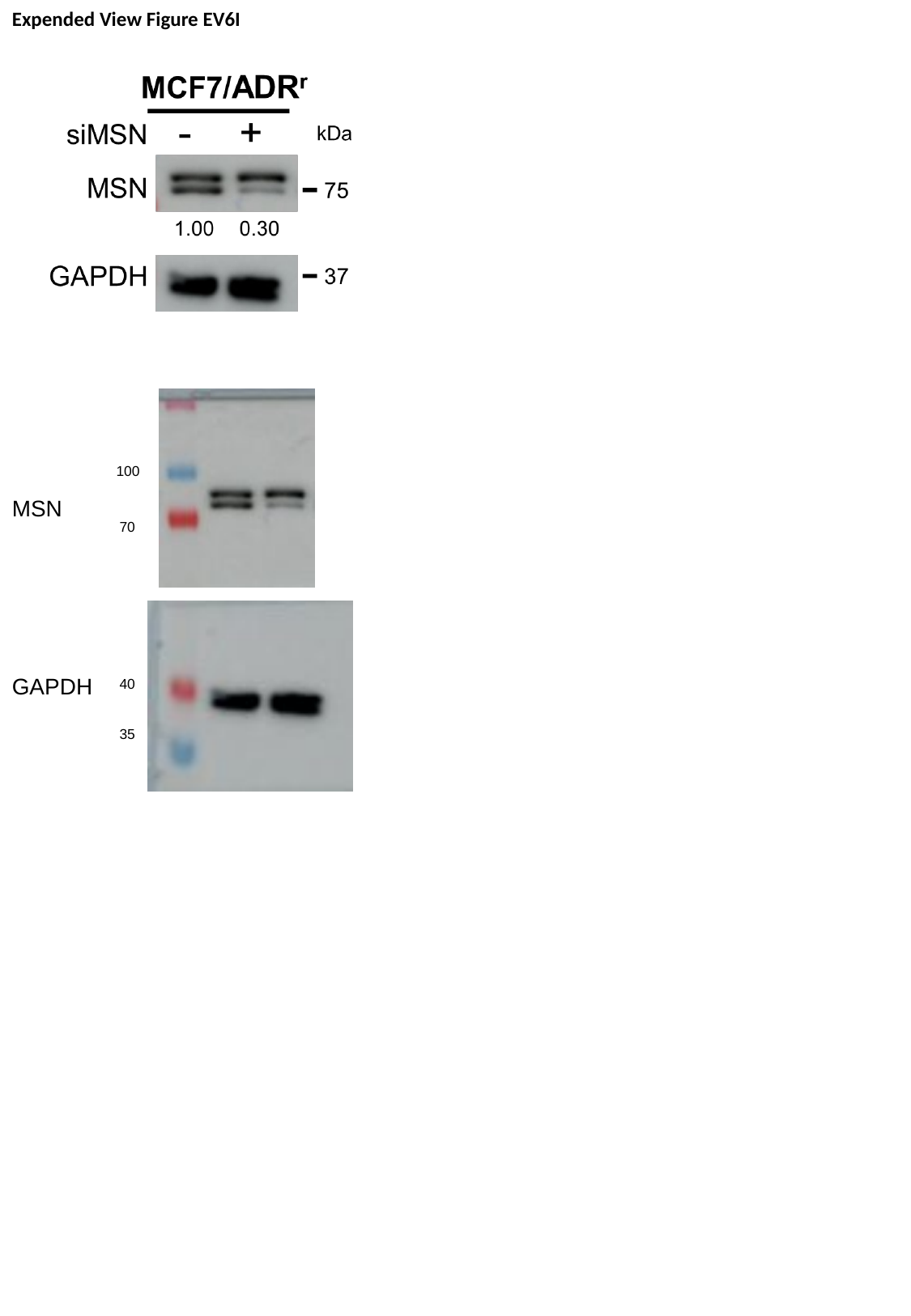

Expended View Figure EV6I
100
MSN
70
GAPDH
40
35

## Slide 43
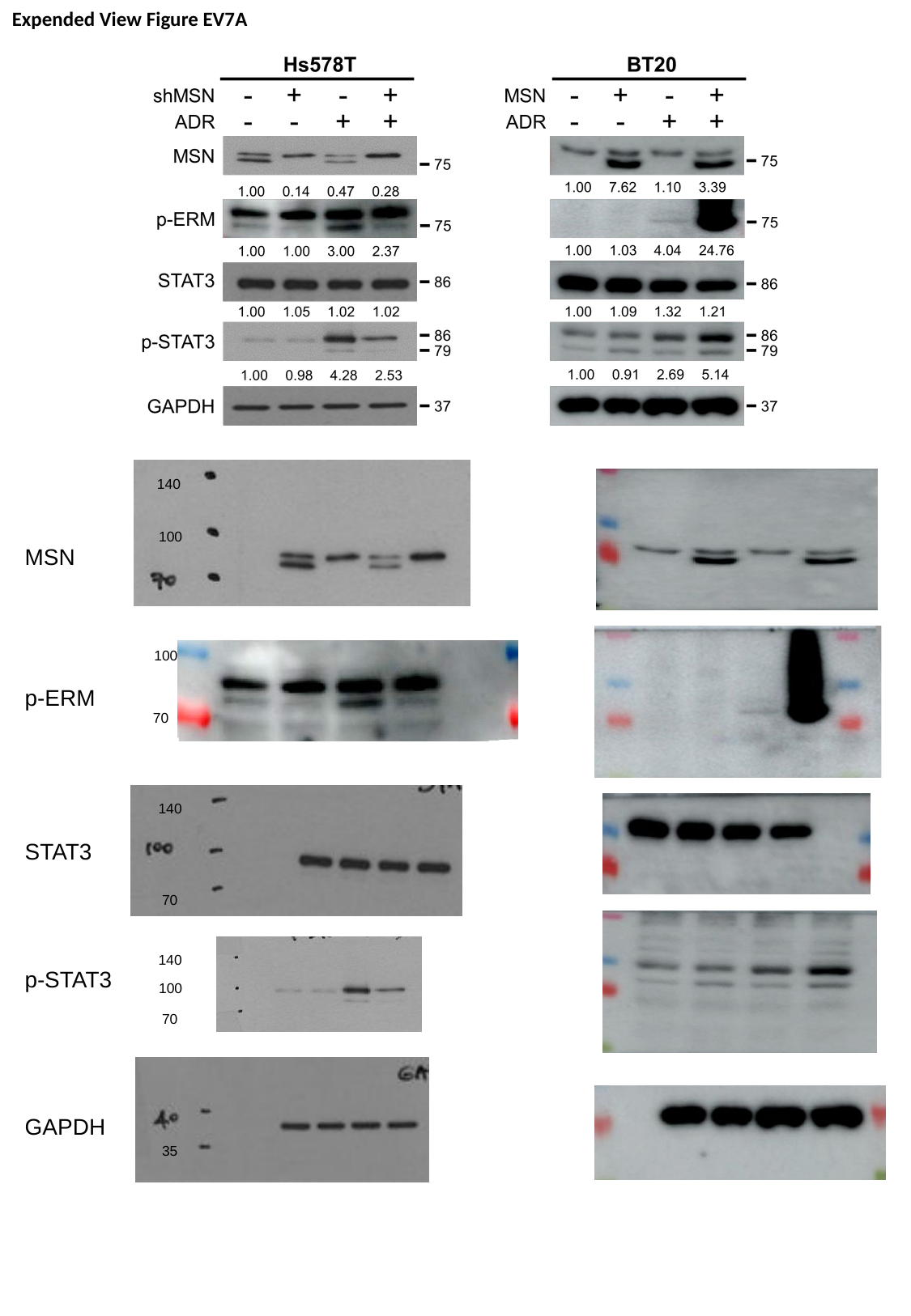

Expended View Figure EV7A
140
100
MSN
100
p-ERM
70
140
STAT3
70
140
p-STAT3
100
70
GAPDH
35

## Slide 44
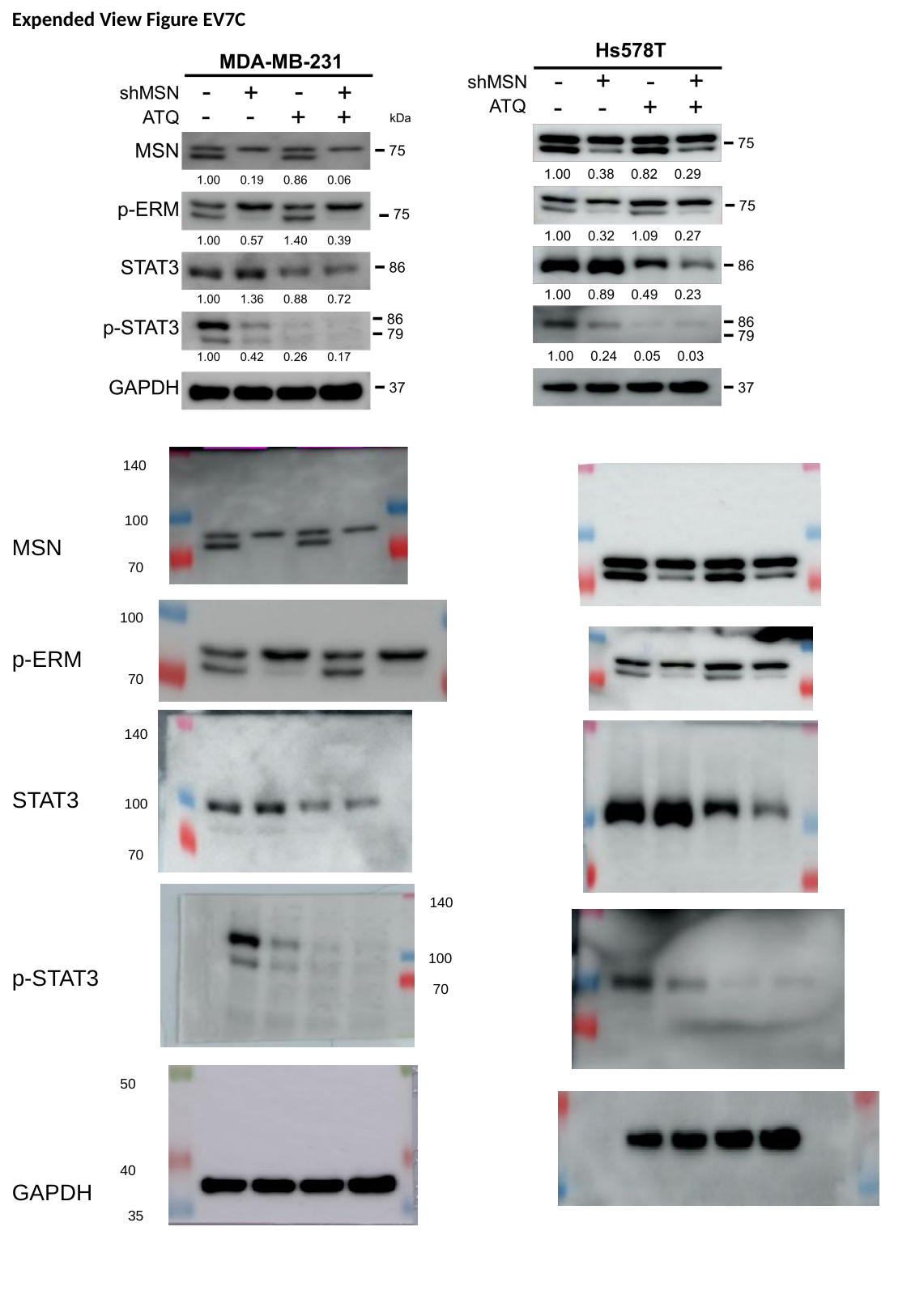

Expended View Figure EV7C
140
100
MSN
70
100
p-ERM
70
140
STAT3
100
70
140
100
p-STAT3
70
50
40
GAPDH
35

## Slide 45
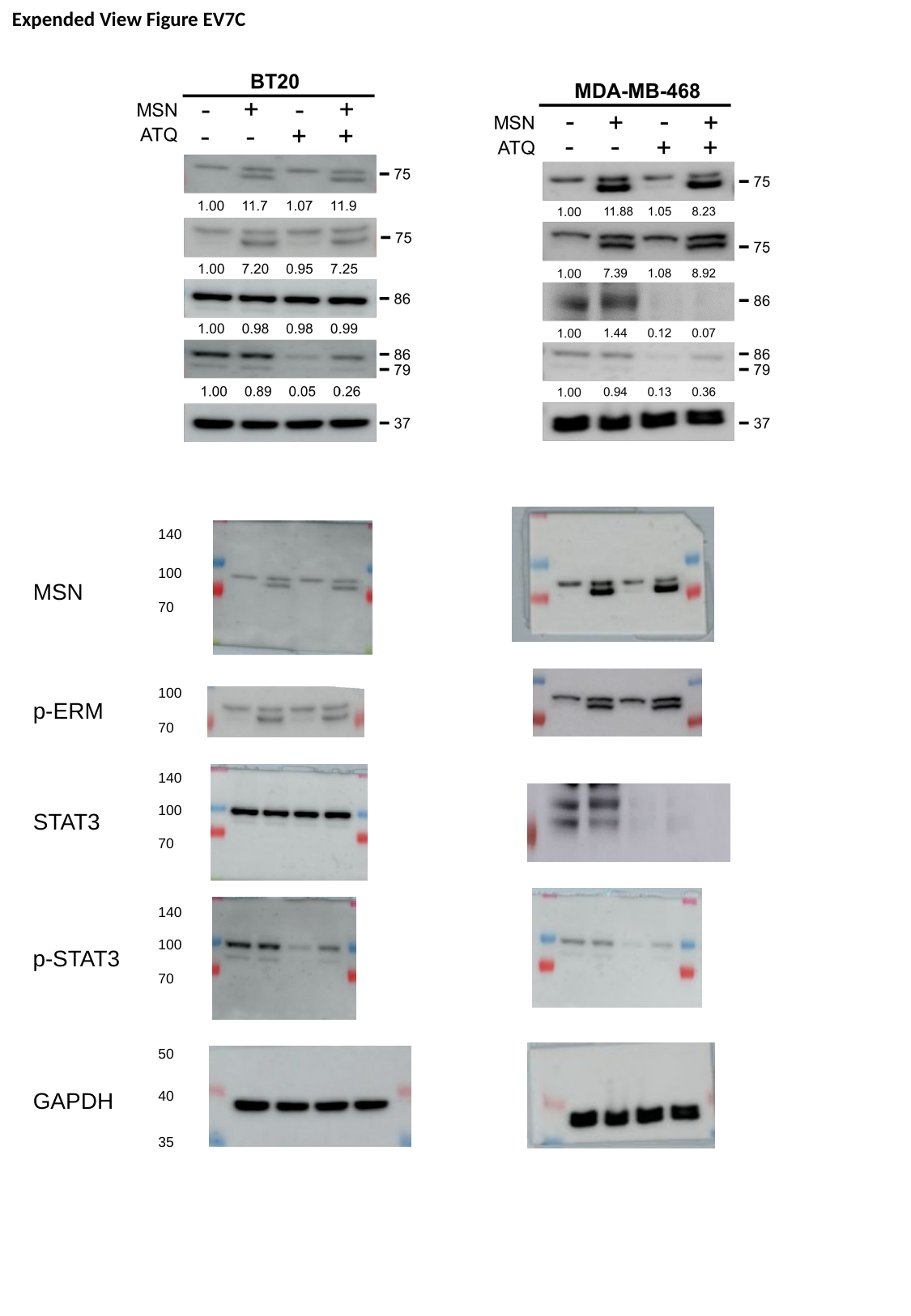

Expended View Figure EV7C
140
100
MSN
70
100
p-ERM
70
140
100
STAT3
70
140
100
p-STAT3
70
50
GAPDH
40
35
